# Perinatal and 2-year neurodevelopmental outcome in late preterm fetal compromise: the TRUFFLE 2 randomised trial protocol

**DOI:** 10.1136/bmjopen-2021-055543

**Published:** 2022-04-15

**Authors:** Bronacha Mylrea-Foley, Jim G Thornton, Edward Mullins, Neil Marlow, Kurt Hecher, Christina Ammari, Birgit Arabin, Astrid Berger, Eva Bergman, Amarnath Bhide, Caterina Bilardo, Julia Binder, Andrew Breeze, Jana Brodszki, Pavel Calda, Rebecca Cannings-John, Andrej Černý, Elena Cesari, Irene Cetin, Andrea Dall'Asta, Anke Diemert, Cathrine Ebbing, Torbjørn Eggebø, Ilaria Fantasia, Enrico Ferrazzi, Tiziana Frusca, Tullio Ghi, Jenny Goodier, Patrick Greimel, Wilfried Gyselaers, Wassim Hassan, Constantin Von Kaisenberg, Alexey Kholin, Philipp Klaritsch, Ladislav Krofta, Peter Lindgren, Silvia Lobmaier, Karel Marsal, Giuseppe M Maruotti, Federico Mecacci, Kirsti Myklestad, Raffaele Napolitano, Eva Ostermayer, Aris Papageorghiou, Claire Potter, Federico Prefumo, Luigi Raio, Jute Richter, Ragnar Kvie Sande, Dietmar Schlembach, Ekkehard Schleußner, Tamara Stampalija, Basky Thilaganathan, Julia Townson, Herbert Valensise, Gerard HA Visser, Ling Wee, Hans Wolf, Christoph C Lees, TRUFFLE 2 Collaborator Group, Abin Thomas

**Affiliations:** 1 Institute for Reproductive and Developmental Biology, Department of Metabolism, Digestion and Reproduction, Imperial College London, London, UK; 2 Department of Obstetrics and Gynaecology, University of Nottingham, City hospital, Nottingham, UK; 3 Elizabeth Garrett Anderson Institute for Women's Health University College London, London, UK; 4 Department of Obstetrics and Fetal Medicine, University Medical Center Hamburg-Eppendorf, Hamburg, Germany; 5 University College London Hospitals NHS Foundation Trust, London, UK; 6 Department of Obstetrics Charite, Humboldt University of Berlin, Berlin, Germany; 7 Department of Gynecology and Obstetrics, Medical University of Innsbruck, Innsbruck, Austria; 8 Department of Women’s and Children’s Health, Uppsala University, Uppsala, Sweden; 9 Fetal Medicine Unit, St George's University Hospitals NHS Foundation Trust, London, UK; 10 Department of Obstetrics Amsterdam, Vrije Universiteit Amsterdam, Noord-Holland, The Netherlands; 11 Department of Obstetrics and Fetomaternal Medicine, Medical University of Vienna, Vienna, Austria; 12 Fetal medicine Unit, Leeds Teaching Hospitals NHS Trust, Leeds, UK; 13 Department of Obstetrics and Gynecology, Lund Skanes universitetssjukhus Lund, Skåne, Sweden; 14 Department of Obstetrics and Gynaecology, Charles University, Praha, Czech Republic; 15 Centre for Trials Research, Cardiff University, Cardiff, UK; 16 Department of Obstetrics & Gynaecology, General University Hospital and First Faculty of Medicine, Charles University, Prague, Czech Republic; 17 Department of Obstetrics and Gynecology, Vittore Buzzi Hospital, University of Milan, Milan, Italy; 18 Medicine and Surgery, University of Parma, Parma, Italy; 19 Universitetet i Bergen, Bergen, Norway; 20 St Olav’s Hospital, Trondheim, Norway; 21 Unit of Fetal Medicine and Prenatal Diagnosis, RCCS materno infantile Burlo Garofolo Dipartimento di Pediatria, Trieste, Italy; 22 Department of Clinical Sciences and Community Health, Fondazione IRCCS Ca' Granda Ospedale Maggiore Policlinico, Milano, ltaly; 23 Department of Obstetrics & Gynecology, University of Parma, Parma, Italy; 24 Department of Obstetrics and Gynecology, Medical University of Graz, Graz, Austria; 25 Department of Obstetrics and Gynecology, Hospital Oost-Limburg, Genk, Belgium; 26 Obstetrics & Gynaecology, East Suffolk and North Essex NHS Foundation Trust, Colchester Hospital, Colchester, UK; 27 Obstetrics and Gynecology, Hannover Medical School, Hannover, Germany; 28 National Medical Research Center for Obstetrics, Gynecology & Perinatology, Moscow, Russia; 29 Division of Obstetrics and Maternal Fetal Medicine, Medical University of Graz, Graz, Austria; 30 Institute for Care of Mother and Child, Prague, Czech Republic; 31 Division of Obstetrics and Gynecology, Department of Clinical Science, Intervention & Technology (CLINTEC), Karolinska Institutet, Stockholm, Sweden; 32 Department of Obstetrics and Gynecology, Klinikum rechts der Isar, Technical University of Munich, Munich, Germany; 33 Obstetrics and Gynaecology, Faculty of Medicine, Lunds Universitet, Lund, Sweden; 34 Department of Neurosciences, Reproductive and Dentistry Sciences, Federico II University Hospital, Napoli, Italy; 35 High Risk Pregnancy Unit, University Hospital Careggi, Firenze, Italy; 36 Department of Obstetrics, Children's and Women's Health, St Olavs Hospital University Hospital, Trondheim, Norway; 37 Molecular & Clinical Sciences Research Institute, St George’s, University of London, London, UK; 38 Department of Obstetrics and Gynaecology, Università degli Studi di Brescia, Brescia, Italy; 39 Department of Obstetrics and Gynaecology, University of Bern, Bern, Switzerland; 40 Department of Gynecology and Obstetrics, Katholieke Universiteit Leuven, Leuven, Belgium; 41 Department of Obstetrics and Gynaecology, Stavanger University Hospital, Stavanger, Norway; 42 Vivantes Network for Health, Clinicum Neukoelln, Clinic for Obstetric Medicine, Berlin, Germany; 43 Klinik für Geburtsmedizin, Universitätsklinikum Jena, Jena, Germany; 44 Division of Obstetrics and Gynaecology Policlinico Casilino, Roma, Italy; 45 Department of Obstetrics, University Medical Center, Utrecht University, Utrecht, The Netherlands; 46 Obstetrics And Gynaecology, Princess Alexandra Hospital NHS Trust, Harlow, UK; 47 Department of Obstetrics and Gynaecology, Amsterdam University Medical Centres, Amsterdam, The Netherlands

**Keywords:** fetal medicine, maternal medicine, ultrasonography

## Abstract

**Introduction:**

Following the detection of fetal growth restriction, there is no consensus about the criteria that should trigger delivery in the late preterm period. The consequences of inappropriate early or late delivery are potentially important yet practice varies widely around the world, with abnormal findings from fetal heart rate monitoring invariably leading to delivery. Indices derived from fetal cerebral Doppler examination may guide such decisions although there are few studies in this area. We propose a randomised, controlled trial to establish the optimum method of timing delivery between 32 weeks and 36 weeks 6 days of gestation. We hypothesise that delivery on evidence of cerebral blood flow redistribution reduces a composite of perinatal poor outcome, death and short-term hypoxia-related morbidity, with no worsening of neurodevelopmental outcome at 2 years.

**Methods and analysis:**

Women with non-anomalous singleton pregnancies 32+0 to 36+6 weeks of gestation in whom the estimated fetal weight or abdominal circumference is <10th percentile or has decreased by 50 percentiles since 18–32 weeks will be included for observational data collection. Participants will be randomised if cerebral blood flow redistribution is identified, based on umbilical to middle cerebral artery pulsatility index ratio values. Computerised cardiotocography (cCTG) must show normal fetal heart rate short term variation (≥4.5 msec) and absence of decelerations at randomisation. Randomisation will be 1:1 to immediate delivery or delayed delivery (based on cCTG abnormalities or other worsening fetal condition). The primary outcome is poor condition at birth and/or fetal or neonatal death and/or major neonatal morbidity, the secondary non-inferiority outcome is 2-year infant general health and neurodevelopmental outcome based on the Parent Report of Children’s Abilities-Revised questionnaire.

**Ethics and dissemination:**

The Study Coordination Centre has obtained approval from London-Riverside Research Ethics Committee (REC) and Health Regulatory Authority (HRA). Publication will be in line with NIHR Open Access policy.

**Trial registration number:**

Main sponsor: Imperial College London, Reference: 19QC5491. Funders: NIHR HTA, Reference: 127 976. Study coordination centre: Imperial College Healthcare NHS Trust, Du Cane Road, London, W12 0HS with Centre for Trials Research, College of Biomedical & Life Sciences, Cardiff University. IRAS Project ID: 266 400. REC reference: 20/LO/0031. ISRCTN registry: 76 016 200.

**Strengths and limitations of this study**

- Changes in cerebral Doppler measures are considered a strong candidate for triggering delivery following detection of late preterm fetal growth restriction.
- The primary outcome is a composite of perinatal and neonatal outcome, and infants will be followed to determine health and neurodevelopment at 2 years to ensure safety and non-inferiority of intervention.
- This appropriately powered UK and European multicentre trial will inform NHS policy and the findings will be generalisable to late preterm fetal growth restriction in other healthcare systems.
- There are limited data available in respect of selecting the threshold of cerebral Doppler change for randomisation.
- Some non-UK centres will not randomise women at the earlier gestation range (32–33+6 weeks) and this could limit the generalisability of the results in this group of women.

## Introduction

Third trimester poor fetal growth and compromise are strongly associated with stillbirth, neonatal illness^1^ and an increased risk of fetal or neonatal brain injury.^2^ The only therapeutic option is delivery of the fetus. This poses a dilemma: delivery too early may incur the complications of prematurity, delivery too late risks further fetal compromise, brain injury and late stillbirth. The problem of when to deliver these babies is twofold: there is no consensus on how to identify fetal compromise and there is no ‘ideal’ evidence-based monitoring strategy. Current screening strategies include standardised symphysis-fundal height measurement, third trimester ultrasound and umbilical Doppler velocimetry.^3 4^ National Institute for Health and Care Excellence (NICE) acknowledges that methods to identify fetal growth restriction (FGR) are ‘poorly developed or not tested by rigorous methodology’.

The use of Doppler ultrasound of fetal vessels allows non-invasive assessment of blood flow. Fetal ‘cerebral blood flow redistribution’, prioritising blood flow toward the brain at the expense of other organs, is a response to an adverse intrauterine environment characterised by hypoxaemia. Ultrasound markers of such fetal compromise include Doppler assessment of blood flow velocity^5–7^ and abdominal circumference growth velocity ‘drop off’ in the third trimester.^8 9^ Intervention by delivery of the fetus in response to either evidence of blood flow redistribution or fetal growth slowing has never been tested in a randomised trial. Computerised cardiotocography (cCTG) has been used in several observational studies to determine fetal condition in relation to hypoxaemia and acidosis. The RCOG (Royal College of Obstetricians and Gynaecologists) recommends that research is required to evaluate the effectiveness of third trimester ultrasound assessment, concluding that ‘middle cerebral artery (MCA) Doppler may be a more useful test in small for gestational age (SGA) fetuses detected after 32 weeks’^4^ but does not define parameters that should trigger delivery. Even with effective screening for FGR, the questions of how to monitor and when to deliver would remain.

A Cochrane review of the management of ‘compromised babies’ at term showed no difference in perinatal or long-term outcome with a policy of early delivery versus conservative management.^10^ Only three trials were included: two included small babies, both part of the DIGITAT (Disproportionate Intrauterine Growth Intervention Trial At Term) study (a small pilot and the main trial),^11^ the third included babies with reduced amniotic fluid. There is no Cochrane review on the optimal timing of delivery in late preterm growth restricted babies.

A systematic review included only one trial of timed delivery in late preterm babies. The Growth Restriction Intervention Trial^12^ included 547 babies from 24 to 36 weeks of gestation with evidence of preterm growth restriction or compromise, where there was clinical uncertainty whether immediate delivery was indicated. Of these, 210 babies were recruited between 33+0 and 36+6 weeks: 107 women were randomised to early delivery and 103 to delayed delivery. Mortality and a range of neurodevelopmental measures were similar between the groups. These results cannot be used to inform management because of the small number of infants assessed using only umbilical artery Doppler velocimetry, the subjective assessment of the cardiotocograph (ie, not computerised analysis), and the use of clinician’s discretion rather than standardised management.

Results from our previous randomised trial of delivery decision-making using Doppler velocimetry and cCTG in pregnancies with FGR at 26–32 weeks of gestation—the TRial of Umbilical Fetal FLow in Europe (TRUFFLE) provided evidence associating monitoring strategies with improved outcomes^13^ and guides practice internationally.^14 15^ In TRUFFLE (2005–2010) we studied FGR <32 weeks of gestation using cCTG as the standard of care for timing delivery, compared with early or late fetal ductus venosus (DV) Doppler velocimetry changes with a cCTG ‘safety net’. Surviving infants whose mothers had been randomised to delivery in late DV changes arm showed better neurodevelopment at 2 years of age.^13^

We then carried out the prospective multicentre observational TRUFFLE 2 Feasibility Study (2017–2018, n=1024). A range of markers of cerebral redistribution were evaluated as potential delivery trigger points in two gestational age bands. These included the MCA Doppler and the umbilical cerebral ratio (UCR); calculated by dividing the Doppler pulsatility index of the umbilical artery by that of the MCA. Participating clinicians suggested greater concern about fetal condition is required to trigger delivery at earlier rather than later gestational ages between 32 and 36 weeks. We, therefore, selected a more abnormal threshold for cerebral redistribution at earlier gestational ages, based on stepped UCR z-score values.^16^ Of note was the finding from a prospective study that birth condition, fetal mortality and neonatal morbidity rate were more common among fetuses showing higher UCR z-score, indicating cerebral redistribution, compared with those with a lower UCR z-score (15 vs 9%).^17^

On this basis, we designed this randomised trial of delivery for women identified with late preterm FGR, to test the hypothesis that delivery based on UCR values derived from the feasibility study will safely lead to improved fetal outcomes (TRUFFLE 2).

## Methods and analysis

### Study design

This is an individual unblinded randomised trial of pregnant women experiencing FGR. Following informed consent, 1560 women will be randomised to either immediate or delayed delivery. Women are being recruited from UK and European centres.

Potential participants are women with singleton non-anomalous pregnancies between 32+0 and 36+6 weeks of gestation in whom a SGA fetus is identified or one whose growth has slowed. This is defined as estimated fetal weight or abdominal circumference <10th percentile and/or having decreased by 50 percentiles since an ultrasound scan at 18–32 weeks. Biometry must have been performed in the 14 days prior to inclusion. Each centre will use local growth charts. Data is collected on absolute measurements and growth chart-derived percentiles. Once identified as potential participants women receive regular monitoring as per local standard of care for fetal condition, using ultrasound biometry, umbilical and MCA Doppler velocimetry assessments and cCTG (using Dawes-Redman criteria). This is recommended to be repeated every 14 days; these observational data are recorded.

Women become eligible for randomisation when signs of fetal cerebral blood flow redistribution are detected by Doppler, defined below and shown in the study flow chart (online supplemental file 1—flow chart).

10.1136/bmjopen-2021-055543.supp1 Supplementary data

Delivery is based on UCR pulsatility index z-scores, of >1.5 (between 32+0 and 33+6 weeks) or >1.0 (34+0 to 36+6 weeks). These correspond to an absolute UCR of ≥1.0 at 32+0 to 33+6 weeks and ≥0.8 at 34+0 to 36+6 weeks. Abnormal UCR measurements must be repeated within 15 minutes–24 hours to confirm these values. This need not be consecutive, so that if the second measurement is normal the patient may still be randomised if a repeat Doppler measurement is abnormal within 72 hours of the first abnormal measurement. Randomisation will occur at the time of the second qualifying UCR measurement and is stratified by centre and gestational age. Randomisation is through the CASTOR website (Amsterdam, NL) into which eligibility, monitoring and outcome data are entered.

Women are consented in a two-stage process. Stage 1 (pre-eligible) consent is not a prerequisite and women may be consented directly for randomisation with stage 2 (eligible for randomisation) consent.

#### Pre-eligible: consent for observational data collection

Consented for prospective data collection once identified as meeting criteria for SGA or slowed fetal growth (as defined above) but not meeting cerebral Doppler thresholds for randomisation or not willing to be randomised. This will include demographics, medical history, ultrasound findings and outcomes. This consent will include obtaining a personal email address, which is entered into and stored on Castor, and willingness to be contacted in the future for follow-up where women are randomised (online supplemental file 2—consent form for observational data collection).

10.1136/bmjopen-2021-055543.supp2 Supplementary data

#### Eligible for randomisation: consent for randomisation

Consent for randomisation once cerebral redistribution is identified, with UCR (as defined above) of ≥1.0 at 32+0 to 33+6 weeks and ≥0.8 at 34+0 to 36+6 weeks. This consent will also include contact details and willingness to be contacted in the future for follow-up data collection (online supplemental file 3—consent for randomisation).

10.1136/bmjopen-2021-055543.supp3 Supplementary data

Women are randomly allocated to either immediate delivery or delayed delivery as defined below. Randomisation has a 1:1 allocation ratio and stratified based on gestational age (above or below 34 weeks) and centre. Randomisation is conducted on the electronic data capture platform (Castor EDC, Amsterdam, The Netherlands).

#### Immediate delivery

Participants in the immediate delivery arm will be delivered by Caesarean or induction of labour will be commenced within 48 hours, allowing for administration of corticosteroids and infusion of magnesium sulphate as per local protocol and guidance. Start of induction of labour is defined as administering cervical preparation (cervical balloon, prostaglandins, etc), artificial rupture of membranes or administration of oxytocin.

#### Delayed delivery

Participants in the delayed delivery arm will be monitored using twice weekly Doppler and cCTG monitoring, or more frequently based on local clinical protocols if required. Umbilical artery Doppler velocities may be measured in this time and delivery may be based on these safety net criteria (see box below). We strongly recommend that MCA measurements are not undertaken during monitoring in the delayed delivery arm. Delivery is indicated when short term variation (STV) is <4.5 ms on cCTG or there are repeated fetal heart rate decelerations. Once participants reach 37+0 weeks of gestation the delivery plan will be based on local protocols.

Box 1 **Delivery thresholds based on umbilical artery Doppler for participants in all arms of the study**

Umbilical Doppler delivery thresholds.

In all arms absolute indications for delivery include:

- Umbilical artery Doppler with reversed end diastolic flow after entry into the trial, OR
- Umbilical artery Doppler absent end diastolic flow from 34+0 weeks.

### Study timeline

Study setup: 0–10 months; recruitment/randomisation: 11–36 months. Two years follow-up questionnaires 35–60 months; analyses, writing up, reporting and dissemination: 61–66 months. This equates to 5 and ½ years (66 months).

### Study outcome measures

#### Primary outcome

The primary outcome for the study is a composite of fetal or infant death, composite measure of poor condition at birth and neonatal morbidity—as defined by presence of any of the following:

1. Poor condition at birth
  - Apgar score at 5 min <7, umbilical artery pH <7.0 or umbilical vein pH <7.1.
  - Need for resuscitation with intubation, chest compressions or medication.
2. Fetal death/ death before neonatal hospital discharge
3. Neonatal brain injury syndromes
  - Infants with a diagnosis consistent with hypoxic ischaemic encephalopathy (HIE): term and near-term infants only.
  - Infants with a diagnosis of intracranial haemorrhage, perinatal stroke, HIE, central nervous system infection, or kernicterus (bilirubin encephalopathy): all infants.
  - Preterm white matter disease (periventricular leukomalacia): preterm infants only.
  - Infants with a recorded seizure confirmed by Electro-encephalogram (EEG).
4. Respiratory support
  - Need for mechanical support of respiration after admission to neonatal unit (NNU), for more than 1 hour; includes need for continuous positive airways pressure (or NIPPV; Noninvasive positive-pressure ventilation) or mechanical ventilation via endotracheal tube but excludes need for supplemental oxygen.
5. Cardiovascular abnormality
  - Hypotensive treatment, patent ductus arteriosus requiring treatment, or disseminated coagulopathy.
6. Sepsis
  - Clinical sepsis with positive blood culture.
  - necrotising enterocolitis requiring surgery.
7. Retinopathy of prematurity requiring treatment (laser or anti Vascular Endothelial Growth Factor (VEGF) injections)

#### Secondary outcomes

##### For the baby

Health and developmental outcomes—assessed using Parent Report of Children’s Abilities-Revised (PARCA-R) questionnaire at 2 years corrected age and an Infant Health Questionnaire up to 2 years (online supplemental file 4—PARCA-R Questionnaire and online supplemental file 5—Infant Health Questionaire).

10.1136/bmjopen-2021-055543.supp4 Supplementary data

10.1136/bmjopen-2021-055543.supp5 Supplementary data

The PARCA-R^18^ will be completed at 24 months term-equivalent age, this allows derivation of the non-verbal cognitive scale and language development scale. Raw scores from the scales are standardised (by corrected age and gender) to a notional population mean of 100 (SD=15) and the average of these two component scores will be taken as the overall composite score. Corrected age is used for preterm babies (born before 37 weeks) and represents the age of the child from the estimated date of delivery.

The Infant Health Questionnaire^19^ will be used to derive the following health outcomes at 6, 12, 18 and 24 months post partum:

- Use of any hospital service (yes/no) and total number of contacts over the 2-year period.
- Admitted to hospital (yes/no) and total number of admissions over the 2-year period.
  - Planned/unplanned admissions to hospital (yes/no) over the 2-year period.
  - Intensive care or not over the 2-year period.
- Attended emergency department (and not subsequently admitted) (yes/no) over the 2-year period.
- Attended Outpatients/clinic (yes/no) over the 2-year period.

##### For the mother

1. Gestational hypertension developed post study entry
  - As defined by the International Society for Study of Hypertension in pregnancy (ISSHP):^20^ hypertension (blood pressure ≥140/90 mm Hg) arising de novo after 20 weeks gestation in the absence of proteinuria.
2. Pre-eclampsia developed post study entry
  - As defined by the ISSHP:^20^ blood pressure ≥140/90 mm Hg and significant proteinuria (protein/creatinine ratio of 30 mg/mmol or more).
3. Onset of labour (spontaneous, induction (method), prelabour caesarean section).
4. Mode of delivery (spontaneous vaginal, assisted vaginal, caesarean section).

### Doppler quality control

#### Sonographer standardisation

Each sonographer in each centre taking part in the trial are assessed by the local principal investigator (PI). Each sonographer will submit to the local PI two images: pseudo anonymised ultrasound images for each Doppler parameter (umbilical artery and MCA) each showing a colour Doppler image with the gate placed over the vessel, and the pulsed wave Doppler waveform arising from that image. The local PI will determine whether these images are satisfactory using a predefined quality control scoring system (online supplemental file 6—Doppler image scoring sheet).

10.1136/bmjopen-2021-055543.supp6 Supplementary data

#### Doppler ultrasound criteria

Measurements are obtained in fetuses between 32+0 and 36+6 weeks of gestation. Umbilical artery and MCA pulsatility index images are collected according to specific predefined objective criteria for both the colour Doppler images and pulsed wave Doppler.

#### Doppler quality control

The local PI will provide details of all sonographers having undergone standardisation in that centre to the Centre for Trials Research (CTR). The CTR will independently request all images submitted to the local PI for the first five patients randomised from each unit, and then for up to 10% of patients thereafter, for anonymised quality control assessment by the Quality Control Board. All images are collected as pseudo anonymised.jpeg images and saved electronically in a Doppler ultrasound sonographer standardisation file by the PI, with the submitting sonographer identifiable. Images are scored using the predefined scoring criteria. The CTR will manage this process and will provide feedback if necessary, and ensure that members of the TRUFFLE 2 Quality Control Board do not assess images from their own unit.

### Participant entry

#### Preregistration evaluations

- Ultrasound scan of fetal growth between 30+0 and 36+6 weeks of gestation, including measurement of MCA and umbilical artery Doppler velocities.
- cCTG STV analysed using Dawes-Redman criteria.

#### Inclusion criteria

(All criteria should be fulfilled to be eligible for randomisation)

- Women ≥18 years old.
- Pregnant with singleton non-anomalous fetus.
- Between 32+0 and 36+6 weeks of gestation.
- Estimated fetal weight or abdominal circumference <10 th percentile OR decreased by 50 percentiles since an ultrasound scan at 18+0–32+0 weeks.
- Cerebral redistribution defined as UCR ≥1.0 (between 32+0 and 33+6 weeks) or ≥0.8 (34+0 to 36+6 weeks) repeated within 15 minutes–24 hours.
- Normal STV on cCTG (4.5 msec or above).

#### Exclusion criteria

- Indication for immediate delivery required within 48 hours.
- Unable to give informed consent.
- Preterm prelabour rupture of the membranes.
- Suspected placental abruption or antepartum haemorrhage.
- Presence of reversed end diastolic flow in the umbilical artery.

### Assessment and follow-up

Assessment of the primary outcome is at infant discharge from the NNU and assessment of the key secondary infant outcomes will use the Infant Health Questionnaire and PARCA-R. The Infant Health Questionnaire will be sent out by investigators via Castor at 6, 12, 18 and 24 months post partum. Neurodevelopment is assessed at 2 years age corrected for prematurity using the PARCA-R, also sent via Castor (translated into local language). The window for determining 2-year outcome is from 23.5 to 27.5 months over which range the PARCA-R has been standardised.

Endpoint is 30 months after the estimated date of delivery of the last participant to deliver (24 months follow-up with additional 6 months for data cleaning and additional enquiries).

### Statistics and data analysis

#### Sample size

The trial is powered to detect if immediate delivery following cerebral redistribution is superior to expectant management following cerebral redistribution on this outcome. A difference in the proportion with the primary outcome from 15% in the delayed delivery to 9% in the immediate delivery (from TRUFFLE 2 feasibility study) demonstrates an OR of 0.56. At two-sided 5% significance with 95% power, 780 participants per arm are required, giving 1560 in total. Given the immediacy of this outcome, no loss to follow-up is expected. An important non-inferiority secondary safety outcome is infant neurodevelopment, which is measured by parent completed questionnaire at 2 years using the PARCA-R, as recommended in NICE Guidance,^21^ supplemented by infant health information over the intervening 2 years. Assuming a loss to follow-up at 2 years of 20%, 2-year outcomes for approximately 1248 infants are expected (624 per group assuming no difference in the lost to follow-up between the groups). The PARCA-R questionnaire provides a composite score for neurodevelopment with a standardised mean of 100 and SD of 15. With a one-sided significance level of 1%, under a non-inferiority hypothesis, a sample size of 624 in each group achieves a 98% power to detect a non-inferiority margin of difference in the mean PARCA-R score of no less than four points (0.25 of a SD). A margin of no less than three points can be detected with 90% power.

#### Main analysis

The primary analysis approach for the primary outcome of composite of adverse fetal/neonatal outcomes, maternal secondary outcomes and the Infant Health Questionnaire will be intention to treat with participants analysed in the groups to which they are assigned regardless of deviation from the protocol or intervention received. The PARCA-R will be both an intention to treat and a per protocol analysis, since the hypothesis under examination for these outcomes is a non-inferiority hypothesis. The per-protocol analysis will exclude babies of women who do not receive their intervention as planned. As the trial includes multiple centres (and will involve a reasonable number of participants randomised per centre), the analysis will be based on the individual participant, allowing for clustering between participants within centre using robust SEs. All analyses will additionally adjust for gestational age at inclusion (stratification risk factor used in randomisation) as a fixed factor. For binary outcomes (composite of adverse fetal/neonatal outcomes, gestational hypertension, pre-eclampsia) a logistic regression model will be used to compare this outcome by arm and results presented as ORs and two-sided 95% CI. Continuous outcomes (PARCA-R, Infant Health Questionnaire) will be analysed using linear regression and results presented as adjusted differences in means alongside 95% CIs. The Infant Health Questionnaire will also be examined over time using a repeated measures model, and will include an interaction term for time (6, 12, 18 and 24 months) and trial arm to investigate any divergent or convergent pattern in Infant Health Questionnaire. The categorical outcomes of mode of delivery and onset of labour will be compared between trial arms by fitting a multilevel ordinal regression model. The neonatal secondary outcome will be examined using the mean PARCA-R score between each trial arm using linear regression, and a one-sided 95% CI constructed to assess non-inferiority. Additional pre-specified sub-group analyses will be carried out to analyse the primary outcome in the whole cohort by those that are <10th percentile (vs ≥10th) and <3rd percentile (vs ≥3rd) based on different growth charts, maternal morbidity (yes/no) and corticosteroid administration (yes/no).^22 23^ If numbers are sufficient, we will also describe the intervention effect in the subgroups <3rd percentile versus 4–10th vs >10th percentile but will not statistically test for intervention effect.

All analyses will be undertaken after database lock following data collection at 2 years. No interim analyses are planned. Missing outcome data but will be accounted for in sensitivity analyses using multiple imputation, where we will assume that outcome data are missing at random given the observed measurements. All planned analyses will be described in detail in a statistical analysis plan, which will be finalised prior to database lock. The reporting of findings will be in accordance with the Consolidated Standards of Reporting Trials guidelines for Randomized Controlled Trials (RCTs). Statistical analysis will be performed in Stata (V.16 or higher).

### Data collection

Data collection and randomisation are carried out on a secure cloud-based electronic data capture platform (Castor EDC, Amsterdam, The Netherlands). Participants can only be identified by their recruiting centres using unique trial identifiers. Participant email addresses will be stored for sending follow-up questionnaires within an encrypted section of Castor only their recruiting centre has access to.

### Trial monitoring

A Trial Management Group will meet monthly to review study progress and recruitment targets. In addition there will be a Trial Steering Committee and Data Monitoring Committee, with a majority of independent members, to guarantee the safety of study participants.

A clinical trial risk assessment has been developed by CTR at Cardiff University, to determine the intensity and focus of central and on-site monitoring activity in the TRUFFLE 2 trial. Appropriate monitoring levels will be employed and are fully documented in the trial monitoring plan. Investigators should agree to allow trial related monitoring, including audits and regulatory inspections, by providing direct access to source data/documents as required. Findings generated from on-site and central monitoring will be shared with the Sponsor, Chief Investigator (CI), PI and local Research & Development (R&D) departments.

Serious adverse events (SAEs) will be reported for randomised participants and their infants. The reporting period is from the point of randomisation until discharge home for the mother and neonate, maternal death is to be reported up to 42 days after delivery. SAEs will be reported on the data capture platform (Castor) in accordance with the UK policy framework for Health and Social care research.

### Patient and public involvement

Patient and public involvement (PPI) has been embedded throughout the development of this study.

SANDS, the Stillbirth and Neonatal Death charity in the UK, has been closely involved in the development and design of this study. SANDS is clear that there is an urgent need for better assessment and care of women and their babies in late pregnancy and are fully supportive of the study. They will play an integral role throughout the study and in publicising the study and disseminating results in accessible formats. The research and development lead at SANDS is a member of the Trial Management Group. In their role, they represent members of the public affected by stillbirth and neonatal death.

An FGR PPI panel was convened to discuss TRUFFLE 2 study design. The purpose of the workshop was to ask members of the public for their views on the study, with specific focus on the concept of randomisation, the current situation for the treatment of FGR, and the scope of this study. The FGR panel comprised eight women who had experienced FGR, stillbirth or uncomplicated pregnancies. At the workshop the proposed study design was discussed. Following the workshop, the women were asked to complete an anonymous questionnaire relating to early delivery compared with monitoring the health of the baby in the womb and randomisation. Overall, women were supportive of the study, one stated ‘I believe a lot of women will benefit from this study’. Another stated ‘The study will have an impact; it will act as a ripple effect on the family and wider public for the better.’ Regarding randomisation, most women said that they would be happy to be randomised to either arm while acknowledging that they would need support, one woman stated ‘Yes, I would be randomised but would need the right support from the clinical staff. I would need to have 100% trust in my doctors’. Two women from this panel have agreed to be involved in the management of the study as independent members of the Trial Steering Committee. We incorporated the views and feedback of the PPI panel in the drafting of the patient information leaflet and consent forms to ensure clarity and comprehensibility.

### Ethics and dissemination

This protocol was written in accordance with the Standard Protocol Items: Recommendations for Interventional Trials (SPIRIT) 2013 Statement (see online supplemental file 7—SPIRIT Checklist). The Study Coordination Centre has obtained approval from the London-Riverside Research Ethics Committee (REC) and Health Regulatory Authority (HRA). The study must also receive confirmation of capacity and capability from each participating NHS Trust before accepting participants into the study or any research activity is carried out. The study has also been submitted to research ethics committees in all participating countries, and each centre must confirm local ethical and hospital approval before starting recruitment. The study will be conducted in accordance with the recommendations for physicians involved in research on human subjects adopted by the 18th World Medical Assembly, Declaration of Helsinki 1964 and later revisions. Publication will be in line with National Institutes of Health Research (NIHR) Open Access policy.

10.1136/bmjopen-2021-055543.supp7 Supplementary data

## Supplementary Material

Reviewer comments

Author's manuscript
