# Supplementary figures and images for: Perinatal and 2-year neurodevelopmental outcome in late preterm fetal compromise: the TRUFFLE 2 randomised trial protocol
Source: BMJ Open. 2022 Apr 15;12(4):e055543. doi: 10.1136/bmjopen-2021-055543 (PMC9014041; doi:10.1136/bmjopen-2021-055543)

## TRUFFLE 2 flowchart

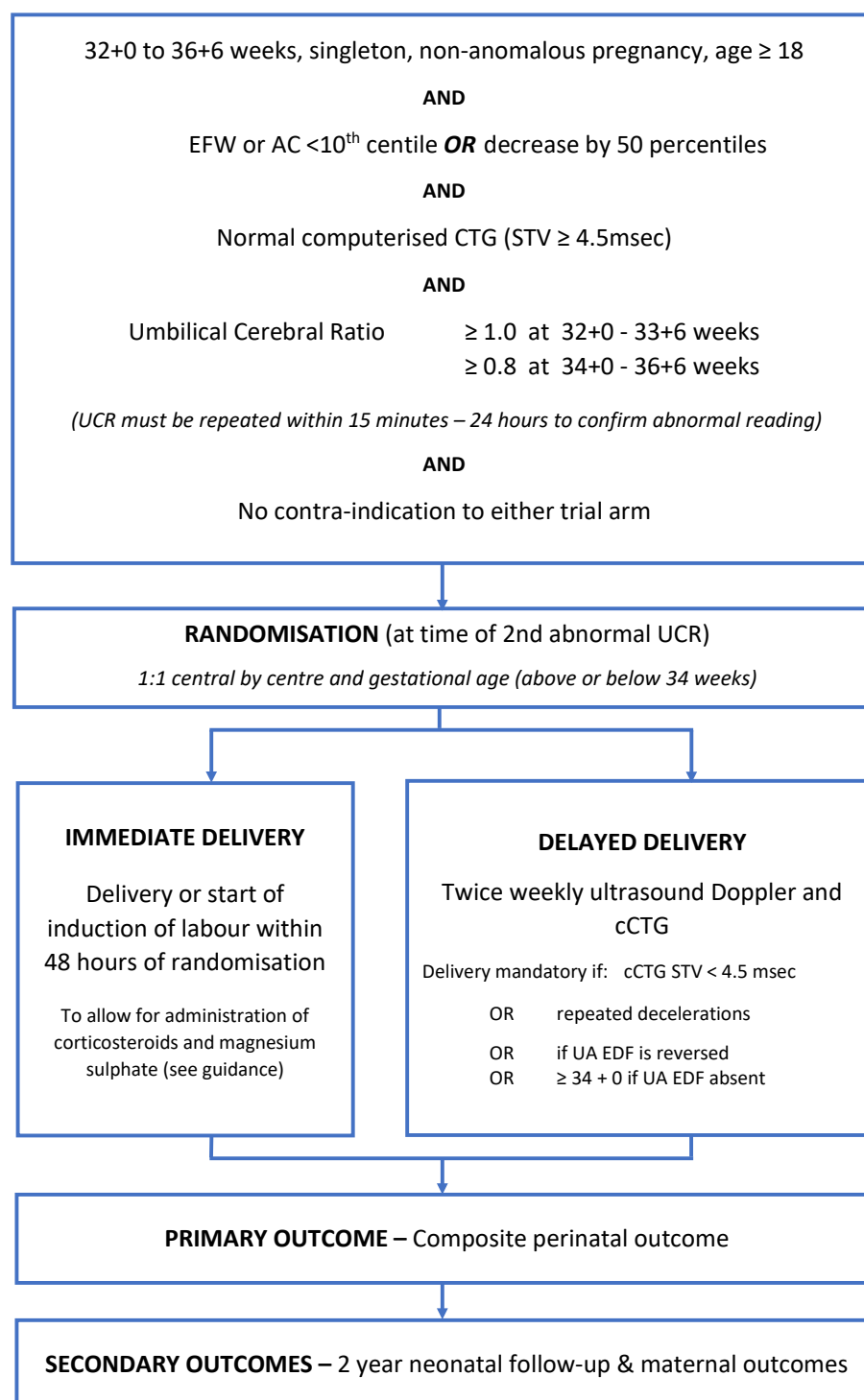

Supplement: Supplementary data [file bmjopen-2021-055543supp001.pdf]
