# Supplementary material for: Perinatal and 2-year neurodevelopmental outcome in late preterm fetal compromise: the TRUFFLE 2 randomised trial protocol
Source: BMJ Open. 2022 Apr 15;12(4):e055543. doi: 10.1136/bmjopen-2021-055543 (PMC9014041; doi:10.1136/bmjopen-2021-055543)
Supplement: Supplementary data [file bmjopen-2021-055543supp002.pdf]

ADD LOCAL HOSPITAL HEADER

Information Sheet for Research Participants
TRUFFLE 2 study

Study title:

Perinatal and 2 year neurodevelopmental outcome in late preterm fetal compromise: the TRUFFLE 2 Randomised Trial

Chief Investigator:

Professor Christoph Lees, MD FRCOG

Principal Local Investigator:

You are being invited to take part in a research study, called the TRial of Umbilical and Fetal FLow in Europe (TRUFFLE) 2 Study. This leaflet is to help you decide whether to participate. It tells you why this study is being conducted and what taking part will mean for you. Please take time to read it carefully. Please get in touch with the study team if anything is not clear or if you would like more information. Take your time to decide whether or not you wish to be involved.

If you decide not to take part your future care will not be affected. If you do take part but decide later on that you don't want to after all, you can withdraw at any time – you do not have to give a reason and your care will not be affected.

Who is organising the research?

The TRUFFLE 2 study is being carried out in this hospital by ………………………………………………………… and their team of doctors and midwives. It is also taking place in centres across the UK and internationally by members of the TRUFFLE research group. This group is made up of researchers and health professionals who specialise in caring for babies and mothers during pregnancy.

This study is sponsored and organised by Imperial College London, with The Centre for Trials Research, Cardiff University managing the trial on a day to day basis. The trial is being funded by the UK National Institute for Health Research (NIHR).

What is the purpose of the study?

Some babies grow more slowly in the womb than expected. This is called 'fetal growth restriction'. The slow growth can be seen on a scan.

Mylrea-Foley B, *et al. BMJ Open* 2022; 12:e055543. doi: 10.1136/bmjopen-2021-055543

Poor growth can be a warning sign about the baby's wellbeing. Doctors have many ways to monitor growth restricted babies, but there is no treatment in the womb; the only treatment is to deliver them. TRUFFLE is investigating the best time for this.

At the end of pregnancy (after 37 weeks) delivery is often recommended as there are fewer risks from birth at that point. Very early in pregnancy (before 32 weeks) doctors usually wait as long as possible, because the risks of premature birth are relatively large.

But when a pregnancy is affected by fetal growth restriction between 32 and 36 weeks of pregnancy, the decision about whether to deliver is more difficult. The possible problems of being delivered early must be balanced against the potential problems for the baby from growing slowly whilst in the womb, including stillbirth.

Currently, doctors don't have good information to help them decide about the best time to deliver a baby between 32 and 36 weeks of pregnancy. At the moment many different approaches are being used. This study aims to find answers about the safest time to deliver the baby.

How will the study work?

Pregnant women whose babies are either smaller or growing more slowly than expected between 32 and 36 weeks of pregnancy will be invited to take part in the screening phase of the study. At this point we will ask your permission to collect information about you and your baby.

Baseline Questionnaire
If you agree to take part in the screening phase of the study, we will ask you for your email address so we can email you a questionnaire to complete. This questionnaire will ask you questions about your ethnicity, job status and drinking and smoking habits.

Apart from this, your care will not change during the screening phase. You will have regular scans and baby heart rate monitoring as normal for small babies. One of the scan tests involves measuring the blood flow through the umbilical cord, and another the blood flow to the baby's brain.

If the blood flow is redirected to the baby's brain, this may be an early warning sign of problems and can be measured by a Doppler ultrasound. The idea is that if the placenta is not working well the baby responds by diverting blood to the most vital organ, the brain. However, many babies have this redistribution pattern remain otherwise perfectly healthy. We can test this by looking at the heart rate pattern. This normally varies moment by moment. You can see this as the line on the monitor being wiggly, rather than flat. The heart rate monitor also calculates a number to measure this pattern objectively. This number is known as short term variation.

If your baby's blood flow is redirected to their brain, but they also have a normal heart rate pattern, some experts would recommend delivery. Others would prefer to wait for the heart rate pattern to change. If you were not taking part in the TRUFFLE 2 trial your treatment would depend on which hospital and/or expert was treating you.

At this point we will invite you to join the main TRUFFLE 2 trial.
If you agree to participate at this stage you will be randomly allocated by a computer to receive one of two methods of treatment.
The two groups are:

- To begin delivery within two days of the Doppler ultrasound blood flow test showing blood flow being redirected to the baby's brain, but before the heart rate pattern changes.
- To wait to deliver until the baby's heart rate pattern changes. This will typically involve twice weekly appointments for baby heart rate monitoring. Your doctor will recommend the exact frequency of this monitoring.

The way in which you give birth, vaginal or caesarean, will not be altered by participation in the trial. That will be left up to your preference and your doctor's recommendation. Your doctors may advise drugs that would be given routinely to improve baby's health when they are born early. These might include steroid injections for lung development and magnesium sulphate infusion for brain protection. Again, these will not be altered by participation in the trial. Your doctors will tell you what drug regime is normally used in your hospital.

We will assess how your baby is when she or he is first born, and then check their development up to two years of age by emailing you questionnaires to complete.

General Health Questionnaire
A general health questionnaire may be emailed to you 6, 12, 18 and 24 months after your baby is born. This questionnaire will ask you details of any hospital admissions for your baby in the last 6 months.

PARCA-R Questionnaire
The PARCA-R questionnaire may be emailed to you 2 years after your baby is born. This questionnaire is made up of two parts to look at your child's development. The questionnaire will be completed by you and will take 15 minutes.

All these questionnaires will be emailed to the email address you provided to the TRUFFLE 2 trials team. The email will come from no-reply@CastorEDC.com, please make sure you check your inbox around the times described above and let the research midwife know if you change your email address. Once completed the questionnaires will be sent back to the TRUFFLE 2 trials team.

We will also use anonymous information collected in the study to determine if there is a difference in the healthcare costs from the different groups in the TRUFFLE 2 study.

Do I have to take part?

No. It is your choice. If you are willing to be part of the study, you will be asked to complete a consent form. If you prefer not to take part, tell your doctor and we will not ask you again. If you decide to take part you are still free to withdraw at any time and without giving a reason. This will not affect the standard of care you receive in the future.

What are the possible disadvantages and risks of taking part?

The timing of your baby's birth will be based on which treatment group you are allocated to. The risks of being born too early are mainly of breathing problems for the baby and a very small risk of bleeding in the brain related to prematurity. The risks of waiting are that the baby's condition may deteriorate rapidly such that he or she gets seriously short of oxygen. All of these risks are very small. Neither ultrasound nor baby heart rate monitoring will cause harm to your baby directly.

What are the possible benefits of taking part?

There are no direct benefits to you from taking part in this study. There is some evidence that people who participate in medical research studies have generally better outcomes than those who don't. We hope that many women and their children in the future will benefit from your participation and the information we gain from this study.

What if a problem is detected?

If any unexpected problems are detected over the course of the study, your doctor will treat you as they think best, whatever trial group you are in. If, for example, you were allocated to the "wait for fetal heart rate changes before birth" group and a new problem, such as bleeding behind the placenta, developed such that delivery became the safest option, your doctor would deliver you. In such a scenario we would still wish to follow up you and your baby.

What happens if I withdraw?

If you decide to withdraw from the study, there are two options.

The first option is it withdraw consent to follow the treatment you were randomised to. This request will be respected and you can still give permission to be followed up by the study team and your data still analysed. You can decide to withdraw from the timing of delivery part of the study at any time without explanation. If you do so, your future care will not be affected by your decision.

The second option is to withdraw from the randomised treatment but also from any follow up data being collected as well. You are free to withdraw from follow up, however if many participants withdraw from follow up it could impact our results, we would therefore strongly prefer that you do not do this. If follow up becomes difficult, please discuss this with the study doctors or midwives. We will usually be able to reduce the burden, perhaps by limiting our contact to your GP rather than contacting you directly. Data that has been collected with your permission before you withdraw from follow up will be included in the study analysis.

Will my taking part in this study be kept confidential?

Imperial College London is the sponsor for this study based in the United Kingdom. We will be using information from you and your medical records in order to undertake this study and will act as the data controller for this study. This means that we are responsible for looking after your information and using it properly. Imperial College London will keep identifiable information about you for 10 years.

Further information on Imperial College London's retention periods may be found at https://www.imperial.ac.uk/media/imperial-college/administration-and-support-services/records-and-archives/public/RetentionSchedule.pdf.

Your rights to access, change or move your information are limited, as we need to manage your information in specific ways in order for the research to be reliable and accurate. If you withdraw from the study, we will keep the information about you that we have already obtained. To safeguard your rights, we will use the minimum personally-identifiable information possible. You can find out more about how we use your information [Dr Christoph Lees Email contact: c.lees@imperial.ac.uk].

LEGAL BASIS

Mylrea-Foley B, *et al. BMJ Open* 2022; 12:e055543. doi: 10.1136/bmjopen-2021-055543

As a university we use personally-identifiable information to conduct research to improve health, care and services. As a publicly-funded organisation, we have to ensure that it is in the public interest when we use personally-identifiable information from people who have agreed to take part in research. This means that when you agree to take part in a research study, we will use your data in the ways needed to conduct and analyse the research study.

Health and care research should serve the public interest, which means that we have to demonstrate that our research serves the interests of society as a whole. We do this by following the UK Policy Framework for Health and Social Care Research.

INTERNATIONAL TRANSFERS

There may be a requirement to transfer information to countries outside the European Economic Area (for example, to a research partner). Where this information contains your personal data, Imperial College London will ensure that it is transferred in accordance with data protection legislation. If the data is transferred to a country which is not subject to a European Commission (EC) adequacy decision in respect of its data protection standards, Imperial College London will enter into a data sharing agreement with the recipient organisation that incorporates EC approved standard contractual clauses that safeguard how your personal data is processed.

CONTACT US

If you wish to raise a complaint on how we have handled your personal data or if you want to find out more about how we use your information, please contact Imperial College London's Data Protection Officer via email at dpo@imperial.ac.uk, via telephone on 020 7594 3502 and via post at Imperial College London, Data Protection Officer, Faculty Building Level 4, London SW7 2AZ.

If you are not satisfied with our response or believe we are processing your personal data in a way that is not lawful you can complain to the Information Commissioner's Office (ICO). The ICO does recommend that you seek to resolve matters with the data controller (us) first before involving the regulator.

Imperial College London will collect information about you for this research study from Imperial College Healthcare Trust. This information will include your hospital number, contact details and health information, which is regarded as a special category of information. We will use this information to conduct this study and contact you with questionnaires.

How we will use and store your information

The TRUFFLE-2 researchers will use and store information about you and your baby for the purpose of the research. This will include contacting you, your hospital doctors and your family doctor to follow you both up. They will keep your information secure and confidential in accord with European data protection rules. Certain authorised individuals may also look at your medical and research records to check the accuracy of the research study.

Potential use of study data for future research

When you agree to take part anonymous information about your health and care may be provided to other authorised researchers. This use of your information without your explicit permission is strictly regulated and will not identify you.

Mylrea-Foley B, *et al. BMJ Open* 2022; 12:e055543. doi: 10.1136/bmjopen-2021-055543

We will also ask your permission to contact you in future to participate in future research studies where your identifiable information would be used. Your participation in such research will only be with your consent.

Who has reviewed the study?

This study was given a favourable ethical opinion for conduct in the NHS by the **London- Riverside** Regional Ethics Committee NHS Health Research Authority.

The IRAS reference number is 266400.

What if something goes wrong?

Imperial College London holds insurance policies which apply to this study. If you experience harm or injury as a result of taking part in this study, you will be eligible to claim compensation without having to prove that Imperial College is at fault. This does not affect your legal rights to seek compensation. If you are harmed due to someone's negligence, then you may have grounds for a legal action.

Regardless of this, if you wish to complain, or have any concerns about any aspect of the way you have been treated during the course of this study then you should immediately inform the Investigator (Dr Christoph Lees Email contact: c.lees@imperial.ac.uk).

The normal National Health Service complaints mechanisms are also available to you. If you are still not satisfied with the response, you may contact the Imperial AHSC Joint Research Compliance Office.

Contact for further information:

For more information you can phone:

Research Fellow and Midwife on 02033137316, or Professor Christoph Lees on 02075942104

Or write to either:

TRUFFLE 2 Investigators, Centre for Fetal Care, Queen Charlotte's & Chelsea Hospital, Du Cane Road, W12 0HS, London
Email: Imperial.TRUFFLEstudy@nhs.net

PALS:

If you have any concerns or wish to complain the details of your local Patient Advice and Liaison Service (PALS) are available on your local hospital's website or on www.nhs.uk.

ADD LOCAL HOSPITAL HEADER

INFORMED CONSENT FORM for DATA COLLECTION

Full Title of Project: **Perinatal and 2 year neurodevelopmental outcome in late preterm fetal compromise: the TRUFFLE 2 Randomised Trial**

Chief Investigator: **Professor Christoph Lees**

Participant number: ☐☐ __ ☐☐☐ __ ☐☐☐

This consent form is for patients who are PRE-ELIGIBLE, who have a small baby with a NORMAL cerebral Doppler.

	**Please initial box**
1. I confirm that I have read and understand the patient information sheet dated ……/……../………., version ……. for the above trial. I have had the opportunity to ask questions and have had these answered satisfactorily.	☐
2. I understand that my participation is voluntary and I am free to withdraw at any time, without giving any reason and without my medical care or legal rights being affected.	☐
3. I understand that sections of my and my baby's medical notes may be looked at by responsible individuals from Imperial College London, from the NHS Trust or from regulatory authorities where it is relevant to my taking part in this research.	☐
4. I give permission for these individuals to access my and my baby's records that are relevant to this research.	☐
5. **Optional:** I give consent for information collected about me and my baby to be used to support other ethically approved research in the future, including those outside of the EEA.	☐
6. **Optional:** I consent to being contacted to potentially taking part in other research studies.	☐
7. I consent for information from my records to be used as part of the TRUFFLE 2 trial.	☐
8. I understand that this form is NOT consent for randomisation.	☐

© Imperial College London

Mylrea-Foley B, *et al. BMJ Open* 2022; 12:e055543. doi: 10.1136/bmjopen-2021-055543

______________________	______________	______________
Name of Subject	Signature	Date
______________________	______________	______________
Name of Person taking consent	Signature	Date
______________________	______________	______________
Principal Investigator	Signature	Date

1 copy for subject; 1 copy for Principal Investigator; 1copy to be kept with hospital notes

© Imperial College London

Mylrea-Foley B*, et al. BMJ Open* 2022; 12:e055543. doi: 10.1136/bmjopen-2021-055543
