# Supplementary material for: Perinatal and 2-year neurodevelopmental outcome in late preterm fetal compromise: the TRUFFLE 2 randomised trial protocol
Source: BMJ Open. 2022 Apr 15;12(4):e055543. doi: 10.1136/bmjopen-2021-055543 (PMC9014041; doi:10.1136/bmjopen-2021-055543)
Supplement: Supplementary data [file bmjopen-2021-055543supp003.pdf]

ADD LOCAL HOSPITAL HEADER

INFORMED CONSENT FORM for RANDOMISATION

Full Title of Project: **Perinatal and 2 year neurodevelopmental outcome in late preterm fetal compromise: the TRUFFLE 2 Randomised Trial**

Chief Investigator: **Professor Christoph Lees**

Participant number: ☐☐ __ ☐☐☐ __ ☐☐☐

This consent form is for patients who are ELIGIBLE FOR RANDOMISATION. This means they have a small baby (EFW or AC <10th or fallen 50 centiles) AND an abnormal UCR (repeated to confirm abnormality within 2-24 hours).

Please initial box

Part A

1. I confirm that I have read and understand the patient information sheet dated ... / ... /... *Version ....* for the above trial. I have had the opportunity to ask questions and have had these answered satisfactorily. ☐

2. I understand that my participation is voluntary and I am free to withdraw at any time, without giving any reason and without my medical care or legal rights being affected. ☐

3. I understand that sections of any of my and my baby's medical notes may be looked at by responsible individuals from Imperial College London, from the NHS Trust or from regulatory authorities where it is relevant to my taking part in this research. ☐

4. I give permission for these individuals to access my and my baby's records that are relevant to this research. ☐

5. I understand that my family doctor will be informed of my participation in the trial. ☐

6. **Optional:** I give consent for information collected about me and my baby to be used to support other ethically approved research in the future, including those outside of the EEA. ☐

7. **Optional:** I consent to being contacted to potentially taking part in other research studies. ☐

Part B

8. I consent to be randomised to either immediate or delayed delivery for this trial. ☐

________________________	________________	________________
Name of Subject	Signature	Date
________________________	________________	________________
Name of Person taking consent	Signature	Date
________________________	________________	________________
Principal Investigator	Signature	Date

1 copy for subject; 1 copy for Principal Investigator; 1copy to be kept with hospital notes

V6.7 02/09/20

© Imperial College London

IRAS ID:

Imperial College London

Mylrea-Foley B, *et al. BMJ Open* 2022; 12:e055543. doi: 10.1136/bmjopen-2021-055543
