# Supplementary material for: Perinatal and 2-year neurodevelopmental outcome in late preterm fetal compromise: the TRUFFLE 2 randomised trial protocol
Source: BMJ Open. 2022 Apr 15;12(4):e055543. doi: 10.1136/bmjopen-2021-055543 (PMC9014041; doi:10.1136/bmjopen-2021-055543)
Supplement: Supplementary data [file bmjopen-2021-055543supp004.pdf]

Parent Report of Children's Abilities – Revised (PARCA-R Questionnaire)

Your child's health and development at 2 years

In this form we ask you to answer some questions about your child and your family. This information is used to work out how your child is developing now that she/he is just over two years old. **Please complete all the questions as accurately as possible.**

If you need any help completing the questionnaire, or have any queries about the questions, please do not hesitate to ask the doctor about these at your appointment.

Permissions:
The PARCA questionnaire was adapted for use with infants born preterm (Johnson et al., Dev Med Child Neurol 2004, 46;389-397) with permission from Saudino, Dale, Oliver, Petrill, Richardson, Rutter, Simonoff, Stevenson & Plomin (1998). The language measures included in this questionnaire are used with permission from the MacArthur-Bates CDI Advisory Board, Chair: Larry Fenson (2016).

For further information about the English version of the PARCA-R questionnaire, please **email**: **parca-r@leicester.ac.uk**

Your child's play

As a parent, you will have a good idea of what your child can and can't do. Listed below are a number of activities. Please indicate whether or not your child can do the activity. That is, if you have seen your child do the activity (or something similar) then tick the box under "YES". If you know that your child would not be able to do it, then tick the box under "NO". If you are not sure whether or not your child can do it, then tick the box under "DON'T KNOW". Please answer every question.

Please keep in mind that these questions are for children ranging from 18 months to 4 years. Some activities may be easy for your child, others may be difficult. Most children of your child's age will not be able to do some of the activities.

		YES	NO	DON'T KNOW
1	**Does your child copy things you do such as cuddling a teddy?** (Try it out if not sure by cuddling a teddy and then giving it to your child. Say: Now you cuddle teddy)	☐	☐	☐
2	**When you hide a toy in full view of your child, will s/he look for it and find it?** (Try this out by covering a small toy with a cloth or a cup and seeing if s/he uncovers the toy)	☐	☐	☐
3	**Can your child put a simple piece, such as a square or an animal, into the correct place in a puzzle board?**	☐	☐	☐
4	**Some toys have several holes or openings with different shapes, such as a circle, triangle, and star. Could your child put the shapes into the right openings?**	☐	☐	☐
5	**Can your child stack two small blocks or toys on top of each other?**	☐	☐	☐
6	**Can your child put together, by him/herself, a puzzle or something similar where the pieces fit together?**	☐	☐	☐
7	**If so, can s/he do this for a puzzle with ten or more pieces?**	☐	☐	☐
8	**Can your child mark on a piece of paper using the tip of a crayon, pencil, or chalk?**	☐	☐	☐
9	**Can your child draw a more or less straight line on paper?**	☐	☐	☐
10	**Does your child turn, or try to turn, pages of a book one at a time?**	☐	☐	☐
11	**Does your child ever pretend that one object, such as a block, is another object, such as a car or a telephone?**	☐	☐	☐

For further information about the English version of the PARCA-R questionnaire, please **email**:

parca-r@leicester.ac.uk

		YES	NO	DON'T KNOW
12	**Can your child stack three small blocks or toys on top of each other by him/herself?**	☐	☐	☐
13	**Does your child ever pretend to do things? For example, riding a horse or making a cup of tea?**	☐	☐	☐
14	**Can your child push a car along the floor with the wheels on the floor?**	☐	☐	☐
15	**Does your child look with interest at pictures in a book?**	☐	☐	☐
16	**Does your child point to pictures in a book?**	☐	☐	☐
17	**Does your child try to copy things you do, such as stirring with a spoon in a cup?**	☐	☐	☐
18	**Can your child stack seven small blocks or toys on top of each other by him/herself?**	☐	☐	☐
19	**Does your child point or show where people or objects are when you ask: "Where is the light?" "Where is Daddy?" or "Where is Teddy?"**	☐	☐	☐
20	**Does your child ever pretend that two dolls are playing together, or are talking to each other, or one is feeding the other?**	☐	☐	☐
21	**Does your child ever play pretend games with another child, pretending to be someone else, such as a mummy, daddy, policeman, or nurse?**	☐	☐	☐
22	**Does your child ever play any game with another child that involves taking turns?**	☐	☐	☐
23	**Does your child ever copy some action shortly (within a few minutes) after s/he has seen it?**	☐	☐	☐
24	**Can your child fetch something, such as a toy, from another room by him/herself when you ask?**	☐	☐	☐
25	**Does your child know where some things belong, such as, that his/her toys belong in a box?**	☐	☐	☐
26	**Does your child ever save or put to one side a biscuit (or snack) for later, on his/her own?**	☐	☐	☐
27	**Have you ever seen your child get together three or more toys before beginning to play with them?**	☐	☐	☐
28	**Have you ever seen your child sort things (blocks, other toys) into groups or piles that go together on his/her own?**	☐	☐	☐

For further information about the English version of the PARCA-R questionnaire, please **email**:

parca-r@leicester.ac.uk

		YES	NO	DON'T KNOW
29	**If your child wants something out of reach, does s/he go and find a chair or box to stand on?**	☐	☐	☐
30	**When your child uses or plays with a telephone, does s/he speak into the mouthpiece not the earpiece?**	☐	☐	☐
31	**When your child drinks from a cup, is s/he careful about putting it down, trying not to spill it?**	☐	☐	☐
32	**Does your child try to turn doorknobs, twist tops, or screw lids on or off jars?**	☐	☐	☐
33	**Does your child recognise him/her self when looking in the mirror?**	☐	☐	☐
34	**Does your child ever use his/her index (first) finger to point to show an interest in something?**	☐	☐	☐

For further information about the English version of the PARCA-R questionnaire, please **email**:

parca-r@leicester.ac.uk

What your child can say

1. Children understand many more words than they can say. Here, we are only interested in the words your child SAYS. Please tick all the words you have heard your child say. If your child uses a different pronunciation of a word – e.g., "tend" for pretend, or "duce" for juice – tick it anyway. Please keep in mind that this is only a sample of words; your child may know many other words not on this list.

☐ Baa baa	☐ Cream cracker	☐ Bed	☐ Carry	☐ Last
☐ Meow	☐ Juice	☐ Bedroom	☐ Chase	☐ Tiny
☐ Ouch/ow	☐ Meat	☐ Settee/sofa	☐ Pour	☐ Wet
☐ Uh-oh/oh dear	☐ Milk	☐ Oven/cooker	☐ Finish	☐ After
☐ Woof woof	☐ Peas	☐ Stairs	☐ Fit	☐ Day
☐ Bear	☐ Hat	☐ Flag	☐ Hug/cuddle	☐ Tonight
☐ Bird	☐ Necklace	☐ Rain	☐ Listen	☐ Our
☐ Cat	☐ Shoe	☐ Star	☐ Like	☐ Them
☐ Dog	☐ Sock	☐ Swing	☐ Pretend	☐ This
☐ Duck	☐ Chin	☐ School	☐ Rip/tear	☐ Us
☐ Horse	☐ Ear	☐ Sky	☐ Shake	☐ Where
☐ Aeroplane	☐ Hand	☐ Zoo	☐ Taste	☐ Beside
☐ Boat	☐ Leg	☐ Friend	☐ Gentle	☐ Down
☐ Car	☐ Pillow	☐ Mummy/mum	☐ Think	☐ Under
☐ Ball	☐ Comb	☐ Person	☐ Wish	☐ All
☐ Book	☐ Lamp/torch	☐ Bye/byebye	☐ All gone	☐ Much
☐ Game	☐ Plate	☐ Hi/hello	☐ Cold	☐ Could
☐ Sandwich	☐ Rubbish	☐ No	☐ Fast	☐ Need to
☐ Fish	☐ Tray	☐ Shopping	☐ Happy	☐ Would
☐ Sauce	☐ Towel	☐ Thank you	☐ Hot	☐ If

For further information about the English version of the PARCA-R questionnaire, please **email**:
parca-r@leicester.ac.uk

How your child uses words

2. We would like to know how your child uses the words s/he can say. Please tick one box for each question below to tell us whether your child uses words like this often, sometimes, or not yet.

Please keep in mind that these questions are for children up to 4 years of age. Many children of your child's age will not be able say some of the words or sentences below.

		OFTEN	SOMETIMES	NOT YET
**A1**	Does your child ever talk about past events or people who are not present? For example, a child who saw a carnival last week might later say 'carnival', 'clown', or 'band'.	☐	☐	☐
**A2**	Does your child ever talk about something that is going to happen in the future? E.g. saying 'choo-choo' or 'bus' before you leave the house on a trip, or saying 'swing' when you are going to the park?	☐	☐	☐
**A3**	Does your child ever talk about objects that are not present? For example, asking about a missing toy not in the room, or asking about someone not present?	☐	☐	☐
**A4**	Does your child understand if you ask for something that is not in the room? For example, would s/he go to the bedroom to get a teddy bear when you say 'Where's the bear?'	☐	☐	☐
**A5**	Does your child know who things belong to? For example, a child might point to mummy's shoe and say 'Mummy'.	☐	☐	☐
**A6**	Has your child started to put together words yet, such as 'Daddy gone' or 'Doggie bite'?	☐	☐	☐

If you answered "Sometimes" or "Often" to question A6, please answer all the questions on the next page.

For further information about the English version of the PARCA-R questionnaire, please **email**:

parca-r@leicester.ac.uk

Mylrea-Foley B, *et al. BMJ Open* 2022; 12:e055543. doi: 10.1136/bmjopen-2021-055543

3. For EACH PAIR of sentences below – A and B – please tick the one that sounds MOST like the way your child talks at the moment, even if s/he would not say that EXACT sentence. If your child is saying sentences even more complicated than the two examples provided, tick B.

		Talking about something happening right now			Talking about something that already happened			
**A7**			**A8**			**A9**		
A	☐	I make tower	A	☐	Daddy pick me up	A	☐	That my truck
B	☐	I making tower	B	☐	Daddy picked me up	B	☐	That's my truck
**A10**			**A11**			**A12**		
A	☐	Baby crying	A	☐	There a doggie	A	☐	Coffee hot
B	☐	Baby is crying	B	☐	There's a doggie	B	☐	That coffee hot
**A13**			**A14**			**A15**		
A	☐	I no do it	A	☐	I like read stories	A	☐	Biscuit mummy
B	☐	I can't do it	B	☐	I like to read stories	B	☐	Biscuit for mummy
**A16**			**A17**			**A18**		
A	☐	Don't read book	A	☐	Baby want eat	A	☐	Look at me
B	☐	Don't want you read that book	B	☐	Baby want to eat	B	☐	Look at me dancing

Thank you very much for your time

For further information about the English version of the PARCA-R questionnaire, please **email**:

parca-r@leicester.ac.uk

Mylrea-Foley B, *et al. BMJ Open* 2022; 12:e055543. doi: 10.1136/bmjopen-2021-055543
