# Supplementary material for: Perinatal and 2-year neurodevelopmental outcome in late preterm fetal compromise: the TRUFFLE 2 randomised trial protocol
Source: BMJ Open. 2022 Apr 15;12(4):e055543. doi: 10.1136/bmjopen-2021-055543 (PMC9014041; doi:10.1136/bmjopen-2021-055543)
Supplement: Supplementary data [file bmjopen-2021-055543supp005.pdf]

Survey 'GB_InfantHealthSurvey'

GB_InfantHealthSurvey - InfantGrowth

Number	Question	Answers
	**Could you enter below the last weight and height of your child and the date when it was measured. Please use the measurements from your last visit to a child health clinic, or take the measurements yourself.**	
1.1	Date of measurement	(dd-mm-yyyy)
1.2	Height (cm)	cm
1.3	Weight (Kg)	kg

GB_InfantHealthSurvey - InfantInpatientCare

Number	Question	Answers
	**The following questions ask about your child's use of hospital services for the LAST SIX MONTHS only. Please answer all questions as accurately as possible.**	
2.1	Has your child been admitted to hospital in the last six months?	○ Yes ● No
2.1.1	***If 'Has your child been admitted to hospital in the last six months?' is equal to 'Yes' answer this question:***<br>In which month was your child admitted in hospital?	○ January<br>○ February<br>○ March<br>○ April<br>○ May<br>○ June<br>○ July<br>○ August<br>○ September<br>○ October<br>○ November<br>○ December

2.1.2	***If 'Has your child been admitted to hospital in the last six months?' is equal to 'Yes' answer this question:***<br>In which year was your child admitted in hospital?	○ 2020<br>○ 2021<br>○ 2022<br>○ 2023<br>○ 2024<br>○ 2025<br>○ 2026
2.1.3	***If 'Has your child been admitted to hospital in the last six months?' is equal to 'Yes' answer this question:***<br>Was the admission planned or an emergency	○ Planned<br>○ Emergency
2.1.4	***If 'Has your child been admitted to hospital in the last six months?' is equal to 'Yes' answer this question:***<br>What was the reason for the admission?	
2.1.5	***If 'Has your child been admitted to hospital in the last six months?' is equal to 'Yes' answer this question:***<br>Did your child stay overnight in the hospital?	○ Yes ○ No
2.1.5.1	***If 'Did your child stay overnight in the hospital?' is equal to 'Yes' answer this question:***<br>For how many nights did your child stay in hospital?	
2.1.6	***If 'Has your child been admitted to hospital in the last six months?' is equal to 'Yes' answer this question:***<br>Did your child receive intensive care?	○ Yes ○ No
2.1.6.1	***If 'Did your child receive intensive care?' is equal to 'Yes' answer this question:***<br>For how many nights did your child stay in the intensive care?	
2.2	Check Admission	
2.2.1	***If 'Check Admission' is equal to '1' answer this question:***<br>Has your child been admitted to hospital for a second time in the last six months?	○ Yes ○ No
	**--_SECOND ADMISSION_---**	

GB_InfantHealthSurvey - InfantOutpatientCare

Number	Question	Answers
	**The following questions ask if your child attended a hospital outpatient department/clinic for the LAST SIX MONTHS only. Please answer all questions as accurately as possible.**	
3.1	Has your child attended a hospital outpatient department/clinic in the last six months	○ Yes ◉ No
3.1.1	***If 'Has your child attended a hospital outpatient department/clinic in the last six months' is equal to 'Yes' answer this question:***<br>Which type of clinic did your child visit during the last six months?	○ Accident or Emergency<br>○ Paediatric / Neonatal Follow-up<br>○ Orthopaedic<br>○ Audiology / Hearing<br>○ Eye / Vision<br>○ General Medicine<br>○ Dermatology<br>○ Other
3.1.1.1	***If 'Which type of clinic did your child visit during the last six months?' is equal to 'Other' answer this question:***<br>Which specialty of outpatient department / clinic did your child visit during the last six months?	
3.1.2	***If 'Has your child attended a hospital outpatient department/clinic in the last six months' is equal to 'Yes' answer this question:***<br>What was the reason for this visit?	
3.1.3	***If 'Has your child attended a hospital outpatient department/clinic in the last six months' is equal to 'Yes' answer this question:***<br>How many times did your child visit this clinic during the past half year?	
3.2	CheckOPD	
3.2.1	***If 'CheckOPD' is equal to '1' answer this question:***<br>Has your child attended a second hospital outpatient department/clinic in the last six months	○ Yes ○ No
	**---_SECOND VISIT_---**	
