# Supplementary material for: Perinatal and 2-year neurodevelopmental outcome in late preterm fetal compromise: the TRUFFLE 2 randomised trial protocol
Source: BMJ Open. 2022 Apr 15;12(4):e055543. doi: 10.1136/bmjopen-2021-055543 (PMC9014041; doi:10.1136/bmjopen-2021-055543)
Supplement: Supplementary data [file bmjopen-2021-055543supp006.pdf]

TRUFFLE 2

Imperial College London

Doppler Image Scoring

For Umbilical Artery:

PULSEWAVE IMAGE (/6)	1 point	0 point
Magnification	Doppler display occupies 50% or more of the image	Doppler display occupies <50% of the image
Angle of insonation	<30 degrees	30 degrees or more
Sweep speed	Doppler spectrum has 4-10 waveforms	Doppler spectrum has < 4 (3 or less) or > 10 waveforms (11 or more)
Sample gate	Large enough to include 3/4th of the vessel diameter	Smaller than 3/4th of the vessel diameter
Appropriate PRF	The waveform fits at least 75% of the pulse wave Doppler scale or PRF (pulse repetition frequency)	The waveform fits < 75% of the pulse wave Doppler scale or PRF
Image quality	Uniform arterial waveforms, no aliasing or background artefacts or fetal breathing movements	Variable arterial waveforms/ aliasing/background artefacts/fetal breathing movements

For Middle Cerebral Arterial: (as above)

PULSEWAVE IMAGE (/6)	1 point	0 point
Magnification	Doppler display occupies 50% or more of the image	Doppler display occupies <50% of the image
Angle of insonation	<30 degrees	30 degrees or more
Sweep speed	Doppler spectrum has 4-10 waveforms	Doppler spectrum has < 4 (3 or less) or > 10 waveforms (11 or more)
Sample gate	Large enough to include 3/4th of the vessel diameter	Smaller than 3/4th of the vessel diameter
Appropriate PRF	The waveform fits at least 75% of the pulse wave Doppler scale or PRF	The waveform fits < 75% the pulse wave Doppler scale or PRF
Image quality	Uniform arterial waveforms, no aliasing or background artefacts or fetal breathing movements	Variable arterial waveforms/ aliasing/background artefacts/fetal breathing movements
