# Supplementary material for: Perinatal and 2-year neurodevelopmental outcome in late preterm fetal compromise: the TRUFFLE 2 randomised trial protocol
Source: BMJ Open. 2022 Apr 15;12(4):e055543. doi: 10.1136/bmjopen-2021-055543 (PMC9014041; doi:10.1136/bmjopen-2021-055543)
Supplement: Author's manuscript [file bmjopen-2021-055543.draft_revisions.pdf]

BMJ Open

BMJ Open is committed to open peer review. As part of this commitment we make the peer review history of every article we publish publicly available.

When an article is published we post the peer reviewers' comments and the authors' responses online. We also post the versions of the paper that were used during peer review. These are the versions that the peer review comments apply to.

The versions of the paper that follow are the versions that were submitted during the peer review process. They are not the versions of record or the final published versions. They should not be cited or distributed as the published version of this manuscript.

BMJ Open is an open access journal and the full, final, typeset and author-corrected version of record of the manuscript is available on our site with no access controls, subscription charges or pay-per-view fees (http://bmjopen.bmj.com).

If you have any questions on BMJ Open's open peer review process please email info.bmjopen@bmj.com

BMJ Open

Perinatal and 2 year neurodevelopmental outcome in late preterm fetal compromise: the TRUFFLE 2 Randomised Trial

Journal:	*BMJ Open*
Manuscript ID	bmjopen-2021-055543
Article Type:	Protocol
Date Submitted by the Author:	19-Jul-2021

Complete List of Authors:	Mylrea-Foley, Bronacha; Imperial College London, Obstetrics and Gynaecology, Queen Charlotte's & Chelsea Hospital<br>Arabin, Birgit; Humboldt University of Berlin, Department of Obstetrics Charite<br>Berger, Astrid; Medical University of Innsbruck, Department of Gynecology and Obstetrics<br>bergman, Eva; Uppsala University, Department of Women's and Children's Health<br>Bhide, Amarnath; St George's University Hospitals NHS Foundation Trust, Fetal Medicine Unit<br>Bilardo, Caterina; Vrije Universiteit Amsterdam, Department of Obstetrics<br>Breeze, Andrew; Leeds Teaching Hospitals NHS Trust, Fetal medicine Unit<br>Brodszki, Jana; Skanes universitetssjukhus Lund, Department of Obstetrics and Gynecology<br>Calda, Pavel; Charles University, Department of Obstetrics and Gynaecology<br>Cannings-John, Rebecca; Cardiff University Centre for Trials Research,<br>Cesari, Elena; Ospedale dei Bambini Vittore Buzzi, Department of Obstetrics and Gynaecology<br>Cetin, I; Vittore Buzzi Children's Hospital, Department of Obstetrics and Gynecology, University of Milan<br>Dall'Asta, Andrea; University of Parma, Medicine and Surgery; Imperial College London Faculty of Medicine, Surgery and Cancer<br>Ebbing, Cathrine; Universitetet i Bergen,<br>Ferrazzi, Enrico; Fondazione IRCCS Ca' Granda Ospedale Maggiore Policlinico, Clinical Sciences Medical School<br>Frusca, Tiziana; University of Parma, Department of Obstetrics and Gynaecology<br>Goodier, Jenny; Imperial College London, Obstetrics and Gynaecology, Queen Charlotte & Chelsea Hospital<br>Gyselaers, Wilfried; Ziekenhuis Oost-Limburg, Department of Obstetrics and Gynecology; Hasselt University,<br>Hecher, Kurt; University Medical Center Hamburg-Eppendorf, Department of Obstetrics and Fetal Medicine<br>Klaritsch, Philipp; Medical University of Graz, Division of Obstetrics and Maternal Fetal Medicine<br>Krofta, Ladislav; Institute for Care of Mother and Child<br>Lindgren, Peter; Karolinska Institutet, Center for fetal medicine<br>Lobmaier, Silvia; Technical University of Munich, Department of

For peer review only - http://bmjopen.bmj.com/site/about/guidelines.xhtml

Obstetrics and Gynecology, Klinikum rechts der Isar
Marlow, Neil; University College London
Marsal, K; Lunds Universitet, Obstetrics and Gynaecology, Faculty of Medicine
Maruotti, Giuseppe; Federico II University Hospital, Department of Neurosciences, Reproductive and Dentistry Sciences
Mecacci, Federico; University Hospital Careggi, High Risk Pregnancy Unit
Mullins, E; Imperial College London, metabolism, digestion and reproduction; Imperial College
Myklestad, Kirsti ; St Olavs Hospital University Hospital in Trondheim, Department of Obstetrics, Children`s and Women`s Health
Napolitano, Raffaele; University College London, UCL Elizabeth Garrett Anderson Institute for Women's Health
Potter, Claire; Cardiff University, Centre for Trials Research
Prefumo, Federico; Università degli Studi di Brescia, Department of Obstetrics and Gynaecology
Raio, Luigi; University of Bern, Department of Obstetrics and Gynaecology
Richter, Jute; Katholieke Universiteit Leuven UZ Leuven, Department of Gynecology and Obstetrics
Sande, Ragnar; Stavanger University Hospital, Department of Obstetrics and Gynaecology
Stampalija, Tamara ; IRCCS materno infantile Burlo Garofolo Dipartimento di Pediatria, Unit of Fetal Medicine and Prenatal Diagnosis
Thornton, Jim; University of Nottingham, Department of Obstetrics and Gynaecology, City Hospital
Townson, Julia; Cardiff University, Centre for Trials Research
Valensise, Herbert; Policlinico Casilino, Division of Obstetrics and Gynaecology
Visser, Gerard ; Utrecht University, Department of Obstetrics, University Medical Center
Wee, Ling; Princess Alexandra Hospital NHS Trust, Obstetrics And Gynaecology
Wolf, Hans; Amsterdam University Medical Centres, Department of Obstetrics and Gynaecology
Lees, Christoph; Imperial College London, Obstetrics and Gynaecology, Queen Charlotte's and Chelsea Hospital

Keywords: Fetal medicine < OBSTETRICS, Maternal medicine < OBSTETRICS, Ultrasonography < OBSTETRICS

SCHOLARONE™
Manuscripts

I, the Submitting Author has the right to grant and does grant on behalf of all authors of the Work (as defined in the below author licence), an exclusive licence and/or a non-exclusive licence for contributions from authors who are: i) UK Crown employees; ii) where BMJ has agreed a CC-BY licence shall apply, and/or iii) in accordance with the terms applicable for US Federal Government officers or employees acting as part of their official duties; on a worldwide, perpetual, irrevocable, royalty-free basis to BMJ Publishing Group Ltd ("BMJ") its licensees and where the relevant Journal is co-owned by BMJ to the co-owners of the Journal, to publish the Work in this journal and any other BMJ products and to exploit all rights, as set out in our licence.

The Submitting Author accepts and understands that any supply made under these terms is made by BMJ to the Submitting Author unless you are acting as an employee on behalf of your employer or a postgraduate student of an affiliated institution which is paying any applicable article publishing charge ("APC") for Open Access articles. Where the Submitting Author wishes to make the Work available on an Open Access basis (and intends to pay the relevant APC), the terms of reuse of such Open Access shall be governed by a Creative Commons licence – details of these licences and which Creative Commons licence will apply to this Work are set out in our licence referred to above.

Other than as permitted in any relevant BMJ Author's Self Archiving Policies, I confirm this Work has not been accepted for publication elsewhere, is not being considered for publication elsewhere and does not duplicate material already published. I confirm all authors consent to publication of this Work and authorise the granting of this licence.

For peer review only

Title:

Perinatal and 2 year neurodevelopmental outcome in late preterm fetal compromise: the TRUFFLE 2 Randomised Trial

Authors

Bronacha Mylrea-Foley[1], Birgit Arabin[2], Astrid Berger[3], Eva Bergman[4], Amarnath Bhide[5], Caterina M Bilardo[6], Andrew Breeze[7], Jana Brodszki[8], Pavel Calda[9], Rebecca Cannings-John[10], Elena Cesari[11], Irene Cetin[11], Andrea Dall'Asta[12], Catherine Ebbing[13], Enrico Ferrazzi[14], Tiziana Frusca[12], Jenny Goodier[1], Wilfred Gyselaers[15], Kurt Hecher[16], Philipp Klaritsch[17], Ladislav Krofta[18], Peter Lindgren[19], Silvia M Lobmaier[20], Neil Marlow[21], Karel Marsal[22], Giuseppe M Maruotti[23], Federico Mecacci[24], Edward Mullins[1], Kirsti Myklestad[25], Raffaele Napolitano[21], Claire Potter[10], Federico Prefumo[26], Luigi Raio[27], Jute Richter[28], Ragnar Kvie Sande[29], Tamara Stampalija[30], Jim G Thornton[31], Julia Townson[10], Herbert Valensise[32], Gerard HA Visser[33], Ling Wee[34], Hans Wolf[35], Christoph Lees[1]* and on behalf of the **TRUFFLE 2 investigators Group**

*Corresponding author

Prof Christoph Lees, c.lees@imperial.ac.uk

Imperial College Healthcare NHS Trust
Centre for Fetal Care, Queen Charlotte's & Chelsea Hospital
Du Cane Road
London
W12 0HS

1 Imperial College London, Obstetrics and Gynaecology, Queen Charlotte's & Chelsea Hospital London, London, UK
2 Department of Obstetrics Charite, Humboldt University of Berlin, Berlin, Germany
3. Department of Gynecology and Obstetrics, Medical University of Innsbruck, Innsbruck, Austria
4. Department of Women's and Children's Health, Uppsala University, Uppsala, Sweden
5. Fetal Medicine Unit, St George's University Hospitals NHS Foundation Trust, London, UK
6. Department of Obstetrics Amsterdam, Vrije Universiteit Amsterdam, Noord-Holland, Netherlands
7. Fetal medicine Unit, Leeds Teaching Hospitals NHS Trust, Leeds, UK
8. Department of Obstetrics and Gynecology, Lund Skanes universitetssjukhus Lund, , Skåne, Sweden
9. Department of Obstetrics and Gynaecology, Charles University, Praha, Czech Republic
10. Centre for Trials Research, Cardiff University, Cardiff, UK
11. Department of Obstetrics and Gynecology, Vittore Buzzi Hospital, University of Milan, Italy
12. Medicine and Surgery, University of Parma, Parma, Italy
13. Universitetet i Bergen, Bergen, Norway
14. Department of Clinical Sciences and Community Health, Fondazione IRCCS Ca' Granda Ospedale Maggiore Policlinico, Italy
15. Department of Obstetrics and Gynecology, Hospital Oost-Limburg, Genk, Belgium
16. Department of Obstetrics and Fetal Medicine, University Medical Center Hamburg-Eppendorf, Hamburg, Germany

For peer review only - http://bmjopen.bmj.com/site/about/guidelines.xhtml

17. Division of Obstetrics and Maternal Fetal Medicine, Medical University of Graz, Graz, Austria
18. Institute for Care of Mother and Child, Prague, Czech Republic
19 Division of Obstetrics and Gynecology, Department of Clinical Science, Intervention & Technology (CLINTEC), Karolinska Institutet, Stockholm, Sweden
20. Department of Obstetrics and Gynecology, Klinikum rechts der Isar, Technical University of Munich, Germany
21. Elizabeth Garrett Anderson Institute for Women's Health University College London, London, UK
22. Obstetrics and Gynaecology, Faculty of Medicine, Lunds Universitet, Lund, Sweden
23. Department of Neurosciences, Reproductive and Dentistry Sciences, Federico II University Hospital, Napoli, Italy
24. High Risk Pregnancy Unit, University Hospital Careggi, Firenze, Italy
25. Department of Obstetrics, Children`s and Women`s Health, St Olavs Hospital University Hospital, Trondheim, Norway
26. Department of Obstetrics and Gynaecology, Università degli Studi di Brescia, Brescia, Italy
27. Department of Obstetrics and Gynaecology, University of Bern, Bern, Switzerland
28. Department of Gynecology and Obstetrics, Katholieke Universiteit Leuven, Leuven, Belgium
29. Department of Obstetrics and Gynaecology, Stavanger University Hospital, Stavanger, Norway
30. Unit of Fetal Medicine and Prenatal Diagnosis, RCCS materno infantile Burlo Garofolo Dipartimento di Pediatria, Trieste, Italy
31. Department of Obstetrics and Gynaecology, University of Nottingham, City hospital, Nottingham, UK
32. Division of Obstetrics and Gynaecology Policlinico Casilino, Roma, Italy
33. Department of Obstetrics, University Medical Center, Utrecht University, Utrecht, Netherlands
34. Obstetrics And Gynaecology, Princess Alexandra Hospital NHS Trust, UK
35. Department of Obstetrics and Gynaecology, Amsterdam University Medical Centres, Amsterdam, Netherlands

Word Count:

3988

For peer review only

For peer review only - http://bmjopen.bmj.com/site/about/guidelines.xhtml

TRUFFLE 2 Investigator Group List:

Ajlana Mulic-Lutvica, Alexey Kholin, Alice Zavatta, Andrea Smith, Andrej Černý, Andrew Breeze, Andrew Sharp, Andy Simm, Angela Ramoni, Angelo Sirico, Anke Diemert, Antonio Lanzone, Aris Papageorghiou, Barry Lloyd, Bianca Masturzo, Christina Ammari, Christine Morfeld, Christopher Lloyd, Claudia Seidig, Constantin Von Keisenberg, Dagmar Pilz, Damiano Lopresti, Daniele Farsetti, Danielle Thornton, Danilo Buca, Dietmar Schlembach, Ekkehard Schleußner, Elena Mantovani, Emanuela Taricco, Emma Bertucci, Enrico Ferrazzi, Erich Cosmi, Eva Ostermayer, Ferenc Macsali, Francesca Ferrari , Francesca Presti, Francesco D'Antonio, Francesco Picciotto, Gareth Waring, Georgios Rembouskos, Giuseppe Cali, Giuseppe Rizzo, Grazia Maria Tiralongo, Ilaria Fantasia, Ilaria Giuditta Ramezzana, Ilenia Mappa, Ioannis Kyvernitakis, Iris Derwig, Iris Dressler-Steinbach, Jiří Vojěch, Jørgen Linde, Julia Binder, Karen Melchiorre, Kate Walker, Kinga Chalubinski, Kristiina Rull, Kristina Kernell, Lars Brodowski, Laura Sarno, Ligita Jokibkiene, Liina Rajasalu, Lina-Ana Fryszer, Louisa Jones, Magdalena Bednarek-Jędrzejek, Makrina Savvidou, Maria Stefopoulou, Marianna Rambaldi, Mark Kilby, Nia Jones, Nicola Fratelli, Nishigandh Deole, Patrick Greimel, Petra Pateisky, Pilar Palmrich, Ralf Schild, Roland Devlieger, Sabina Ondrová, Sarah Gumpert, Sasha Taylor, Saskia Schmidt, Sebastian Kwiatkowski, Serena Caterina, Serena Ottanelli, Serena Simeone, Sergio Ferrazzani, Silvia Salvi, Silvia Visentin, Sofia Amylidi-Mohr, Spyros Bakalis, Stefan Verlohren, Susanne Dargel, Sven Kehl, Tanja Groten, Tatjana Radaelli, Tinne Mesens, Tiziana Fanelli, Torbjørn Eggebø, Tullio Ghi, Tuuli Haabpiht, Wassim Hassan, Yvonne Heiman, Zulfiya Khodzhaeva

For peer review only

For peer review only - http://bmjopen.bmj.com/site/about/guidelines.xhtml

Abstract:

Introduction

Following the detection of FGR, there is no consensus about the criteria that should trigger elective delivery in the late preterm period. The consequences of inappropriate early or late delivery are potentially important yet practice varies widely around the world, with abnormal findings from fetal heart rate monitoring invariably leading to delivery. Indices derived from fetal cerebral Doppler examination may guide such decisions although there are few studies in this area. We propose a randomised, controlled trial to establish the optimum method of timing delivery between 32 weeks and 36 weeks 6 days of gestation. We hypothesise that delivery on evidence of cerebral blood flow redistribution reduces a composite of perinatal poor outcome, death and short-term hypoxia-related morbidity, with no worsening of neurodevelopmental outcome at 2 years.

Methods and Analysis

Women with non-anomalous singleton pregnancies 32+0 to 36+6 weeks of gestation in whom the estimated fetal weight or abdominal circumference is <10th percentile or has decreased by 50 percentiles since 18-32 weeks will be included for observational data collection. Participants will be randomised if cerebral blood flow redistribution is identified, based on umbilical to middle cerebral artery pulsatility index ratio (UCR) values. Computerised cardiotocography (cCTG) must show normal fetal heart rate variability (STV ≥4.5msec) and absence of decelerations at randomisation. Randomisation will be 1:1 to immediate delivery or delayed delivery (based on cCTG abnormalities or other worsening fetal condition). The primary outcome is poor condition at birth and/or fetal or neonatal death and/or major neonatal morbidity, the secondary non-inferiority outcome is two-year infant general health and neurodevelopmental outcome based on the PARCA-R questionnaire.

Ethics and Dissemination

The Study Coordination Centre has obtained approval from the London – Riverside Research Ethics Committee (REC) and Health Regulatory Authority (HRA). Publication will be in line with NIHR Open Access policy.

Registration details;

IRAS Project ID: 266400

REC reference: 20/LO/0031

ISRCTN registry: 76016200

For peer review only - http://bmjopen.bmj.com/site/about/guidelines.xhtml

Strengths and limitations of this study:

An Article Summary, placed after the abstract, consisting of the heading 'Strengths and limitations of this study', and containing up to five short bullet points, no longer than one sentence each, that relate specifically to the methods.

- Changes in cerebral Doppler measures are considered a strong candidate for triggering delivery following detection of late preterm fetal growth restriction, though there are few data to support this approach.
- The primary outcome is a composite of peri- and neonatal outcome, and infants will be followed to determine health and two-neurodevelopment at 2 years to ensure safety and non-inferiority of intervention.
- This appropriately powered UK and European multicentre trial will inform NHS policy and the findings will be generalizable, substantially advancing understanding as to how to manage late preterm fetal growth restriction.

For peer review only

Introduction:

Third trimester poor fetal growth and compromise are strongly associated with stillbirth, neonatal illness[1] and an increased risk of fetal or neonatal brain injury[2]. The only therapeutic option is delivery of the fetus. This poses a dilemma: delivery too early may incur the complications of prematurity, delivery too late risks further fetal compromise, brain injury and late stillbirth. The problem of when to deliver these babies is twofold: there is no consensus on how to identify fetal compromise and there is no 'ideal' evidence-based monitoring strategy. Current screening strategies include standardised symphysis-fundal height measurement, third trimester ultrasound and umbilical Doppler velocimetry[3,4]. NICE acknowledges that methods to identify fetal growth restriction (FGR) are 'poorly developed or not tested by rigorous methodology'.

The use of Doppler ultrasound of fetal vessels allows non-invasive assessment of blood flow. Fetal 'cerebral blood flow redistribution', prioritising blood flow toward the brain at the expense of other organs, is a response to an adverse intrauterine environment characterised by hypoxemia. Ultrasound markers of such fetal compromise include Doppler assessment of blood flow velocity[5–7] and abdominal circumference growth velocity 'drop off' in the third trimester[8,9]. Intervention by delivery of the fetus in response to either evidence of blood flow redistribution or fetal growth slowing has never been tested in a randomised trial. Computerised cardiotocography (cCTG) has been used in several observational studies to determine fetal condition in relation to hypoxaemia and acidosis. The RCOG recommends that research is required to evaluate the effectiveness of third trimester ultrasound assessment, concluding that 'middle cerebral artery Doppler may be a more useful test in small for gestational age fetuses detected after 32 weeks'[4] but does not define parameters that should trigger delivery. Even with effective screening for fetal growth restriction, the questions of how to monitor and when to deliver would remain.

A Cochrane review of the management of 'compromised babies' at term showed no difference in perinatal or long-term outcome with a policy of early delivery versus conservative management[10]. Only three trials were included: two included small babies, both part of the DIGITAT study (a small pilot and the main trial[11]), the third included babies with reduced amniotic fluid. There is no Cochrane review on the optimal timing of delivery in late preterm growth restricted babies.

A systematic review included only one trial of timed delivery in late preterm babies. The Growth Restriction Intervention Trial[12] included 547 babies from 24-36 weeks of gestation with evidence of preterm growth restriction or compromise, where there was clinical uncertainty whether immediate delivery was indicated. Of these, 210 babies were recruited between 33+0 and 36+6 weeks: 107 women were randomised to early delivery and 103 to delayed delivery. Mortality and a range of neurodevelopmental measures were similar between the groups. These results cannot be used to inform management because of the small number of infants assessed using only umbilical artery Doppler velocimetry, the subjective assessment of the cardiotocograph (i.e. not computerised analysis), and the use of clinician's discretion rather than standardised management.

Results from our previous randomised trial of delivery decision-making using Doppler velocimetry and cCTG in pregnancies with FGR at 26-32 weeks of gestation - the TRial of Umbilical Fetal FLow in Europe (TRUFFLE) provided evidence associating monitoring strategies with improved outcomes[13] and guides practice internationally[14,15]. In TRUFFLE (2005-2010) we studied FGR <32 weeks of gestation using cCTG as the standard of care for timing delivery, compared to early or late fetal ductus venosus (DV) Doppler velocimetry changes with a cCTG 'safety net'. Surviving infants whose mothers had been

For peer review only - http://bmjopen.bmj.com/site/about/guidelines.xhtml

randomised to delivery in late ductus venosus changes arm showed better neurodevelopment at 2 years of age[13].

We then carried out the prospective multicentre observational TRUFFLE 2 Feasibility Study (2017-18, n=1024). A range of markers of cerebral redistribution were evaluated as potential delivery trigger points in two gestational age bands. These included the middle cerebral artery (MCA) Doppler and the umbilical cerebral ratio (UCR); calculated by dividing the Doppler pulsatility index of the umbilical artery by that of the MCA. Participating clinicians suggested greater concern about fetal condition is required to trigger delivery at earlier rather than later gestational ages between 32 and 36 weeks. We therefore selected a more abnormal threshold for cerebral redistribution at earlier gestational ages, based on stepped UCR z-score values[16]. Of note was the finding from a prospective study that birth condition, fetal mortality and neonatal morbidity rate were more common among fetuses showing higher UCR z-score, indicating cerebral redistribution, compared to those with a lower UCR z-score (15 vs. 9%)[17].

On this basis, we designed this randomised trial of delivery for women identified with late preterm fetal growth restriction, to test the hypothesis that delivery based on UCR values derived from the feasibility study will safely lead to improved fetal outcomes (TRUFFLE 2).

Methods and analysis:

Study Design

This is an individual unblinded randomised trial of pregnant women experiencing fetal growth restriction. Following informed consent, 1560 women will be randomised to either immediate or delayed delivery. Women are being recruited from UK and European centres.

Potential participants are women with singleton non-anomalous pregnancies between 32+0 and 36+6 weeks of gestation in whom a small for gestational age (SGA) fetus is identified or one whose growth has slowed. This is defined as estimated fetal weight or abdominal circumference <10th percentile and/or having decreased by 50 percentiles since an ultrasound scan at 18-32 weeks. Each centre will utilise local growth charts. Data is collected on absolute measurements and growth chart-derived centiles. Once identified as potential participants women receive regular monitoring as per local standard of care for fetal condition, using ultrasound biometry, umbilical and middle cerebral artery (MCA) Doppler velocimetry assessments and cCTG (using Dawes-Redman criteria). This is recommended to be repeated every 14 days; these observational data are recorded.

Women become eligible for randomisation when signs of fetal cerebral blood flow redistribution are detected by Doppler, defined below and shown in the Study Flowchart (Supplementary File 1 - Flowchart).

Delivery is based on UCR pulsatility index z-scores, of >1.5 (between 32+0 and 33+6 weeks) or >1.0 (34+0 to 36+6 weeks). These correspond to an absolute UCR of ≥1.0 at 32+0 to 33+6 weeks and ≥0.8 at 34+0 to 36+6 weeks. Abnormal UCR measurements must be repeated within 2–24 hours to confirm these values. Randomisation will occur at the time of the second qualifying UCR measurement (this need not be consecutive) and is stratified by centre and gestational age. Randomisation is through the CASTOR website (Amsterdam, NL) into which eligibility, monitoring and outcome data are entered.

For peer review only - http://bmjopen.bmj.com/site/about/guidelines.xhtml

Women are consented in a two-stage process. Stage 1 (pre-eligible) consent is not a prerequisite and women may be consented directly for randomisation with stage 2 (eligible for randomisation) consent.

Pre-eligible (Supplementary File 2 – Consent Form for Observational Data Collection)

1. Consented for prospective data collection once identified as meeting criteria for SGA or slowed fetal growth (as defined above) but not meeting cerebral Doppler thresholds for randomisation. This will include demographics, medical history, ultrasound findings and outcomes. This consent will include obtaining a personal email address, which is entered into and stored on Castor, and willingness to be contacted in the future for follow up where women are randomised.

Eligible for randomisation (Supplementary File 3 – Consent for Randomisation)

2. Consent for randomisation once cerebral redistribution is identified, with UCR (as defined above) of ≥1.0 at 32+0 to 33+6 weeks and ≥0.8 at 34+0 to 36+6 weeks. This consent will also include contact details and willingness to be contacted in the future for follow up data collection.

Women are randomly allocated to either immediate delivery or delayed delivery as defined below. Randomisation has a 1:1 allocation ratio and stratified based on gestational age (above or below 34 weeks) and centre. Randomisation is conducted on the electronic data capture platform (Castor EDC, Amsterdam, The Netherlands).

Immediate Delivery

Participants in the immediate delivery arm will be delivered by Caesarean or induction of labour will be commenced within 48 hours, allowing for administration of corticosteroids and infusion of magnesium sulphate as per local protocol and guidance. Start of induction of labour is defined as administering cervical preparation (cervical balloon, prostaglandins, etc) or artificial rupture of membranes or administration of oxytocin.

Delayed Delivery

Participants in the delayed delivery arm will be monitored using twice weekly Doppler and cCTG monitoring, or more frequently based on local clinical protocols if required. Umbilical artery Doppler velocities may be measured in this time and delivery may be based on these safety net criteria (see box below). We strongly recommend that MCA measurements are not undertaken during monitoring in the delayed delivery arm. Delivery is indicated when short term variation (STV) is <4.5 ms on cCTG or there are repeated fetal heart rate decelerations. Once participants reach 37^{+0} weeks of gestation the delivery plan will be based on local protocols.

Umbilical Doppler delivery thresholds

In all arms absolute indications for delivery include:

- umbilical artery Doppler with reversed end diastolic flow after entry into the trial, OR
- umbilical artery Doppler absent end diastolic flow from 34^{+0} weeks

For peer review only - http://bmjopen.bmj.com/site/about/guidelines.xhtml

Study Timeline

Study set up: 0-10 months; recruitment/randomisation: 11-36 months. Two years follow up questionnaires 35-60 months; analyses, writing up, reporting & dissemination: 61-66 months. This equates to 5 and ½ years (66 months).

Study outcome measures

Primary outcome

The primary outcome for the study is a composite of fetal or infant death, composite measure of poor condition at birth and neonatal morbidity – as defined by presence of any of the following:

1) Poor condition at birth

- Apgar score at 5 minutes <7, umbilical artery pH <7.0 or umbilical vein pH <7.1
- Need for resuscitation with intubation, chest compressions or medication

2) Fetal death/ death before neonatal hospital discharge

3) Neonatal brain injury syndromes

- Infants with a diagnosis consistent with hypoxic ischaemic encephalopathy (HIE): term and near-term infants only
- Infants with a diagnosis of intracranial haemorrhage, perinatal stroke, HIE, central nervous system infection, or kernicterus (bilirubin encephalopathy): all infants
- Preterm white matter disease (periventricular leukomalacia): preterm infants only
- Infants with a recorded seizure confirmed by EEG

4) Respiratory support

- Need for mechanical support of respiration after admission to neonatal unit (NNU), for more than 1 hour; includes need for continuous positive airways pressure (CPAP or NIPPV) or mechanical ventilation via endotracheal tube but excludes need for supplemental oxygen.

5) Cardio-vascular abnormality

- Hypotensive treatment, patent ductus arteriosus requiring treatment, or disseminated coagulopathy;

6) Sepsis

- Clinical sepsis with positive blood culture, or
- necrotising enterocolitis requiring surgery

7) Retinopathy of prematurity requiring treatment (laser or anti-VEGF injections)

Secondary outcomes

For the baby:

1) *Health and developmental outcomes* – assessed using Parent Report of Children's Abilities-Revised (PARCA-R) questionnaire at 2 years corrected age and an Infant Health Questionnaire up to 2 years

For peer review only - http://bmjopen.bmj.com/site/about/guidelines.xhtml

(Supplementary File 4 – PARCA-R Questionnaire and Supplementary File 5 – Infant Health Questionaire).

The PARCA-R [18] will be completed at 24 months term-equivalent age, this allows derivation of the non-verbal cognitive scale and language development scale. Raw scores from the scales are standardised (by corrected age and gender) to a notional population mean of 100 SD=15 and the average of these two component scores will be taken as the overall composite score. Corrected age is used for preterm babies (born before 37 weeks) and represents the age of the child from the estimated date of delivery.

The Infant Health Questionnaire [19] will be used to derive the following health outcomes at 6, 12, 18 and 24 months post-partum:

- Use of any hospital service (yes/no) and total number of contacts over the 2 year period;
- Admitted to hospital (yes/no) and total number of admissions over the 2 year period;
    - Planned/unplanned admissions to hospital (yes/no) over the 2 year period;
    - Intensive care or not over the 2-year period;
- Attended Emergency Department (and not subsequently admitted) (yes/no) over the 2 year period;
- Attended Outpatients/clinic (yes/no) over the 2 year period.

For the mother:

1) Gestational hypertension developed post study entry

- As defined by the International Society for Study of Hypertension in pregnancy (ISSHP): hypertension (blood pressure ≥140/90mmHg) arising de novo after 20 weeks gestation in the absence of proteinuria

2) Pre-eclampsia developed post study entry

- As defined by the ISSHP: blood pressure ≥140/90mmHg AND significant proteinuria (protein/creatinine ratio of 30 mg/mmol or more)

3) Onset of labour (spontaneous, induction (method), prelabour caesarean section)

4) Mode of delivery (spontaneous vaginal, assisted vaginal, caesarean section)

Doppler Quality Control

Sonographer Standardisation: Each sonographer in each centre taking part in the trial are assessed by the local PI. Each sonographer will submit to the local PI two images: pseudo anonymised ultrasound images for each Doppler parameter (umbilical artery and MCA) each showing a colour Doppler image with the gate placed over the vessel, and the pulsed wave Doppler waveform arising from that image. The local PI will determine whether these images are satisfactory using a predefined quality control scoring system (Supplementary File 6 – Doppler Image Scoring Sheet).

Doppler Ultrasound Criteria: Measurements are obtained in fetuses between 32+0 and 36+6 weeks of gestation. Umbilical artery and MCA pulsatility index images are collected according to specific predefined objective criteria for both the colour Doppler images and pulsed wave Doppler.

For peer review only - http://bmjopen.bmj.com/site/about/guidelines.xhtml

Doppler Quality Control: The local PI will provide details of all sonographers having undergone standardisation in that centre to the Centre for Trials Research (CTR). The CTR will independently request all images submitted to the local PI for the first 5 patients randomised from each unit, and then for up to 10% of patients thereafter, for anonymised quality control assessment by the Quality Control Board. All images are collected as pseudo anonymised .jpeg images and saved electronically in a Doppler ultrasound sonographer standardisation file by the PI, with the submitting sonographer identifiable. Images are scored using the predefined scoring criteria. The CTR will manage this process and will provide feedback if necessary, and ensure that members of the TRUFFLE 2 Quality Control Board do not assess images from their own unit.

Participant Entry

Pre-registration evaluations

- Ultrasound scan of fetal growth between 32+0 and 36+6 weeks of gestation, including measurement of MCA and umbilical artery Doppler velocities
- cCTG STV analysed using Dawes-Redman criteria

Inclusion Criteria

(All criteria should be fulfilled to be eligible for randomisation)

- Women ≥ 18 years old
- Pregnant with singleton non-anomalous fetus
- Between 32+0 and 36+6 weeks of gestation
- Estimated fetal weight or abdominal circumference <10th percentile OR decreased by 50 percentiles since an ultrasound scan at 18+0-32+0 weeks and:
- Cerebral redistribution defined as UCR ≥1.0 (between 32+0 and 33+6 weeks) or ≥0.8 (34+0 to 36+6 weeks) **repeated within 2-24 hours**
- Normal STV on cCTG (4.5msec or above)

Exclusion Criteria

- Indication for immediate delivery required within 48 hours
- Unable to give informed consent
- Preterm pre-labour rupture of the membranes
- Suspected placental abruption or antepartum haemorrhage
- Presence of reversed end diastolic flow in the umbilical artery.

Assessment and Follow-up

Assessment of the primary outcome is at infant discharge from the neonatal unit and assessment of the key secondary infant outcomes will use the Infant Health Questionnaire and PARCA-R. The Infant Health Questionnaire will be sent out by investigators via Castor at 6, 12, 18 and 24 months post-partum. Neurodevelopment is assessed at 2 years age corrected for prematurity using the PARCA-R, also sent via Castor (translated and validated into local language). The window for determining 2 year outcome is from 22-28 months over which range the PARCA-R has been standardised.

For peer review only - http://bmjopen.bmj.com/site/about/guidelines.xhtml

Endpoint is 30 months after the estimated date of delivery of the last participant to deliver (24 months follow-up with additional 6 months for data cleaning and additional enquiries).

Statistics and Data Analysis

Sample size

The trial is powered to detect if immediate delivery following cerebral redistribution is superior to expectant management following cerebral redistribution on this outcome. A difference in the proportion with the primary outcome from 15% in the delayed delivery to 9% in the immediate delivery (from TRUFFLE 2 feasibility study) demonstrates an odds ratio of 0.56. At 2-sided 5% significance with 95% power, 780 participants per arm are required, giving 1,560 in total. Given the immediacy of this outcome, no loss to follow-up is expected. An important non-inferiority secondary safety outcome is infant neurodevelopment, which is measured by parent completion questionnaire at 2 years using the PARCA-R, as recommended in NICE Guidance [20], supplemented by infant health information over the intervening 2 years. Assuming a loss to follow-up at 2 years of 20%, 2 year outcomes for approximately 1,248 infants are expected (624 per group assuming no difference in the loss to follow-up between the groups). The PARCA-R questionnaire provides a composite score for neurodevelopment with a standardised mean of 100 and standard deviation of 15. With a one-sided significance level of 1%, under a non-inferiority hypothesis, a sample size of 624 in each group achieves a 98% power to detect a non-inferiority margin of difference in the mean PARCA-R score of no less than 4 points (0.25 of a standard deviation). A margin of no less than 3 points can be detected with 90% power.

Main analysis

The primary analysis approach for the primary outcome of composite of adverse fetal/neonatal outcomes, maternal secondary outcomes and the Infant Health Questionnaire will be intention to treat with participants analysed in the groups to which they are assigned regardless of deviation from the protocol or intervention received. The PARCA-R will be both an intention to treat and a per protocol analysis, since the hypothesis under examination for these outcomes is a non-inferiority hypothesis. The per-protocol analysis will exclude babies of women who do not receive their intervention as planned. As the trial includes multiple centres (and will involve a reasonable number of participants randomised per centre), the analysis will be based on the individual participant, allowing for clustering between participants within centre using robust standard errors. All analyses will additionally adjust for gestational age at inclusion (stratification risk factor used in randomisation) as a fixed factor. For binary outcomes (composite of adverse fetal/neonatal outcomes, gestational hypertension, pre-eclampsia) a logistic regression model will be used to compare this outcome by arm and results presented as odds ratios and two-sided 95% confidence interval (CI). Continuous outcomes (PARCA-R, Infant Health Questionnaire) will be analysed using linear regression and results presented as adjusted differences in means alongside 95% CIs. The Infant Health Questionnaire will also be examined over time using a repeated measures model, and will include an interaction term for time (6, 12, 18 and 24 months) and trial arm to investigate any divergent or convergent pattern in Infant Health Questionnaire. The categorical outcomes of mode of delivery and onset of labour will be compared between trial arms by fitting a multilevel ordinal regression model. The neonatal secondary outcome will be examined using the mean PARCA-R score between each trial arm using linear regression, and a one-sided 95% CI constructed to assess non-inferiority. Additional pre-specified sub-group analyses will be carried out to analyse the primary outcome in the whole cohort by those that are <10th centile (versus ≥10th) and <3rd centile (versus ≥3rd) based on Intergrowth-21[21], WHO[22]

For peer review only - http://bmjopen.bmj.com/site/about/guidelines.xhtml

and customised growth charts, maternal morbidity (yes/no) and corticosteroid administration (yes/no). If numbers are sufficient, we will also describe the intervention effect in the sub-groups <3rd centile versus 4-10th versus >10th centile but will not statistically tested for intervention effect.

All analyses will be undertaken after database lock following data collection at 2 years. No interim analyses are planned. Missing outcome data but will be accounted for in sensitivity analyses using multiple imputation, where we will assume that outcome data are missing at random given the observed measurements. All planned analyses will be described in detail in a Statistical Analysis Plan (SAP), which will be finalised prior to database lock. The reporting of findings will be in accordance with the CONSORT guidelines for RCTs. Statistical analysis will be performed in Stata (version 16 or higher).

Data Collection:

Data collection and randomisation are carried out on a secure cloud-based electronic data capture platform (Castor EDC, Amsterdam, The Netherlands). Participants can only be identified by their recruiting centers using unique trial identifiers. Participant email addresses will be stored for sending follow up questionnaires, this is stored within an encrypted section of Castor only their recruiting center has access to.

Trial monitoring:

A Trial Management Group will meet monthly to review study progress and recruitment targets. In addition there will be a Trial Steering Committee and Data Monitoring Committee, with a majority of independent members, to guarantee the safety of study participants.

A clinical trial risk assessment has been developed by Centre for Trials Research at Cardiff University, to determine the intensity and focus of central and on-site monitoring activity in the TRUFFLE 2 trial. Appropriate monitoring levels will be employed and are fully documented in the trial monitoring plan. Investigators should agree to allow trial related monitoring, including audits and regulatory inspections, by providing direct access to source data/documents as required. Findings generated from on-site and central monitoring will be shared with the Sponsor, CI, PI & local R&D.

Serious Adverse Events (SAE) will be reported for randomised participants and their infants. The reporting period is from the point of randomisation until discharge home for the mother and neonate, maternal death is to be reported up to 42 days after delivery. SAEs will be reported on the data capture platform (Castor) in accordance with the UK policy framework for Health and Social care research.

Patient and Public Involvement:

Patient and Public Involvement (PPI) has been embedded throughout the development of this study.

Sands, the Stillbirth and Neonatal Death charity in the UK, have been closely involved in the development and design of this study. Sands is clear that there is an urgent need for better assessment and care of women and their babies in late pregnancy and are fully supportive of the study. They will play an integral role throughout the study and in publicising the study and disseminating results in accessible formats. The research and development lead at Sands, is a member of the Trial

For peer review only - http://bmjopen.bmj.com/site/about/guidelines.xhtml

Management Group. In their role, they represents many members of the public affected by stillbirth and neonatal death.

An FGR PPI panel was convened to discuss TRUFFLE 2 study design. The purpose of the workshop was to ask members of the public for their views on the study, with specific focus on the concept of randomisation, the current situation for the treatment of FGR, and the scope of this study. The FGR panel comprised eight women, who had experienced FGR, stillbirth or uncomplicated pregnancies. At the workshop the proposed study design was discussed. Following the workshop, the women were asked to complete an anonymous questionnaire relating to early delivery compared to monitoring the health of the baby in the womb and randomisation. Overall, women were supportive of the study, one stated "I believe a lot of women will benefit from this study'. Another stated 'The study will have an impact; it will act as a ripple effect on the family and wider public for the better.' Regarding randomisation, most women said that they would be happy to be randomised to either arm whilst acknowledging that they would need support, one woman stated 'Yes, I would be randomised but would need the right support from the clinical staff. I would need to have 100% trust in my doctors'. Two women from this panel have agreed to be involved in the management of the study as independent members of the Trial Steering Committee. We incorporated the views and feedback of the PPI panel in the drafting of the patient information leaflet and consent forms to ensure clarity and comprehensibility.

For peer review only

Ethics and dissemination:

The Study Coordination Centre has obtained approval from the London - Riverside Research Ethics Committee (REC) and Health Regulatory Authority (HRA). The study must also receive confirmation of capacity and capability from each participating NHS Trust before accepting participants into the study or any research activity is carried out. The study has also been submitted to research ethics committees in all participating countries, and each centre must confirm local ethical and hospital approval before starting recruitment. The study will be conducted in accordance with the recommendations for physicians involved in research on human subjects adopted by the 18th World Medical Assembly, Helsinki 1964 and later revisions. Publication will be in line with NIHR Open Access policy.

Authors' contributions:

BMF wrote the manuscript and collated all comments. CCL reviewed and edited the manuscript. All authors contributed to the development of the protocol in meetings between 2015 and 2019, reviewed the protocol and commented on it.

Funding statement:

This research was funded by the National Institute for Health Research (NIHR) (127976) using UK aid from the UK Government to support global health research. The views expressed in this publication are those of the author(s) and not necessarily those of the NIHR or the UK government. BMF was supported by a Fellowship Grant from Imperial Health Charity (reference: RFRFPrD1920/104).

For peer review only - http://bmjopen.bmj.com/site/about/guidelines.xhtml

Competing interests statement:

All authors have completed the ICMJE uniform disclosure form at www.icmje.org/coi_disclosure.pdf and declare: all authors had financial support from ABC Company for the submitted work; no financial relationships with any organisations that might have an interest in the submitted work in the previous three years; no other relationships or activities that could appear to have influenced the submitted work. (From BMJ website).

For peer review only

For peer review only - http://bmjopen.bmj.com/site/about/guidelines.xhtml

Full references:

1 Baschat AA. Planning management and delivery of the growth-restricted fetus. *Best Pract Res Clin Obstet Gynaecol* 2018;**49**:53–65. doi:10.1016/j.bpobgyn.2018.02.009

2 Malhotra A, Ditchfield M, Fahey MC, *et al.* Detection and assessment of brain injury in the growth-restricted fetus and neonate. *Nat Publ Gr* 2017;**82**:184–93. doi:10.1038/pr.2017.37

3 NICE. Antenatal care for uncomplicated pregnancies | Guidance. 2019.

4 RCOG. Small-for-Gestational-Age Fetus, Investigation and Management (Green-top Guideline No. 31). 2013.

5 Meher S, Hernandez-Andrade E, Basheer SN, *et al.* Impact of cerebral redistribution on neurodevelopmental outcome in small-for-gestational-age or growth-restricted babies: a systematic review. *Ultrasound Obstet Gynecol* 2015;**46**:398–404. doi:10.1002/uog.14818

6 Khalil A, Morales-Rosellõ J, Townsend R, *et al.* Value of third-trimester cerebroplacental ratio and uterine artery Doppler indices as predictors of stillbirth and perinatal loss. *Ultrasound Obstet Gynecol* 2016;**47**:74–80. doi:10.1002/uog.15729

7 Akolekar R, Syngelaki A, Gallo DM, *et al.* Umbilical and fetal middle cerebral artery Doppler at 35-37 weeks' gestation in the prediction of adverse perinatal outcome. *Ultrasound Obstet Gynecol* 2015;**46**:82–92. doi:10.1002/uog.14842

8 Sovio U, White IR, Dacey A, *et al.* Screening for fetal growth restriction with universal third trimester ultrasonography in nulliparous women in the Pregnancy Outcome Prediction (POP) study: A prospective cohort study. *Lancet* 2015;**386**:2089–97. doi:10.1016/S0140-6736(15)00131-2

9 Gordijn SJ, Beune IM, Thilaganathan B, *et al.* Consensus definition of fetal growth restriction: a Delphi procedure. *Ultrasound Obstet Gynecol* 2016;**48**:333–9. doi:10.1002/uog.15884

10 Bond DM, Gordon A, Hyett J, *et al.* Planned early delivery versus expectant management of the term suspected compromised baby for improving outcomes. Cochrane Database Syst. Rev. 2015;**2015**. doi:10.1002/14651858.CD009433.pub2

11 Boers KE, Vijgen SMC, Bijlenga D, *et al.* Induction versus expectant monitoring for intrauterine growth restriction at term: Randomised equivalence trial (DIGITAT). *BMJ* 2011;**342**:35. doi:10.1136/bmj.c7087

12 GRIT study group, Thornton JG, Hornbuckle J, *et al.* Infant wellbeing at 2 years of age in the Growth Restriction Intervention Trial (GRIT): Multicentred randomised controlled trial. *Lancet* 2004;**364**:513–20. doi:10.1016/S0140-6736(04)16809-8

13 Lees CC, Marlow N, Van Wassenaer-Leemhuis A, *et al.* 2 year neurodevelopmental and intermediate perinatal outcomes in infants with very preterm fetal growth restriction (TRUFFLE): A randomised trial. *Lancet* 2015;**385**:2162–72. doi:10.1016/S0140-6736(14)62049-3

14 Martins JG, Biggio JR, Abuhamad A. Society for Maternal-Fetal Medicine Consult Series #52: Diagnosis and management of fetal growth restriction. *Am J Obstet Gynecol* 2020;**223**:B2–17. doi:10.1016/j.ajog.2020.05.010

15 Lees CC, Stampalija T, Baschat A, *et al.* ISUOG Practice Guidelines: diagnosis and management of small-for-gestational-age fetus and fetal growth restriction. *Ultrasound Obstet Gynecol* 2020;**56**:298–312. doi:10.1002/uog.22134

For peer review only - http://bmjopen.bmj.com/site/about/guidelines.xhtml

16 Mylrea-Foley B, Bhide A, Mullins E, *et al.* Building consensus: thresholds for delivery in TRUFFLE-2 randomized intervention study. Ultrasound Obstet. Gynecol. 2020;**56**:285–7. doi:10.1002/uog.22124

17 Stampalija T, Thornton J, Marlow N, *et al.* Fetal cerebral Doppler changes and outcome in late preterm fetal growth restriction: prospective cohort study. *Ultrasound Obstet Gynecol* 2020;:173–81. doi:10.1002/uog.22125

18 Johnson S, Bountziouka V, Brocklehurst P, *et al.* Standardisation of the Parent Report of Children's Abilities–Revised (PARCA-R): a norm-referenced assessment of cognitive and language development at age 2 years. *Lancet Child Adolesc Heal* 2019;**3**:705–12. doi:10.1016/s2352-4642(19)30189-0

19 Chappell LC, Brocklehurst P, Green ME, *et al.* Planned early delivery or expectant management for late preterm pre-eclampsia (PHOENIX): a randomised controlled trial. *Lancet* 2019;**394**:1181–90. doi:10.1016/S0140-6736(19)31963-4

20 NICE. Developmental follow-up of children and young people born preterm | Quality standards. 2018.

21 Papageorghiou AT, Ohuma EO, Altman DG, *et al.* International standards for fetal growth based on serial ultrasound measurements: The Fetal Growth Longitudinal Study of the INTERGROWTH-21st Project. *Lancet* 2014;**384**:869–79. doi:10.1016/S0140-6736(14)61490-2

22 Kiserud T, Piaggio G, Carroli G, *et al. The World Health Organization Fetal Growth Charts : A Multinational Longitudinal Study of Ultrasound Biometric Measurements and Estimated Fetal Weight*. 2017. doi:10.1371/journal.pmed.1002220

Supplementary Files:

1. Supplementary File 1 - Flowchart
2. Supplementary File 2 - Consent for Observational Data Collection
3. Supplementary File 3 - Consent for Randomisation
4. Supplementary File 4 - PARCA-R Questionnaire
5. Supplementary File 5 - Infant Health Questionnaire
6. Supplementary File 6 - Doppler image scoring sheet

For peer review only

TRUFFLE 2 flowchart

32+0 to 36+6 weeks, singleton, non-anomalous pregnancy, age ≥ 18

AND

EFW or AC <10th centile ***OR*** decrease by 50 percentiles

AND

Normal computerised CTG (STV ≥ 4.5msec)

AND

Umbilical Cerebral Ratio > 1.0 at 32+0 - 33+6 weeks
> 0.8 at 34+0 - 36+6 weeks

(UCR must be repeated within 2 – 24 hours to confirm abnormal reading)

AND

No contra-indication to either trial arm

↓

RANDOMISATION (at time of 2nd abnormal UCR)

1:1 central by centre and gestational age (above or below 34 weeks)

↓

IMMEDIATE DELIVERY

Delivery or start of induction of labour within 48 hours of randomisation

To allow for administration of corticosteroids and magnesium sulphate (see guidance)

DELAYED DELIVERY

Twice weekly ultrasound Doppler and cCTG

Delivery mandatory if: cCTG STV < 4.5 msec
OR repeated decelerations
OR if UA EDF is reversed
OR ≥ 34 + 0 if UA EDF absent

↓

PRIMARY OUTCOME – Composite perinatal outcome

↓

SECONDARY OUTCOMES – 2 year neonatal follow-up & maternal outcomes

ADD LOCAL HOSPITAL HEADER

Information Sheet for Research Participants
TRUFFLE 2 study

Study title:

Perinatal and 2 year neurodevelopmental outcome in late preterm fetal compromise: the TRUFFLE 2 Randomised Trial

Chief Investigator:

Professor Christoph Lees, MD FRCOG

Principal Local Investigator:

You are being invited to take part in a research study, called the TRial of Umbilical and Fetal FLow in Europe (TRUFFLE) 2 Study. This leaflet is to help you decide whether to participate. It tells you why this study is being conducted and what taking part will mean for you. Please take time to read it carefully. Please get in touch with the study team if anything is not clear or if you would like more information. Take your time to decide whether or not you wish to be involved.

If you decide not to take part your future care will not be affected. If you do take part but decide later on that you don't want to after all, you can withdraw at any time – you do not have to give a reason and your care will not be affected.

Who is organising the research?

The TRUFFLE 2 study is being carried out in this hospital by …………………………………………………………… and their team of doctors and midwives. It is also taking place in centres across the UK and internationally by members of the TRUFFLE research group. This group is made up of researchers and health professionals who specialise in caring for babies and mothers during pregnancy.

This study is sponsored and organised by Imperial College London, with The Centre for Trials Research, Cardiff University managing the trial on a day to day basis. The trial is being funded by the UK National Institute for Health Research (NIHR).

What is the purpose of the study?

Some babies grow more slowly in the womb than expected. This is called 'fetal growth restriction'. The slow growth can be seen on a scan.

For peer review only - http://bmjopen.bmj.com/site/about/guidelines.xhtml

Poor growth can be a warning sign about the baby's wellbeing. Doctors have many ways to monitor growth restricted babies, but there is no treatment in the womb; the only treatment is to deliver them. TRUFFLE is investigating the best time for this.

At the end of pregnancy (after 37 weeks) delivery is often recommended as there are fewer risks from birth at that point. Very early in pregnancy (before 32 weeks) doctors usually wait as long as possible, because the risks of premature birth are relatively large.

But when a pregnancy is affected by fetal growth restriction between 32 and 36 weeks of pregnancy, the decision about whether to deliver is more difficult. The possible problems of being delivered early must be balanced against the potential problems for the baby from growing slowly whilst in the womb, including stillbirth.

Currently, doctors don't have good information to help them decide about the best time to deliver a baby between 32 and 36 weeks of pregnancy. At the moment many different approaches are being used. This study aims to find answers about the safest time to deliver the baby.

How will the study work?

Pregnant women whose babies are either smaller or growing more slowly than expected between 32 and 36 weeks of pregnancy will be invited to take part in the screening phase of the study. At this point we will ask your permission to collect information about you and your baby.

Baseline Questionnaire

If you agree to take part in the screening phase of the study, we will ask you for your email address so we can email you a questionnaire to complete. This questionnaire will ask you questions about your ethnicity, job status and drinking and smoking habits.

Apart from this, your care will not change during the screening phase. You will have regular scans and baby heart rate monitoring as normal for small babies. One of the scan tests involves measuring the blood flow through the umbilical cord, and another the blood flow to the baby's brain.

If the blood flow is redirected to the baby's brain, this may be an early warning sign of problems and can be measured by a Doppler ultrasound. The idea is that if the placenta is not working well the baby responds by diverting blood to the most vital organ, the brain. However, many babies have this redistribution pattern remain otherwise perfectly healthy. We can test this by looking at the heart rate pattern. This normally varies moment by moment. You can see this as the line on the monitor being wiggly, rather than flat. The heart rate monitor also calculates a number to measure this pattern objectively. This number is known as short term variation.

If your baby's blood flow is redirected to their brain, but they also have a normal heart rate pattern, some experts would recommend delivery. Others would prefer to wait for the heart rate pattern to change. If you were not taking part in the TRUFFLE 2 trial your treatment would depend on which hospital and/or expert was treating you.

At this point we will invite you to join the main TRUFFLE 2 trial.
If you agree to participate at this stage you will be randomly allocated by a computer to receive one of two methods of treatment.
The two groups are:

For peer review only - http://bmjopen.bmj.com/site/about/guidelines.xhtml

- To begin delivery within two days of the Doppler ultrasound blood flow test showing blood flow being redirected to the baby's brain, but before the heart rate pattern changes.
- To wait to deliver until the baby's heart rate pattern changes. This will typically involve twice weekly appointments for baby heart rate monitoring. Your doctor will recommend the exact frequency of this monitoring.

The way in which you give birth, vaginal or caesarean, will not be altered by participation in the trial. That will be left up to your preference and your doctor's recommendation. Your doctors may advise drugs that would be given routinely to improve baby's health when they are born early. These might include steroid injections for lung development and magnesium sulphate infusion for brain protection. Again, these will not be altered by participation in the trial. Your doctors will tell you what drug regime is normally used in your hospital.

We will assess how your baby is when she or he is first born, and then check their development up to two years of age by emailing you questionnaires to complete.

General Health Questionnaire
A general health questionnaire may be emailed to you 6, 12, 18 and 24 months after your baby is born. This questionnaire will ask you details of any hospital admissions for your baby in the last 6 months.

PARCA-R Questionnaire
The PARCA-R questionnaire may be emailed to you 2 years after your baby is born. This questionnaire is made up of two parts to look at your child's development. The questionnaire will be completed by you and will take 15 minutes.

All these questionnaires will be emailed to the email address you provided to the TRUFFLE 2 trials team. The email will come from no-reply@CastorEDC.com, please make sure you check your inbox around the times described above and let the research midwife know if you change your email address. Once completed the questionnaires will be sent back to the TRUFFLE 2 trials team.

We will also use anonymous information collected in the study to determine if there is a difference in the healthcare costs from the different groups in the TRUFFLE 2 study.

Do I have to take part?

No. It is your choice. If you are willing to be part of the study, you will be asked to complete a consent form. If you prefer not to take part, tell your doctor and we will not ask you again. If you decide to take part you are still free to withdraw at any time and without giving a reason. This will not affect the standard of care you receive in the future.

What are the possible disadvantages and risks of taking part?

The timing of your baby's birth will be based on which treatment group you are allocated to. The risks of being born too early are mainly of breathing problems for the baby and a very small risk of bleeding in the brain related to prematurity. The risks of waiting are that the baby's condition may deteriorate rapidly such that he or she gets seriously short of oxygen. All of these risks are very small. Neither ultrasound nor baby heart rate monitoring will cause harm to your baby directly.

What are the possible benefits of taking part?

For peer review only - http://bmjopen.bmj.com/site/about/guidelines.xhtml

There are no direct benefits to you from taking part in this study. There is some evidence that people who participate in medical research studies have generally better outcomes than those who don't. We hope that many women and their children in the future will benefit from your participation and the information we gain from this study.

What if a problem is detected?

If any unexpected problems are detected over the course of the study, your doctor will treat you as they think best, whatever trial group you are in. If, for example, you were allocated to the "wait for fetal heart rate changes before birth" group and a new problem, such as bleeding behind the placenta, developed such that delivery became the safest option, your doctor would deliver you. In such a scenario we would still wish to follow up you and your baby.

What happens if I withdraw?

If you decide to withdraw from the study, there are two options.

The first option is it withdraw consent to follow the treatment you were randomised to. This request will be respected and you can still give permission to be followed up by the study team and your data still analysed. You can decide to withdraw from the timing of delivery part of the study at any time without explanation. If you do so, your future care will not be affected by your decision.

The second option is to withdraw from the randomised treatment but also from any follow up data being collected as well. You are free to withdraw from follow up, however if many participants withdraw from follow up it could impact our results, we would therefore strongly prefer that you do not do this. If follow up becomes difficult, please discuss this with the study doctors or midwives. We will usually be able to reduce the burden, perhaps by limiting our contact to your GP rather than contacting you directly. Data that has been collected with your permission before you withdraw from follow up will be included in the study analysis.

Will my taking part in this study be kept confidential?

Imperial College London is the sponsor for this study based in the United Kingdom. We will be using information from you and your medical records in order to undertake this study and will act as the data controller for this study. This means that we are responsible for looking after your information and using it properly. Imperial College London will keep identifiable information about you for 10 years.

Further information on Imperial College London's retention periods may be found at https://www.imperial.ac.uk/media/imperial-college/administration-and-support-services/records-and-archives/public/RetentionSchedule.pdf.

Your rights to access, change or move your information are limited, as we need to manage your information in specific ways in order for the research to be reliable and accurate. If you withdraw from the study, we will keep the information about you that we have already obtained. To safeguard your rights, we will use the minimum personally-identifiable information possible. You can find out more about how we use your information [Dr Christoph Lees Email contact: c.lees@imperial.ac.uk].

LEGAL BASIS

For peer review only - http://bmjopen.bmj.com/site/about/guidelines.xhtml

As a university we use personally-identifiable information to conduct research to improve health, care and services. As a publicly-funded organisation, we have to ensure that it is in the public interest when we use personally-identifiable information from people who have agreed to take part in research. This means that when you agree to take part in a research study, we will use your data in the ways needed to conduct and analyse the research study.

Health and care research should serve the public interest, which means that we have to demonstrate that our research serves the interests of society as a whole. We do this by following the UK Policy Framework for Health and Social Care Research.

INTERNATIONAL TRANSFERS

There may be a requirement to transfer information to countries outside the European Economic Area (for example, to a research partner). Where this information contains your personal data, Imperial College London will ensure that it is transferred in accordance with data protection legislation. If the data is transferred to a country which is not subject to a European Commission (EC) adequacy decision in respect of its data protection standards, Imperial College London will enter into a data sharing agreement with the recipient organisation that incorporates EC approved standard contractual clauses that safeguard how your personal data is processed.

CONTACT US

If you wish to raise a complaint on how we have handled your personal data or if you want to find out more about how we use your information, please contact Imperial College London's Data Protection Officer via email at dpo@imperial.ac.uk, via telephone on 020 7594 3502 and via post at Imperial College London, Data Protection Officer, Faculty Building Level 4, London SW7 2AZ.

If you are not satisfied with our response or believe we are processing your personal data in a way that is not lawful you can complain to the Information Commissioner's Office (ICO). The ICO does recommend that you seek to resolve matters with the data controller (us) first before involving the regulator.

Imperial College London will collect information about you for this research study from Imperial College Healthcare Trust. This information will include your hospital number, contact details and health information, which is regarded as a special category of information. We will use this information to conduct this study and contact you with questionnaires.

How we will use and store your information

The TRUFFLE-2 researchers will use and store information about you and your baby for the purpose of the research. This will include contacting you, your hospital doctors and your family doctor to follow you both up. They will keep your information secure and confidential in accord with European data protection rules. Certain authorised individuals may also look at your medical and research records to check the accuracy of the research study.

Potential use of study data for future research

When you agree to take part anonymous information about your health and care may be provided to other authorised researchers. This use of your information without your explicit permission is strictly regulated and will not identify you.

For peer review only - http://bmjopen.bmj.com/site/about/guidelines.xhtml

We will also ask your permission to contact you in future to participate in future research studies where your identifiable information would be used. Your participation in such research will only be with your consent.

Who has reviewed the study?

This study was given a favourable ethical opinion for conduct in the NHS by the **London- Riverside** Regional Ethics Committee NHS Health Research Authority.

The IRAS reference number is 266400.

What if something goes wrong?

Imperial College London holds insurance policies which apply to this study. If you experience harm or injury as a result of taking part in this study, you will be eligible to claim compensation without having to prove that Imperial College is at fault. This does not affect your legal rights to seek compensation. If you are harmed due to someone's negligence, then you may have grounds for a legal action.

Regardless of this, if you wish to complain, or have any concerns about any aspect of the way you have been treated during the course of this study then you should immediately inform the Investigator (Dr Christoph Lees Email contact: c.lees@imperial.ac.uk).

The normal National Health Service complaints mechanisms are also available to you. If you are still not satisfied with the response, you may contact the Imperial AHSC Joint Research Compliance Office.

Contact for further information:

For more information you can phone:

Research Fellow and Midwife on 02033137316, or Professor Christoph Lees on 02075942104

Or write to either:

TRUFFLE 2 Investigators, Centre for Fetal Care, Queen Charlotte's & Chelsea Hospital, Du Cane Road, W12 0HS, London
Email: Imperial.TRUFFLEstudy@nhs.net

PALS:

If you have any concerns or wish to complain the details of your local Patient Advice and Liaison Service (PALS) are available on your local hospital's website or on www.nhs.uk.

For peer review only

For peer review only - http://bmjopen.bmj.com/site/about/guidelines.xhtml

ADD LOCAL HOSPITAL HEADER

INFORMED CONSENT FORM for DATA COLLECTION

Full Title of Project: **Perinatal and 2 year neurodevelopmental outcome in late preterm fetal compromise: the TRUFFLE 2 Randomised Trial**

Chief Investigator: **Professor Christoph Lees**

Participant number: ☐☐ __ ☐☐☐ __ ☐☐☐

This consent form is for patients who are PRE-ELIGIBLE, who have a small baby with a NORMAL cerebral Doppler.

	Please initial box
1. I confirm that I have read and understand the patient information sheet dated ……/……../………., version ……. for the above trial. I have had the opportunity to ask questions and have had these answered satisfactorily.	☐
2. I understand that my participation is voluntary and I am free to withdraw at any time, without giving any reason and without my medical care or legal rights being affected.	☐
3. I understand that sections of my and my baby's medical notes may be looked at by responsible individuals from Imperial College London, from the NHS Trust or from regulatory authorities where it is relevant to my taking part in this research.	☐
4. I give permission for these individuals to access my and my baby's records that are relevant to this research.	☐
5. **Optional:** I give consent for information collected about me and my baby to be used to support other ethically approved research in the future, including those outside of the EEA.	☐
6. **Optional:** I consent to being contacted to potentially taking part in other research studies.	☐
7. I consent for information from my records to be used as part of the TRUFFLE 2 trial.	☐
8. I understand that this form is NOT consent for randomisation.	☐

© Imperial College London

For peer review only - http://bmjopen.bmj.com/site/about/guidelines.xhtml

______________________	______________	______________
Name of Subject	Signature	Date
______________________	______________	______________
Name of Person taking consent	Signature	Date
______________________	______________	______________
Principal Investigator	Signature	Date

For peer review only

1 copy for subject; 1 copy for Principal Investigator; 1copy to be kept with hospital notes

© Imperial College London

For peer review only - http://bmjopen.bmj.com/site/about/guidelines.xhtml

ADD LOCAL HOSPITAL HEADER

INFORMED CONSENT FORM for RANDOMISATION

Full Title of Project: **Perinatal and 2 year neurodevelopmental outcome in late preterm fetal compromise: the TRUFFLE 2 Randomised Trial**

Chief Investigator: **Professor Christoph Lees**

Participant number: ☐☐ __ ☐☐☐ __ ☐☐☐

This consent form is for patients who are ELIGIBLE FOR RANDOMISATION. This means they have a small baby (EFW or AC <10th or fallen 50 centiles) AND an abnormal UCR (repeated to confirm abnormality within 2-24 hours).

Please initial box

Part A

1. I confirm that I have read and understand the patient information sheet dated ... / ... /... *Version* .... for the above trial. I have had the opportunity to ask questions and have had these answered satisfactorily. ☐

2. I understand that my participation is voluntary and I am free to withdraw at any time, without giving any reason and without my medical care or legal rights being affected. ☐

3. I understand that sections of any of my and my baby's medical notes may be looked at by responsible individuals from Imperial College London, from the NHS Trust or from regulatory authorities where it is relevant to my taking part in this research. ☐

4. I give permission for these individuals to access my and my baby's records that are relevant to this research. ☐

5. I understand that my family doctor will be informed of my participation in the trial. ☐

6. **Optional:** I give consent for information collected about me and my baby to be used to support other ethically approved research in the future, including those outside of the EEA. ☐

7. **Optional:** I consent to being contacted to potentially taking part in other research studies. ☐

Part B

8. I consent to be randomised to either immediate or delayed delivery for this trial. ☐

Name of Subject	Signature	Date
Name of Person taking consent	Signature	Date
Principal Investigator	Signature	Date

1 copy for subject; 1 copy for Principal Investigator; 1copy to be kept with hospital notes

For peer review only - http://bmjopen.bmj.com/site/about/guidelines.xhtml

© Imperial College London
IRAS ID:
Imperial College London

Parent Report of Children's Abilities – Revised

(PARCA-R Questionnaire)

For peer review only

Your child's health and development at 2 years

In this form we ask you to answer some questions about your child and your family. This information is used to work out how your child is developing now that she/he is just over two years old. **Please complete all the questions as accurately as possible.**

If you need any help completing the questionnaire, or have any queries about the questions, please do not hesitate to ask the doctor about these at your appointment.

Permissions:
The PARCA questionnaire was adapted for use with infants born preterm (Johnson et al., Dev Med Child Neurol 2004, 46;389-397) with permission from Saudino, Dale, Oliver, Petrill, Richardson, Rutter, Simonoff, Stevenson & Plomin (1998). The language measures included in this questionnaire are used with permission from the MacArthur-Bates CDI Advisory Board, Chair: Larry Fenson (2016).

For further information about the English version of the PARCA-R questionnaire, please **email**:
parca-r@leicester.ac.uk

Your child's play

As a parent, you will have a good idea of what your child can and can't do. Listed below are a number of activities. Please indicate whether or not your child can do the activity. That is, if you have seen your child do the activity (or something similar) then tick the box under "YES". If you know that your child would not be able to do it, then tick the box under "NO". If you are not sure whether or not your child can do it, then tick the box under "DON'T KNOW". Please answer every question.

Please keep in mind that these questions are for children ranging from 18 months to 4 years. Some activities may be easy for your child, others may be difficult. Most children of your child's age will not be able to do some of the activities.

		YES	NO	DON'T KNOW
1	**Does your child copy things you do such as cuddling a teddy?** (Try it out if not sure by cuddling a teddy and then giving it to your child. Say: Now you cuddle teddy)	☐	☐	☐
2	**When you hide a toy in full view of your child, will s/he look for it and find it?** (Try this out by covering a small toy with a cloth or a cup and seeing if s/he uncovers the toy)	☐	☐	☐
3	**Can your child put a simple piece, such as a square or an animal, into the correct place in a puzzle board?**	☐	☐	☐
4	**Some toys have several holes or openings with different shapes, such as a circle, triangle, and star. Could your child put the shapes into the right openings?**	☐	☐	☐
5	**Can your child stack two small blocks or toys on top of each other?**	☐	☐	☐
6	**Can your child put together, by him/herself, a puzzle or something similar where the pieces fit together?**	☐	☐	☐
7	**If so, can s/he do this for a puzzle with ten or more pieces?**	☐	☐	☐
8	**Can your child mark on a piece of paper using the tip of a crayon, pencil, or chalk?**	☐	☐	☐
9	**Can your child draw a more or less straight line on paper?**	☐	☐	☐
10	**Does your child turn, or try to turn, pages of a book one at a time?**	☐	☐	☐
11	**Does your child ever pretend that one object, such as a block, is another object, such as a car or a telephone?**	☐	☐	☐

For further information about the English version of the PARCA-R questionnaire, please **email**:

parca-r@leicester.ac.uk

For peer review only - http://bmjopen.bmj.com/site/about/guidelines.xhtml

		YES	NO	DON'T KNOW
12	**Can your child stack three small blocks or toys on top of each other by him/herself?**	☐	☐	☐
13	**Does your child ever pretend to do things? For example, riding a horse or making a cup of tea?**	☐	☐	☐
14	**Can your child push a car along the floor with the wheels on the floor?**	☐	☐	☐
15	**Does your child look with interest at pictures in a book?**	☐	☐	☐
16	**Does your child point to pictures in a book?**	☐	☐	☐
17	**Does your child try to copy things you do, such as stirring with a spoon in a cup?**	☐	☐	☐
18	**Can your child stack seven small blocks or toys on top of each other by him/herself?**	☐	☐	☐
19	**Does your child point or show where people or objects are when you ask: "Where is the light?" "Where is Daddy?" or "Where is Teddy?"**	☐	☐	☐
20	**Does your child ever pretend that two dolls are playing together, or are talking to each other, or one is feeding the other?**	☐	☐	☐
21	**Does your child ever play pretend games with another child, pretending to be someone else, such as a mummy, daddy, policeman, or nurse?**	☐	☐	☐
22	**Does your child ever play any game with another child that involves taking turns?**	☐	☐	☐
23	**Does your child ever copy some action shortly (within a few minutes) after s/he has seen it?**	☐	☐	☐
24	**Can your child fetch something, such as a toy, from another room by him/herself when you ask?**	☐	☐	☐
25	**Does your child know where some things belong, such as, that his/her toys belong in a box?**	☐	☐	☐
26	**Does your child ever save or put to one side a biscuit (or snack) for later, on his/her own?**	☐	☐	☐
27	**Have you ever seen your child get together three or more toys before beginning to play with them?**	☐	☐	☐
28	**Have you ever seen your child sort things (blocks, other toys) into groups or piles that go together on his/her own?**	☐	☐	☐

For further information about the English version of the PARCA-R questionnaire, please **email**:

parca-r@leicester.ac.uk

For peer review only - http://bmjopen.bmj.com/site/about/guidelines.xhtml

		YES	NO	DON'T KNOW
29	**If your child wants something out of reach, does s/he go and find a chair or box to stand on?**	☐	☐	☐
30	**When your child uses or plays with a telephone, does s/he speak into the mouthpiece not the earpiece?**	☐	☐	☐
31	**When your child drinks from a cup, is s/he careful about putting it down, trying not to spill it?**	☐	☐	☐
32	**Does your child try to turn doorknobs, twist tops, or screw lids on or off jars?**	☐	☐	☐
33	**Does your child recognise him/her self when looking in the mirror?**	☐	☐	☐
34	**Does your child ever use his/her index (first) finger to point to show an interest in something?**	☐	☐	☐

For peer review only

For further information about the English version of the PARCA-R questionnaire, please **email**:

parca-r@leicester.ac.uk

What your child can say

1. Children understand many more words than they can say. Here, we are only interested in the words your child SAYS. Please tick all the words you have heard your child say. If your child uses a different pronunciation of a word – e.g., "tend" for pretend, or "duce" for juice – tick it anyway. Please keep in mind that this is only a sample of words; your child may know many other words not on this list.

☐ Baa baa	☐ Cream cracker	☐ Bed	☐ Carry	☐ Last
☐ Meow	☐ Juice	☐ Bedroom	☐ Chase	☐ Tiny
☐ Ouch/ow	☐ Meat	☐ Settee/sofa	☐ Pour	☐ Wet
☐ Uh-oh/oh dear	☐ Milk	☐ Oven/cooker	☐ Finish	☐ After
☐ Woof woof	☐ Peas	☐ Stairs	☐ Fit	☐ Day
☐ Bear	☐ Hat	☐ Flag	☐ Hug/cuddle	☐ Tonight
☐ Bird	☐ Necklace	☐ Rain	☐ Listen	☐ Our
☐ Cat	☐ Shoe	☐ Star	☐ Like	☐ Them
☐ Dog	☐ Sock	☐ Swing	☐ Pretend	☐ This
☐ Duck	☐ Chin	☐ School	☐ Rip/tear	☐ Us
☐ Horse	☐ Ear	☐ Sky	☐ Shake	☐ Where
☐ Aeroplane	☐ Hand	☐ Zoo	☐ Taste	☐ Beside
☐ Boat	☐ Leg	☐ Friend	☐ Gentle	☐ Down
☐ Car	☐ Pillow	☐ Mummy/mum	☐ Think	☐ Under
☐ Ball	☐ Comb	☐ Person	☐ Wish	☐ All
☐ Book	☐ Lamp/torch	☐ Bye/byebye	☐ All gone	☐ Much
☐ Game	☐ Plate	☐ Hi/hello	☐ Cold	☐ Could
☐ Sandwich	☐ Rubbish	☐ No	☐ Fast	☐ Need to
☐ Fish	☐ Tray	☐ Shopping	☐ Happy	☐ Would
☐ Sauce	☐ Towel	☐ Thank you	☐ Hot	☐ If

For further information about the English version of the PARCA-R questionnaire, please **email**:
parca-r@leicester.ac.uk

For peer review only
For peer review only - http://bmjopen.bmj.com/site/about/guidelines.xhtml

How your child uses words

2. We would like to know how your child uses the words s/he can say. Please tick one box for each question below to tell us whether your child uses words like this often, sometimes, or not yet.

Please keep in mind that these questions are for children up to 4 years of age. Many children of your child's age will not be able say some of the words or sentences below.

		OFTEN	SOMETIMES	NOT YET
**A1**	Does your child ever talk about past events or people who are not present? For example, a child who saw a carnival last week might later say 'carnival', 'clown', or 'band'.	☐	☐	☐
**A2**	Does your child ever talk about something that is going to happen in the future? E.g. saying 'choo-choo' or 'bus' before you leave the house on a trip, or saying 'swing' when you are going to the park?	☐	☐	☐
**A3**	Does your child ever talk about objects that are not present? For example, asking about a missing toy not in the room, or asking about someone not present?	☐	☐	☐
**A4**	Does your child understand if you ask for something that is not in the room? For example, would s/he go to the bedroom to get a teddy bear when you say 'Where's the bear?'	☐	☐	☐
**A5**	Does your child know who things belong to? For example, a child might point to mummy's shoe and say 'Mummy'.	☐	☐	☐
**A6**	Has your child started to put together words yet, such as 'Daddy gone' or 'Doggie bite'?	☐	☐	☐

If you answered "Sometimes" or "Often" to question A6, please answer all the questions on the next page.

For further information about the English version of the PARCA-R questionnaire, please **email**:

parca-r@leicester.ac.uk

For peer review only - http://bmjopen.bmj.com/site/about/guidelines.xhtml

3. For EACH PAIR of sentences below – A and B – please tick the one that sounds MOST like the way your child talks at the moment, even if s/he would not say that EXACT sentence. If your child is saying sentences even more complicated than the two examples provided, tick B.

Talking about something happening right now			Talking about something that already happened					
**A7**			**A8**			**A9**		
A	☐	I make tower	A	☐	Daddy pick me up	A	☐	That my truck
B	☐	I making tower	B	☐	Daddy picked me up	B	☐	That's my truck
**A10**			**A11**			**A12**		
A	☐	Baby crying	A	☐	There a doggie	A	☐	Coffee hot
B	☐	Baby is crying	B	☐	There's a doggie	B	☐	That coffee hot
**A13**			**A14**			**A15**		
A	☐	I no do it	A	☐	I like read stories	A	☐	Biscuit mummy
B	☐	I can't do it	B	☐	I like to read stories	B	☐	Biscuit for mummy
**A16**			**A17**			**A18**		
A	☐	Don't read book	A	☐	Baby want eat	A	☐	Look at me
B	☐	Don't want you read that book	B	☐	Baby want to eat	B	☐	Look at me dancing

For peer review only

Thank you very much for your time

For further information about the English version of the PARCA-R questionnaire, please **email**:

parca-r@leicester.ac.uk

For peer review only - http://bmjopen.bmj.com/site/about/guidelines.xhtml

Survey 'GB_InfantHealthSurvey'

GB_InfantHealthSurvey - InfantGrowth

Number	Question	Answers
	**Could you enter below the last weight and height of your child and the date when it was measured. Please use the measurements from your last visit to a child health clinic, or take the measurements yourself.**	
1.1	Date of measurement	*(dd-mm-yyyy)*
1.2	Height (cm)	cm
1.3	Weight (Kg)	kg

GB_InfantHealthSurvey - InfantInpatientCare

Number	Question	Answers
	**The following questions ask about your child's use of hospital services for the LAST SIX MONTHS only. Please answer all questions as accurately as possible.**	
2.1	Has your child been admitted to hospital in the last six months?	○ Yes ◉ No
2.1.1	***If 'Has your child been admitted to hospital in the last six months?' is equal to 'Yes' answer this question:***<br>In which month was your child admitted in hospital?	○ January<br>○ February<br>○ March<br>○ April<br>○ May<br>○ June<br>○ July<br>○ August<br>○ September<br>○ October<br>○ November<br>○ December

2.1.2	***If 'Has your child been admitted to hospital in the last six months?' is equal to 'Yes' answer this question:***<br>In which year was your child admitted in hospital?	○ 2020<br>○ 2021<br>○ 2022<br>○ 2023<br>○ 2024<br>○ 2025<br>○ 2026
2.1.3	***If 'Has your child been admitted to hospital in the last six months?' is equal to 'Yes' answer this question:***<br>Was the admission planned or an emergency	○ Planned<br>○ Emergency
2.1.4	***If 'Has your child been admitted to hospital in the last six months?' is equal to 'Yes' answer this question:***<br>What was the reason for the admission?	
2.1.5	***If 'Has your child been admitted to hospital in the last six months?' is equal to 'Yes' answer this question:***<br>Did your child stay overnight in the hospital?	○ Yes ○ No
2.1.5.1	***If 'Did your child stay overnight in the hospital?' is equal to 'Yes' answer this question:***<br>For how many nights did your child stay in hospital?	
2.1.6	***If 'Has your child been admitted to hospital in the last six months?' is equal to 'Yes' answer this question:***<br>Did your child receive intensive care?	○ Yes ○ No
2.1.6.1	***If 'Did your child receive intensive care?' is equal to 'Yes' answer this question:***<br>For how many nights did your child stay in the intensive care?	
2.2	Check Admission	
2.2.1	***If 'Check Admission' is equal to '1' answer this question:***<br>Has your child been admitted to hospital for a second time in the last six months?	○ Yes ○ No
	**---_SECOND ADMISSION_---**	

GB_InfantHealthSurvey - InfantOutpatientCare

Number	Question	Answers
	**The following questions ask if your child attended a hospital outpatient department/clinic for the LAST SIX MONTHS only. Please answer all questions as accurately as possible.**	
3.1	Has your child attended a hospital outpatient department/clinic in the last six months	○ Yes ◉ No
3.1.1	***If 'Has your child attended a hospital outpatient department/clinic in the last six months' is equal to 'Yes' answer this question:***<br>Which type of clinic did your child visit during the last six months?	○ Accident or Emergency<br>○ Paediatric / Neonatal Follow-up<br>○ Orthopaedic<br>○ Audiology / Hearing<br>○ Eye / Vision<br>○ General Medicine<br>○ Dermatology<br>○ Other
3.1.1.1	***If 'Which type of clinic did your child visit during the last six months?' is equal to 'Other' answer this question:***<br>Which specialty of outpatient department / clinic did your child visit during the last six months?	
3.1.2	***If 'Has your child attended a hospital outpatient department/clinic in the last six months' is equal to 'Yes' answer this question:***<br>What was the reason for this visit?	
3.1.3	***If 'Has your child attended a hospital outpatient department/clinic in the last six months' is equal to 'Yes' answer this question:***<br>How many times did your child visit this clinic during the past half year?	
3.2	CheckOPD	
3.2.1	***If 'CheckOPD' is equal to '1' answer this question:***<br>Has your child attended a second hospital outpatient department/clinic in the last six months	○ Yes ○ No
	**--_SECOND VISIT_--**	

TRUFFLE 2

Imperial College London

Doppler Image Scoring

For Umbilical Artery:

PULSEWAVE IMAGE (/6)	1 point	0 point
Magnification	Doppler display occupies 50% or more of the image	Doppler display occupies <50% of the image
Angle of insonation	<30 degrees	30 degrees or more
Sweep speed	Doppler spectrum has 4-10 waveforms	Doppler spectrum has < 4 (3 or less) or > 10 waveforms (11 or more)
Sample gate	Large enough to include 3/4th of the vessel diameter	Smaller than 3/4th of the vessel diameter
Appropriate PRF	The waveform fits at least 75% of the pulse wave Doppler scale or PRF (pulse repetition frequency)	The waveform fits < 75% of the pulse wave Doppler scale or PRF
Image quality	Uniform arterial waveforms, no aliasing or background artefacts or fetal breathing movements	Variable arterial waveforms/ aliasing/background artefacts/fetal breathing movements

For Middle Cerebral Arterial: (as above)

PULSEWAVE IMAGE (/6)	1 point	0 point
Magnification	Doppler display occupies 50% or more of the image	Doppler display occupies <50% of the image
Angle of insonation	<30 degrees	30 degrees or more
Sweep speed	Doppler spectrum has 4-10 waveforms	Doppler spectrum has < 4 (3 or less) or > 10 waveforms (11 or more)
Sample gate	Large enough to include 3/4th of the vessel diameter	Smaller than 3/4th of the vessel diameter
Appropriate PRF	The waveform fits at least 75% of the pulse wave Doppler scale or PRF	The waveform fits < 75% the pulse wave Doppler scale or PRF
Image quality	Uniform arterial waveforms, no aliasing or background artefacts or fetal breathing movements	Variable arterial waveforms/ aliasing/background artefacts/fetal breathing movements

For peer review only

BMJ Open

Perinatal and 2 year neurodevelopmental outcome in late preterm fetal compromise: the TRUFFLE 2 Randomised Trial Protocol

Journal:	*BMJ Open*
Manuscript ID	bmjopen-2021-055543.R1
Article Type:	Protocol
Date Submitted by the Author:	23-Sep-2021

Complete List of Authors:	Mylrea-Foley, Bronacha; Imperial College London, Institute of Reproductive and Developmental Biology, Department of Metabolism, Digestion and Reproduction; Imperial College Healthcare NHS Trust, Centre for Fetal Care, Queen Charlotte's & Chelsea Hospital, London<br>Arabin, Birgit; Humboldt University of Berlin, Department of Obstetrics Charite<br>Berger, Astrid; University Hospital of Innsbruck, Department of Obstetrics and Gynecology<br>bergman, Eva; Uppsala University, Department of Women's and Children's Health<br>Bhide, Amarnath; St George's University Hospitals NHS Foundation Trust, Fetal Medicine Unit<br>Bilardo, Caterina; Amsterdam UMC Locatie VUmc; University Medical Centre Groningen<br>Breeze, Andrew; Leeds Teaching Hospitals NHS Trust, Fetal Medicine Unit, Leeds General Infirmary, Leeds; University of Leeds, Leeds<br>Brodszki, Jana; Skanes universitetssjukhus Lund, Department of Obstetrics and Gynecology<br>Calda, Pavel; Charles University, Department of Obstetrics and Gynaecology<br>Cannings-John, Rebecca; Cardiff University Centre for Trials Research,<br>Cesari, Elena; Ospedale dei Bambini Vittore Buzzi, Department of Obstetrics and Gynaecology<br>Cetin, I; University of Milan, Department of Woman Mother and Child, Buzzi Children's Hospital<br>Dall'Asta, Andrea; University of Parma, Medicine and Surgery; Imperial College London Faculty of Medicine, Surgery and Cancer<br>Ebbing, Cathrine; Haukeland University Hospital, Department for Obstetrics and Gynecology<br>Ferrazzi, Enrico; Fondazione IRCCS Ca' Granda Ospedale Maggiore Policlinico, Clinical Sciences Medical School<br>Frusca, Tiziana; University of Parma, Department of Obstetrics and Gynaecology<br>Goodier, Jenny; Imperial College London, Obstetrics and Gynaecology, Queen Charlotte & Chelsea Hospital<br>Gyselaers, Wilfried; Ziekenhuis Oost-Limburg, Department of Obstetrics and Gynecology; Hasselt University,<br>Hecher, Kurt; University Medical Center Hamburg-Eppendorf, Department of Obstetrics and Fetal Medicine

Klaritsch, Philipp; Medical University of Graz, Department of Obstetrics and Gynaecology
Krofta, Ladislav; Institute for Care of Mother and Child
Lindgren, Peter; Karolinska Institutet, Department of Clinical Science, Intervention and Technology (CLINTEC); Karolinska University Hospital, Centre for Fetal Medicine (CFM)
Lobmaier, Silvia; Technical University of Munich, Department of Obstetrics and Gynecology, Klinikum rechts der Isar
Marlow, Neil; University College London, UCL EGA Institute for Women's Health
Marsal, K; Lund University, Obstetrics & Gynecology, Department of Clinical Sciences Lund
Maruotti, Giuseppe; Federico II University Hospital, Department of Neurosciences, Reproductive and Dentistry Sciences
Mecacci, Federico; University Hospital Careggi, High Risk Pregnancy Unit
Mullins, E; Imperial College London, metabolism, digestion and reproduction; Imperial College
Myklestad, Kirsti ; St Olavs Hospital University Hospital in Trondheim, Department of Obstetrics and Gynaecology
Napolitano, Raffaele; University College London, Elizabeth Garrett Anderson Institute for Women's Health; University College London Hospitals NHS Foundation Trust, Fetal Medicine Unit
Potter, Claire; Cardiff University, Centre for Trials Research
Prefumo, Federico; Università degli Studi di Brescia, Department of Obstetrics and Gynaecology
Raio, Luigi; University of Bern, Department of Obstetrics and Gynaecology
Richter, Jute; Katholieke Universiteit Leuven UZ Leuven, Department of Gynecology and Obstetrics
Sande, Ragnar; Stavanger University Hospital, Department of Obstetrics and Gynaecology
Stampalija, Tamara ; IRCCS Materno Infantile Burlo Garofolo, Unit of Fetal Medicine and Prenatal Diagnosis; University of Trieste, Department of Medicine, Surgery and Health Sciences
Thornton, Jim; University of Nottingham, Department of Obstetrics and Gynaecology, City Hospital
Townson, Julia; Cardiff University, Centre for Trials Research
Valensise, Herbert; University of Rome Tor Vergata, Department of Surgery, Division of Obstetrics and Gynecology, Policlinico Casilino
Visser, Gerard ; Utrecht University, Department of Obstetrics, University Medical Center
Wee, Ling; Princess Alexandra Hospital NHS Trust, Obstetrics And Gynaecology
Wolf, Hans; Amsterdam University Medical Centres, Department of Obstetrics and Gynaecology
Lees, Christoph; Imperial College London, Institute of Reproductive and Developmental Biology, Department of Metabolism, Digestion and Reproduction; Imperial College Healthcare NHS Trust, Centre for Fetal Care, Queen Charlotte’s & Chelsea Hospital, London

**Primary Subject Heading**:	Obstetrics and gynaecology
Secondary Subject Heading:	Paediatrics
Keywords:	Fetal medicine < OBSTETRICS, Maternal medicine < OBSTETRICS, Ultrasonography < OBSTETRICS

I, the Submitting Author has the right to grant and does grant on behalf of all authors of the Work (as defined in the below author licence), an exclusive licence and/or a non-exclusive licence for contributions from authors who are: i) UK Crown employees; ii) where BMJ has agreed a CC-BY licence shall apply, and/or iii) in accordance with the terms applicable for US Federal Government officers or employees acting as part of their official duties; on a worldwide, perpetual, irrevocable, royalty-free basis to BMJ Publishing Group Ltd ("BMJ") its licensees and where the relevant Journal is co-owned by BMJ to the co-owners of the Journal, to publish the Work in this journal and any other BMJ products and to exploit all rights, as set out in our licence.

The Submitting Author accepts and understands that any supply made under these terms is made by BMJ to the Submitting Author unless you are acting as an employee on behalf of your employer or a postgraduate student of an affiliated institution which is paying any applicable article publishing charge ("APC") for Open Access articles. Where the Submitting Author wishes to make the Work available on an Open Access basis (and intends to pay the relevant APC), the terms of reuse of such Open Access shall be governed by a Creative Commons licence – details of these licences and which Creative Commons licence will apply to this Work are set out in our licence referred to above.

Other than as permitted in any relevant BMJ Author's Self Archiving Policies, I confirm this Work has not been accepted for publication elsewhere, is not being considered for publication elsewhere and does not duplicate material already published. I confirm all authors consent to publication of this Work and authorise the granting of this licence.

For peer review only

Title:

Perinatal and 2 year neurodevelopmental outcome in late preterm fetal compromise: the TRUFFLE 2 Randomised Trial Protocol

Authors

Bronacha Mylrea-Foley[1], Birgit Arabin[2], Astrid Berger[3], Eva Bergman[4], Amarnath Bhide[5], Caterina M Bilardo[6], Andrew Breeze[7], Jana Brodszki[8], Pavel Calda[9], Rebecca Cannings-John[10], Elena Cesari[11], Irene Cetin[11], Andrea Dall'Asta[12], Catherine Ebbing[13], Enrico Ferrazzi[14], Tiziana Frusca[12], Jenny Goodier[1], Wilfred Gyselaers[15], Kurt Hecher[16], Philipp Klaritsch[17], Ladislav Krofta[18], Peter Lindgren[19], Silvia M Lobmaier[20], Neil Marlow[21], Karel Marsal[22], Giuseppe M Maruotti[23], Federico Mecacci[24], Edward Mullins[1], Kirsti Myklestad[25], Raffaele Napolitano[21], Claire Potter[10], Federico Prefumo[26], Luigi Raio[27], Jute Richter[28], Ragnar Kvie Sande[29], Tamara Stampalija[30], Jim G Thornton[31], Julia Townson[10], Herbert Valensise[32], Gerard HA Visser[33], Ling Wee[34], Hans Wolf[35], Christoph C Lees[1]* and on behalf of the **TRUFFLE 2 Investigators Group**

*Corresponding author

Prof Christoph C Lees, c.lees@imperial.ac.uk

Imperial College Healthcare NHS Trust
Centre for Fetal Care, Queen Charlotte's & Chelsea Hospital
Du Cane Road
London
W12 0HS

1 Imperial College London, Obstetrics and Gynaecology, Queen Charlotte's & Chelsea Hospital London, London, UK
2 Department of Obstetrics Charite, Humboldt University of Berlin, Berlin, Germany
3. Department of Gynecology and Obstetrics, Medical University of Innsbruck, Innsbruck, Austria
4. Department of Women's and Children's Health, Uppsala University, Uppsala, Sweden
5. Fetal Medicine Unit, St George's University Hospitals NHS Foundation Trust, London, UK
6. Department of Obstetrics Amsterdam, Vrije Universiteit Amsterdam, Noord-Holland, Netherlands
7. Fetal medicine Unit, Leeds Teaching Hospitals NHS Trust, Leeds, UK
8. Department of Obstetrics and Gynecology, Lund Skanes universitetssjukhus Lund, , Skåne, Sweden
9. Department of Obstetrics and Gynaecology, Charles University, Praha, Czech Republic
10. Centre for Trials Research, Cardiff University, Cardiff, UK
11. Department of Obstetrics and Gynecology, Vittore Buzzi Hospital, University of Milan, Italy
12. Medicine and Surgery, University of Parma, Parma, Italy
13. Universitetet i Bergen, Bergen, Norway
14. Department of Clinical Sciences and Community Health, Fondazione IRCCS Ca' Granda Ospedale Maggiore Policlinico, Italy
15. Department of Obstetrics and Gynecology, Hospital Oost-Limburg, Genk, Belgium
16. Department of Obstetrics and Fetal Medicine, University Medical Center Hamburg-Eppendorf, Hamburg, Germany

For peer review only - http://bmjopen.bmj.com/site/about/guidelines.xhtml

17. Division of Obstetrics and Maternal Fetal Medicine, Medical University of Graz, Graz, Austria
18. Institute for Care of Mother and Child, Prague, Czech Republic
19 Division of Obstetrics and Gynecology, Department of Clinical Science, Intervention & Technology (CLINTEC), Karolinska Institutet, Stockholm, Sweden
20. Department of Obstetrics and Gynecology, Klinikum rechts der Isar, Technical University of Munich, Germany
21. Elizabeth Garrett Anderson Institute for Women's Health University College London, London, UK
22. Obstetrics and Gynaecology, Faculty of Medicine, Lunds Universitet, Lund, Sweden
23. Department of Neurosciences, Reproductive and Dentistry Sciences, Federico II University Hospital, Napoli, Italy
24. High Risk Pregnancy Unit, University Hospital Careggi, Firenze, Italy
25. Department of Obstetrics, Children`s and Women`s Health, St Olavs Hospital University Hospital, Trondheim, Norway
26. Department of Obstetrics and Gynaecology, Università degli Studi di Brescia, Brescia, Italy
27. Department of Obstetrics and Gynaecology, University of Bern, Bern, Switzerland
28. Department of Gynecology and Obstetrics, Katholieke Universiteit Leuven, Leuven, Belgium
29. Department of Obstetrics and Gynaecology, Stavanger University Hospital, Stavanger, Norway
30. Unit of Fetal Medicine and Prenatal Diagnosis, RCCS materno infantile Burlo Garofolo Dipartimento di Pediatria, Trieste, Italy
31. Department of Obstetrics and Gynaecology, University of Nottingham, City hospital, Nottingham, UK
32. Division of Obstetrics and Gynaecology Policlinico Casilino, Roma, Italy
33. Department of Obstetrics, University Medical Center, Utrecht University, Utrecht, Netherlands
34. Obstetrics And Gynaecology, Princess Alexandra Hospital NHS Trust, UK
35. Department of Obstetrics and Gynaecology, Amsterdam University Medical Centres, Amsterdam, Netherlands

For peer review only

Word Count:

4315

For peer review only - http://bmjopen.bmj.com/site/about/guidelines.xhtml

TRUFFLE 2 Investigator Group List:

Ajlana Mulic-Lutvica, Alexey Kholin, Alice Zavatta, Andrea Smith, Andrej Černý, Andrew Breeze, Andrew Sharp, Andy Simm, Angela Ramoni, Angelo Sirico, Anke Diemert, Antonio Lanzone, Aris Papageorghiou, Barry Lloyd, Bianca Masturzo, Christina Ammari, Christine Morfeld, Christopher Lloyd, Claudia Seidig, Constantin Von Keisenberg, Dagmar Pilz, Damiano Lopresti, Daniele Farsetti, Danielle Thornton, Danilo Buca, Dietmar Schlembach, Ekkehard Schleußner, Elena Mantovani, Emanuela Taricco, Emma Bertucci, Enrico Ferrazzi, Erich Cosmi, Eva Ostermayer, Ferenc Macsali, Francesca Ferrari , Francesca Presti, Francesco D'Antonio, Francesco Picciotto, Gareth Waring, Georgios Rembouskos, Giuseppe Cali, Giuseppe Rizzo, Grazia Maria Tiralongo, Ilaria Fantasia, Ilaria Giuditta Ramezzana, Ilenia Mappa, Ioannis Kyvernitakis, Iris Derwig, Iris Dressler-Steinbach, Jiří Vojěch, Jørgen Linde, Julia Binder, Karen Melchiorre, Kate Walker, Kinga Chalubinski, Kristiina Rull, Kristina Kernell, Lars Brodowski, Laura Sarno, Ligita Jokibkiene, Liina Rajasalu, Lina-Ana Fryszer, Louisa Jones, Magdalena Bednarek-Jędrzejek, Makrina Savvidou, Maria Stefopoulou, Marianna Rambaldi, Mark Kilby, Nia Jones, Nicola Fratelli, Nishigandh Deole, Patrick Greimel, Petra Pateisky, Pilar Palmrich, Ralf Schild, Roland Devlieger, Sabina Ondrová, Sarah Gumpert, Sasha Taylor, Saskia Schmidt, Sebastian Kwiatkowski, Serena Caterina, Serena Ottanelli, Serena Simeone, Sergio Ferrazzani, Silvia Salvi, Silvia Visentin, Sofia Amylidi-Mohr, Spyros Bakalis, Stefan Verlohren, Susanne Dargel, Sven Kehl, Tanja Groten, Tatjana Radaelli, Tinne Mesens, Tiziana Fanelli, Torbjørn Eggebø, Tullio Ghi, Tuuli Haabpiht, Wassim Hassan, Yvonne Heiman, Zulfiya Khodzhaeva

For peer review only

Abstract:

Introduction Following the detection of fetal growth restriction, there is no consensus about the criteria that should trigger delivery in the late preterm period. The consequences of inappropriate early or late delivery are potentially important yet practice varies widely around the world, with abnormal findings from fetal heart rate monitoring invariably leading to delivery. Indices derived from fetal cerebral Doppler examination may guide such decisions although there are few studies in this area. We propose a randomised, controlled trial to establish the optimum method of timing delivery between 32 weeks and 36 weeks 6 days of gestation. We hypothesise that delivery on evidence of cerebral blood flow redistribution reduces a composite of perinatal poor outcome, death and short-term hypoxia-related morbidity, with no worsening of neurodevelopmental outcome at 2 years.

Methods and Analysis Women with non-anomalous singleton pregnancies 32+0 to 36+6 weeks of gestation in whom the estimated fetal weight or abdominal circumference is <10th percentile or has decreased by 50 percentiles since 18-32 weeks will be included for observational data collection. Participants will be randomised if cerebral blood flow redistribution is identified, based on umbilical to middle cerebral artery pulsatility index ratio (UCR) values. Computerised cardiotocography (cCTG) must show normal fetal heart rate short term variation (STV ≥4.5msec) and absence of decelerations at randomisation. Randomisation will be 1:1 to immediate delivery or delayed delivery (based on cCTG abnormalities or other worsening fetal condition). The primary outcome is poor condition at birth and/or fetal or neonatal death and/or major neonatal morbidity, the secondary non-inferiority outcome is two-year infant general health and neurodevelopmental outcome based on the Parent Report of Children's Abilities-Revised (PARCA-R) questionnaire.

Ethics and Dissemination The Study Coordination Centre has obtained approval from London – Riverside Research Ethics Committee (REC) and Health Regulatory Authority (HRA). Publication will be in line with NIHR Open Access policy.

Registration details;

MAIN SPONSOR: Imperial College London, Reference: 19QC5491

FUNDERS: NIHR HTA, Reference: 127976

STUDY COORDINATION CENTRE: Imperial College Healthcare NHS Trust, Du Cane Road, London, W12 0HS with Centre for Trials Research, College of Biomedical & Life Sciences, Cardiff University

IRAS Project ID: 266400

REC reference: 20/LO/0031

ISRCTN registry: 76016200

For peer review only - http://bmjopen.bmj.com/site/about/guidelines.xhtml

For peer review only

For peer review only - http://bmjopen.bmj.com/site/about/guidelines.xhtml

Strengths and limitations of this study:

Strengths

- Changes in cerebral Doppler measures are considered a strong candidate for triggering delivery following detection of late preterm fetal growth restriction.
- The primary outcome is a composite of peri- and neonatal outcome, and infants will be followed to determine health and two-neurodevelopment at 2 years to ensure safety and non-inferiority of intervention.
- This appropriately powered UK and European multicentre trial will inform NHS policy and the findings will be generalizable to late preterm fetal growth restriction in other healthcare systems.

Limitations

- There is limited data available in respect of selecting the threshold of cerebral Doppler change for randomization.
- Some non UK centres will not randomize women at the earlier gestation range (32-33+6 weeks) and this could limit the generalisability of the results in this group of women.

For peer review only

For peer review only - http://bmjopen.bmj.com/site/about/guidelines.xhtml

Introduction:

Third trimester poor fetal growth and compromise are strongly associated with stillbirth, neonatal illness[1] and an increased risk of fetal or neonatal brain injury[2]. The only therapeutic option is delivery of the fetus. This poses a dilemma: delivery too early may incur the complications of prematurity, delivery too late risks further fetal compromise, brain injury and late stillbirth. The problem of when to deliver these babies is twofold: there is no consensus on how to identify fetal compromise and there is no 'ideal' evidence-based monitoring strategy. Current screening strategies include standardised symphysis-fundal height measurement, third trimester ultrasound and umbilical Doppler velocimetry[3,4]. NICE acknowledges that methods to identify fetal growth restriction (FGR) are 'poorly developed or not tested by rigorous methodology'.

The use of Doppler ultrasound of fetal vessels allows non-invasive assessment of blood flow. Fetal 'cerebral blood flow redistribution', prioritising blood flow toward the brain at the expense of other organs, is a response to an adverse intrauterine environment characterised by hypoxemia. Ultrasound markers of such fetal compromise include Doppler assessment of blood flow velocity[5–7] and abdominal circumference growth velocity 'drop off' in the third trimester[8,9]. Intervention by delivery of the fetus in response to either evidence of blood flow redistribution or fetal growth slowing has never been tested in a randomised trial. Computerised cardiotocography (cCTG) has been used in several observational studies to determine fetal condition in relation to hypoxaemia and acidosis. The RCOG recommends that research is required to evaluate the effectiveness of third trimester ultrasound assessment, concluding that 'middle cerebral artery Doppler may be a more useful test in small for gestational age fetuses detected after 32 weeks'[4] but does not define parameters that should trigger delivery. Even with effective screening for fetal growth restriction, the questions of how to monitor and when to deliver would remain.

A Cochrane review of the management of 'compromised babies' at term showed no difference in perinatal or long-term outcome with a policy of early delivery versus conservative management[10]. Only three trials were included: two included small babies, both part of the DIGITAT study (a small pilot and the main trial[11]), the third included babies with reduced amniotic fluid. There is no Cochrane review on the optimal timing of delivery in late preterm growth restricted babies.

A systematic review included only one trial of timed delivery in late preterm babies. The Growth Restriction Intervention Trial[12] included 547 babies from 24-36 weeks of gestation with evidence of preterm growth restriction or compromise, where there was clinical uncertainty whether immediate delivery was indicated. Of these, 210 babies were recruited between 33+0 and 36+6 weeks: 107 women were randomised to early delivery and 103 to delayed delivery. Mortality and a range of neurodevelopmental measures were similar between the groups. These results cannot be used to inform management because of the small number of infants assessed using only umbilical artery Doppler velocimetry, the subjective assessment of the cardiotocograph (i.e. not computerised analysis), and the use of clinician's discretion rather than standardised management.

Results from our previous randomised trial of delivery decision-making using Doppler velocimetry and cCTG in pregnancies with FGR at 26-32 weeks of gestation - the TRial of Umbilical Fetal FLow in Europe (TRUFFLE) provided evidence associating monitoring strategies with improved outcomes[13] and guides practice internationally[14,15]. In TRUFFLE (2005-2010) we studied FGR <32 weeks of gestation using cCTG as the standard of care for timing delivery, compared to early or late fetal ductus venosus (DV) Doppler velocimetry changes with a cCTG 'safety net'. Surviving infants whose mothers had been

For peer review only - http://bmjopen.bmj.com/site/about/guidelines.xhtml

randomised to delivery in late ductus venosus changes arm showed better neurodevelopment at 2 years of age[13].

We then carried out the prospective multicentre observational TRUFFLE 2 Feasibility Study (2017-18, n=1024). A range of markers of cerebral redistribution were evaluated as potential delivery trigger points in two gestational age bands. These included the middle cerebral artery (MCA) Doppler and the umbilical cerebral ratio (UCR); calculated by dividing the Doppler pulsatility index of the umbilical artery by that of the MCA. Participating clinicians suggested greater concern about fetal condition is required to trigger delivery at earlier rather than later gestational ages between 32 and 36 weeks. We therefore selected a more abnormal threshold for cerebral redistribution at earlier gestational ages, based on stepped UCR z-score values[16]. Of note was the finding from a prospective study that birth condition, fetal mortality and neonatal morbidity rate were more common among fetuses showing higher UCR z-score, indicating cerebral redistribution, compared to those with a lower UCR z-score (15 vs. 9%)[17].

On this basis, we designed this randomised trial of delivery for women identified with late preterm fetal growth restriction, to test the hypothesis that delivery based on UCR values derived from the feasibility study will safely lead to improved fetal outcomes (TRUFFLE 2).

Methods and analysis:

Study Design

This is an individual unblinded randomised trial of pregnant women experiencing fetal growth restriction. Following informed consent, 1560 women will be randomised to either immediate or delayed delivery. Women are being recruited from UK and European centres.

Potential participants are women with singleton non-anomalous pregnancies between 32+0 and 36+6 weeks of gestation in whom a small for gestational age (SGA) fetus is identified or one whose growth has slowed. This is defined as estimated fetal weight or abdominal circumference <10th percentile and/or having decreased by 50 percentiles since an ultrasound scan at 18-32 weeks. Each centre will utilise local growth charts. Data is collected on absolute measurements and growth chart-derived centiles. Once identified as potential participants women receive regular monitoring as per local standard of care for fetal condition, using ultrasound biometry, umbilical and middle cerebral artery (MCA) Doppler velocimetry assessments and cCTG (using Dawes-Redman criteria). This is recommended to be repeated every 14 days; these observational data are recorded.

Women become eligible for randomisation when signs of fetal cerebral blood flow redistribution are detected by Doppler, defined below and shown in the Study Flowchart (Supplementary File 1 - Flowchart).

Delivery is based on UCR pulsatility index z-scores, of >1.5 (between 32+0 and 33+6 weeks) or >1.0 (34+0 to 36+6 weeks). These correspond to an absolute UCR of ≥1.0 at 32+0 to 33+6 weeks and ≥0.8 at 34+0 to 36+6 weeks. Abnormal UCR measurements must be repeated within 2–24 hours to confirm these values. Randomisation will occur at the time of the second qualifying UCR measurement (this need not be consecutive) and is stratified by centre and gestational age. Randomisation is through the CASTOR website (Amsterdam, NL) into which eligibility, monitoring and outcome data are entered.

For peer review only - http://bmjopen.bmj.com/site/about/guidelines.xhtml

Women are consented in a two-stage process. Stage 1 (pre-eligible) consent is not a prerequisite and women may be consented directly for randomisation with stage 2 (eligible for randomisation) consent.

Pre-eligible – Consent for Observational Data Collection

1. Consented for prospective data collection once identified as meeting criteria for SGA or slowed fetal growth (as defined above) but not meeting cerebral Doppler thresholds for randomisation. This will include demographics, medical history, ultrasound findings and outcomes. This consent will include obtaining a personal email address, which is entered into and stored on Castor, and willingness to be contacted in the future for follow up where women are randomised. (Supplementary File 2 – Consent Form for Observational Data Collection)

Eligible for randomisation – Consent for Randomisation

2. Consent for randomisation once cerebral redistribution is identified, with UCR (as defined above) of ≥1.0 at 32+0 to 33+6 weeks and ≥0.8 at 34+0 to 36+6 weeks. This consent will also include contact details and willingness to be contacted in the future for follow up data collection. (Supplementary File 3 – Consent for Randomisation)

Women are randomly allocated to either immediate delivery or delayed delivery as defined below. Randomisation has a 1:1 allocation ratio and stratified based on gestational age (above or below 34 weeks) and centre. Randomisation is conducted on the electronic data capture platform (Castor EDC, Amsterdam, The Netherlands).

Immediate Delivery

Participants in the immediate delivery arm will be delivered by Caesarean or induction of labour will be commenced within 48 hours, allowing for administration of corticosteroids and infusion of magnesium sulphate as per local protocol and guidance. Start of induction of labour is defined as administering cervical preparation (cervical balloon, prostaglandins, etc) or artificial rupture of membranes or administration of oxytocin.

Delayed Delivery

Participants in the delayed delivery arm will be monitored using twice weekly Doppler and cCTG monitoring, or more frequently based on local clinical protocols if required. Umbilical artery Doppler velocities may be measured in this time and delivery may be based on these safety net criteria (see box below). We strongly recommend that MCA measurements are not undertaken during monitoring in the delayed delivery arm. Delivery is indicated when short term variation (STV) is <4.5 ms on cCTG or there are repeated fetal heart rate decelerations. Once participants reach 37^{+0} weeks of gestation the delivery plan will be based on local protocols.

For peer review only - http://bmjopen.bmj.com/site/about/guidelines.xhtml

Umbilical Doppler delivery thresholds

In all arms absolute indications for delivery include:

- umbilical artery Doppler with reversed end diastolic flow after entry into the trial, OR
- umbilical artery Doppler absent end diastolic flow from 34^{+0} weeks

Box 1: Delivery thresholds based on umbilical artery Doppler for participants in all arms of the study

Study Timeline

Study set up: 0-10 months; recruitment/randomisation: 11-36 months. Two years follow up questionnaires 35-60 months; analyses, writing up, reporting & dissemination: 61-66 months. This equates to 5 and ½ years (66 months).

Study outcome measures

Primary outcome

The primary outcome for the study is a composite of fetal or infant death, composite measure of poor condition at birth and neonatal morbidity – as defined by presence of any of the following:

1) Poor condition at birth

- Apgar score at 5 minutes <7, umbilical artery pH <7.0 or umbilical vein pH <7.1
- Need for resuscitation with intubation, chest compressions or medication

2) Fetal death/ death before neonatal hospital discharge

3) Neonatal brain injury syndromes

- Infants with a diagnosis consistent with hypoxic ischaemic encephalopathy (HIE): term and near-term infants only
- Infants with a diagnosis of intracranial haemorrhage, perinatal stroke, HIE, central nervous system infection, or kernicterus (bilirubin encephalopathy): all infants
- Preterm white matter disease (periventricular leukomalacia): preterm infants only
- Infants with a recorded seizure confirmed by EEG

4) Respiratory support

- Need for mechanical support of respiration after admission to neonatal unit (NNU), for more than 1 hour; includes need for continuous positive airways pressure (CPAP or NIPPV) or mechanical ventilation via endotracheal tube but excludes need for supplemental oxygen.

5) Cardio-vascular abnormality

- Hypotensive treatment, patent ductus arteriosus requiring treatment, or disseminated coagulopathy;

6) Sepsis

- Clinical sepsis with positive blood culture, or
- necrotising enterocolitis requiring surgery

For peer review only - http://bmjopen.bmj.com/site/about/guidelines.xhtml

For peer review only

7) Retinopathy of prematurity requiring treatment (laser or anti-VEGF injections)

Secondary outcomes

For the baby:

1) *Health and developmental outcomes* – assessed using Parent Report of Children's Abilities-Revised (PARCA-R) questionnaire at 2 years corrected age and an Infant Health Questionnaire up to 2 years (Supplementary File 4 – PARCA-R Questionnaire and Supplementary File 5 – Infant Health Questionaire).

The PARCA-R [18] will be completed at 24 months term-equivalent age, this allows derivation of the non-verbal cognitive scale and language development scale. Raw scores from the scales are standardised (by corrected age and gender) to a notional population mean of 100 SD=15 and the average of these two component scores will be taken as the overall composite score. Corrected age is used for preterm babies (born before 37 weeks) and represents the age of the child from the estimated date of delivery.

The Infant Health Questionnaire [19] will be used to derive the following health outcomes at 6, 12, 18 and 24 months post-partum:

- Use of any hospital service (yes/no) and total number of contacts over the 2 year period;
- Admitted to hospital (yes/no) and total number of admissions over the 2 year period;
    - Planned/unplanned admissions to hospital (yes/no) over the 2 year period;
    - Intensive care or not over the 2-year period;
- Attended Emergency Department (and not subsequently admitted) (yes/no) over the 2 year period;
- Attended Outpatients/clinic (yes/no) over the 2 year period.

For the mother:

1) Gestational hypertension developed post study entry

- As defined by the International Society for Study of Hypertension in pregnancy (ISSHP): hypertension (blood pressure ≥140/90mmHg) arising de novo after 20 weeks gestation in the absence of proteinuria

2) Pre-eclampsia developed post study entry

- As defined by the ISSHP: blood pressure ≥140/90mmHg AND significant proteinuria (protein/creatinine ratio of 30 mg/mmol or more)

3) Onset of labour (spontaneous, induction (method), prelabour caesarean section)

4) Mode of delivery (spontaneous vaginal, assisted vaginal, caesarean section)

Doppler Quality Control

Sonographer Standardisation: Each sonographer in each centre taking part in the trial are assessed by the local PI. Each sonographer will submit to the local PI two images: pseudo anonymised

For peer review only - http://bmjopen.bmj.com/site/about/guidelines.xhtml

ultrasound images for each Doppler parameter (umbilical artery and MCA) each showing a colour Doppler image with the gate placed over the vessel, and the pulsed wave Doppler waveform arising from that image. The local PI will determine whether these images are satisfactory using a predefined quality control scoring system (Supplementary File 6 – Doppler Image Scoring Sheet).

Doppler Ultrasound Criteria: Measurements are obtained in fetuses between 32+0 and 36+6 weeks of gestation. Umbilical artery and MCA pulsatility index images are collected according to specific predefined objective criteria for both the colour Doppler images and pulsed wave Doppler.

Doppler Quality Control: The local PI will provide details of all sonographers having undergone standardisation in that centre to the Centre for Trials Research (CTR). The CTR will independently request all images submitted to the local PI for the first 5 patients randomised from each unit, and then for up to 10% of patients thereafter, for anonymised quality control assessment by the Quality Control Board. All images are collected as pseudo anonymised .jpeg images and saved electronically in a Doppler ultrasound sonographer standardisation file by the PI, with the submitting sonographer identifiable. Images are scored using the predefined scoring criteria. The CTR will manage this process and will provide feedback if necessary, and ensure that members of the TRUFFLE 2 Quality Control Board do not assess images from their own unit.

Participant Entry

Pre-registration evaluations

- Ultrasound scan of fetal growth between 32+0 and 36+6 weeks of gestation, including measurement of MCA and umbilical artery Doppler velocities
- cCTG STV analysed using Dawes-Redman criteria

Inclusion Criteria

(All criteria should be fulfilled to be eligible for randomisation)

- Women ≥ 18 years old
- Pregnant with singleton non-anomalous fetus
- Between 32+0 and 36+6 weeks of gestation
- Estimated fetal weight or abdominal circumference <10th percentile OR decreased by 50 percentiles since an ultrasound scan at 18+0-32+0 weeks and:
- Cerebral redistribution defined as UCR ≥1.0 (between 32+0 and 33+6 weeks) or ≥0.8 (34+0 to 36+6 weeks) **repeated within 2-24 hours**
- Normal STV on cCTG (4.5msec or above)

Exclusion Criteria

- Indication for immediate delivery required within 48 hours
- Unable to give informed consent
- Preterm pre-labour rupture of the membranes
- Suspected placental abruption or antepartum haemorrhage
- Presence of reversed end diastolic flow in the umbilical artery.

For peer review only - http://bmjopen.bmj.com/site/about/guidelines.xhtml

Assessment and Follow-up

Assessment of the primary outcome is at infant discharge from the neonatal unit and assessment of the key secondary infant outcomes will use the Infant Health Questionnaire and PARCA-R. The Infant Health Questionnaire will be sent out by investigators via Castor at 6, 12, 18 and 24 months post-partum. Neurodevelopment is assessed at 2 years age corrected for prematurity using the PARCA-R, also sent via Castor (translated and validated into local language). The window for determining 2 year outcome is from 22-28 months over which range the PARCA-R has been standardised.

Endpoint is 30 months after the estimated date of delivery of the last participant to deliver (24 months follow-up with additional 6 months for data cleaning and additional enquiries).

Statistics and Data Analysis

Sample size

The trial is powered to detect if immediate delivery following cerebral redistribution is superior to expectant management following cerebral redistribution on this outcome. A difference in the proportion with the primary outcome from 15% in the delayed delivery to 9% in the immediate delivery (from TRUFFLE 2 feasibility study) demonstrates an odds ratio of 0.56. At 2-sided 5% significance with 95% power, 780 participants per arm are required, giving 1,560 in total. Given the immediacy of this outcome, no loss to follow-up is expected. An important non-inferiority secondary safety outcome is infant neurodevelopment, which is measured by parent completion questionnaire at 2 years using the PARCA-R, as recommended in NICE Guidance [20], supplemented by infant health information over the intervening 2 years. Assuming a loss to follow-up at 2 years of 20%, 2 year outcomes for approximately 1,248 infants are expected (624 per group assuming no difference in the loss to follow-up between the groups). The PARCA-R questionnaire provides a composite score for neurodevelopment with a standardised mean of 100 and standard deviation of 15. With a one-sided significance level of 1%, under a non-inferiority hypothesis, a sample size of 624 in each group achieves a 98% power to detect a non-inferiority margin of difference in the mean PARCA-R score of no less than 4 points (0.25 of a standard deviation). A margin of no less than 3 points can be detected with 90% power.

Main analysis

The primary analysis approach for the primary outcome of composite of adverse fetal/neonatal outcomes, maternal secondary outcomes and the Infant Health Questionnaire will be intention to treat with participants analysed in the groups to which they are assigned regardless of deviation from the protocol or intervention received. The PARCA-R will be both an intention to treat and a per protocol analysis, since the hypothesis under examination for these outcomes is a non-inferiority hypothesis. The per-protocol analysis will exclude babies of women who do not receive their intervention as planned. As the trial includes multiple centres (and will involve a reasonable number of participants randomised per centre), the analysis will be based on the individual participant, allowing for clustering between participants within centre using robust standard errors. All analyses will additionally adjust for gestational age at inclusion (stratification risk factor used in randomisation) as a fixed factor. For binary outcomes (composite of adverse fetal/neonatal outcomes, gestational hypertension, pre-eclampsia) a logistic regression model will be used to compare this outcome by arm and results presented as odds ratios and two-sided 95% confidence interval (CI). Continuous outcomes (PARCA-R, Infant Health Questionnaire) will be analysed using linear regression and results presented

For peer review only - http://bmjopen.bmj.com/site/about/guidelines.xhtml

as adjusted differences in means alongside 95% CIs. The Infant Health Questionnaire will also be examined over time using a repeated measures model, and will include an interaction term for time (6, 12, 18 and 24 months) and trial arm to investigate any divergent or convergent pattern in Infant Health Questionnaire. The categorical outcomes of mode of delivery and onset of labour will be compared between trial arms by fitting a multilevel ordinal regression model. The neonatal secondary outcome will be examined using the mean PARCA-R score between each trial arm using linear regression, and a one-sided 95% CI constructed to assess non-inferiority. Additional pre-specified sub-group analyses will be carried out to analyse the primary outcome in the whole cohort by those that are <10th centile (versus ≥10th) and <3rd centile (versus ≥3rd) based on Intergrowth-21[21], WHO[22] and customised growth charts, maternal morbidity (yes/no) and corticosteroid administration (yes/no). If numbers are sufficient, we will also describe the intervention effect in the sub-groups <3rd centile versus 4-10th versus >10th centile but will not statistically tested for intervention effect.

All analyses will be undertaken after database lock following data collection at 2 years. No interim analyses are planned. Missing outcome data but will be accounted for in sensitivity analyses using multiple imputation, where we will assume that outcome data are missing at random given the observed measurements. All planned analyses will be described in detail in a Statistical Analysis Plan (SAP), which will be finalised prior to database lock. The reporting of findings will be in accordance with the CONSORT guidelines for RCTs. Statistical analysis will be performed in Stata (version 16 or higher).

Data Collection:

Data collection and randomisation are carried out on a secure cloud-based electronic data capture platform (Castor EDC, Amsterdam, The Netherlands). Participants can only be identified by their recruiting centers using unique trial identifiers. Participant email addresses will be stored for sending follow up questionnaires, this is stored within an encrypted section of Castor only their recruiting center has access to.

Trial monitoring:

A Trial Management Group will meet monthly to review study progress and recruitment targets. In addition there will be a Trial Steering Committee and Data Monitoring Committee, with a majority of independent members, to guarantee the safety of study participants.

A clinical trial risk assessment has been developed by Centre for Trials Research at Cardiff University, to determine the intensity and focus of central and on-site monitoring activity in the TRUFFLE 2 trial. Appropriate monitoring levels will be employed and are fully documented in the trial monitoring plan. Investigators should agree to allow trial related monitoring, including audits and regulatory inspections, by providing direct access to source data/documents as required. Findings generated from on-site and central monitoring will be shared with the Sponsor, CI, PI & local R&D.

Serious Adverse Events (SAE) will be reported for randomised participants and their infants. The reporting period is from the point of randomisation until discharge home for the mother and neonate, maternal death is to be reported up to 42 days after delivery. SAEs will be reported on the data capture platform (Castor) in accordance with the UK policy framework for Health and Social care research.

For peer review only - http://bmjopen.bmj.com/site/about/guidelines.xhtml

Patient and Public Involvement:

Patient and Public Involvement (PPI) has been embedded throughout the development of this study.

Sands, the Stillbirth and Neonatal Death charity in the UK, have been closely involved in the development and design of this study. Sands is clear that there is an urgent need for better assessment and care of women and their babies in late pregnancy and are fully supportive of the study. They will play an integral role throughout the study and in publicising the study and disseminating results in accessible formats. The research and development lead at Sands, is a member of the Trial Management Group. In their role, they represents many members of the public affected by stillbirth and neonatal death.

An FGR PPI panel was convened to discuss TRUFFLE 2 study design. The purpose of the workshop was to ask members of the public for their views on the study, with specific focus on the concept of randomisation, the current situation for the treatment of FGR, and the scope of this study. The FGR panel comprised eight women, who had experienced FGR, stillbirth or uncomplicated pregnancies. At the workshop the proposed study design was discussed. Following the workshop, the women were asked to complete an anonymous questionnaire relating to early delivery compared to monitoring the health of the baby in the womb and randomisation. Overall, women were supportive of the study, one stated "I believe a lot of women will benefit from this study'. Another stated 'The study will have an impact; it will act as a ripple effect on the family and wider public for the better.' Regarding randomisation, most women said that they would be happy to be randomised to either arm whilst acknowledging that they would need support, one woman stated 'Yes, I would be randomised but would need the right support from the clinical staff. I would need to have 100% trust in my doctors'. Two women from this panel have agreed to be involved in the management of the study as independent members of the Trial Steering Committee. We incorporated the views and feedback of the PPI panel in the drafting of the patient information leaflet and consent forms to ensure clarity and comprehensibility.

Ethics and dissemination:

This protocol was written in accordance with the SPIRIT (Standard Protocol Items: Recommendations for Interventional Trials) 2013 Statement (see Supplementary File 7 - SPIRIT Checklist). The Study Coordination Centre has obtained approval from the London - Riverside Research Ethics Committee (REC) and Health Regulatory Authority (HRA). The study must also receive confirmation of capacity and capability from each participating NHS Trust before accepting participants into the study or any research activity is carried out. The study has also been submitted to research ethics committees in all participating countries, and each centre must confirm local ethical and hospital approval before starting recruitment. The study will be conducted in accordance with the recommendations for physicians involved in research on human subjects adopted by the 18th World Medical Assembly, Helsinki 1964 and later revisions. Publication will be in line with NIHR Open Access policy.

Authors' contributions:

BMF drafted and revised the protocol. BA, A Berger, EB, A Bhide, CMB, A Breeze, JB, PC, EC, IC, ADA, CE, EF, TF, WG, KH, PK, LK, PL, SML, NM, K Marsal, GMM, FM, K Myklestad, RN, FP, LR, JR, RKS, TS, JGT, HV, GHV and LW contributed intellectually to the development of the protocol and revised the draft protocol. EM, JG, CP and JT revised the draft protocol. RCJ wrote the statistical analysis plan.

For peer review only - http://bmjopen.bmj.com/site/about/guidelines.xhtml

HW designed the data collection database, wrote the statistical analysis plan, contributed intellectually to the development of the protocol and revised the draft protocol. CCL initiated the collaborative project, led the development of the protocol and revised the draft protocol. All authors approved the final version for publication.

All members of the TRUFFLE 2 Investigator Group collaborated on the study.

Funding statement:

This research was funded by the National Institute for Health Research (NIHR) (127976). CCL is supported by the NIHR Biomedical Research Centre (BRC) based at Imperial College Healthcare NHS Trust and Imperial College London. BMF was supported by a Fellowship Grant from Imperial Health Charity and NIHR Imperial BRC (RFRFPrD1920/104). The views expressed are those of the author(s) and not necessarily those of the NHS, the NIHR or the Department of Health and Social Care.

Competing interests statement:

All authors have completed the ICMJE uniform disclosure form at www.icmje.org/coi_disclosure.pdf and declare: no financial relationships with any organisations that might have an interest in the submitted work in the previous three years; no other relationships or activities that could appear to have influenced the submitted work.

Full references:

1 Baschat AA. Planning management and delivery of the growth-restricted fetus. *Best Pract Res Clin Obstet Gynaecol* 2018;**49**:53–65. doi:10.1016/j.bpobgyn.2018.02.009

2 Malhotra A, Ditchfield M, Fahey MC, *et al.* Detection and assessment of brain injury in the growth-restricted fetus and neonate. *Nat Publ Gr* 2017;**82**:184–93. doi:10.1038/pr.2017.37

3 NICE. Antenatal care for uncomplicated pregnancies | Guidance. 2019.

4 RCOG. Small-for-Gestational-Age Fetus, Investigation and Management (Green-top Guideline No. 31). 2013.

5 Meher S, Hernandez-Andrade E, Basheer SN, *et al.* Impact of cerebral redistribution on neurodevelopmental outcome in small-for-gestational-age or growth-restricted babies: a systematic review. *Ultrasound Obstet Gynecol* 2015;**46**:398–404. doi:10.1002/uog.14818

6 Khalil A, Morales-Rosellõ J, Townsend R, *et al.* Value of third-trimester cerebroplacental ratio and uterine artery Doppler indices as predictors of stillbirth and perinatal loss. *Ultrasound Obstet Gynecol* 2016;**47**:74–80. doi:10.1002/uog.15729

7 Akolekar R, Syngelaki A, Gallo DM, *et al.* Umbilical and fetal middle cerebral artery Doppler at 35-37 weeks' gestation in the prediction of adverse perinatal outcome. *Ultrasound Obstet Gynecol* 2015;**46**:82–92. doi:10.1002/uog.14842

8 Sovio U, White IR, Dacey A, *et al.* Screening for fetal growth restriction with universal third trimester ultrasonography in nulliparous women in the Pregnancy Outcome Prediction (POP) study: A prospective cohort study. *Lancet* 2015;**386**:2089–97. doi:10.1016/S0140-6736(15)00131-2

9 Gordijn SJ, Beune IM, Thilaganathan B, *et al.* Consensus definition of fetal growth restriction: a Delphi procedure. *Ultrasound Obstet Gynecol* 2016;**48**:333–9. doi:10.1002/uog.15884

For peer review only - http://bmjopen.bmj.com/site/about/guidelines.xhtml

10 Bond DM, Gordon A, Hyett J, *et al.* Planned early delivery versus expectant management of the term suspected compromised baby for improving outcomes. Cochrane Database Syst. Rev. 2015;**2015**. doi:10.1002/14651858.CD009433.pub2

11 Boers KE, Vijgen SMC, Bijlenga D, *et al.* Induction versus expectant monitoring for intrauterine growth restriction at term: Randomised equivalence trial (DIGITAT). *BMJ* 2011;**342**:35. doi:10.1136/bmj.c7087

12 GRIT study group, Thornton JG, Hornbuckle J, *et al.* Infant wellbeing at 2 years of age in the Growth Restriction Intervention Trial (GRIT): Multicentred randomised controlled trial. *Lancet* 2004;**364**:513–20. doi:10.1016/S0140-6736(04)16809-8

13 Lees CC, Marlow N, Van Wassenaer-Leemhuis A, *et al.* 2 year neurodevelopmental and intermediate perinatal outcomes in infants with very preterm fetal growth restriction (TRUFFLE): A randomised trial. *Lancet* 2015;**385**:2162–72. doi:10.1016/S0140-6736(14)62049-3

14 Martins JG, Biggio JR, Abuhamad A. Society for Maternal-Fetal Medicine Consult Series #52: Diagnosis and management of fetal growth restriction. *Am J Obstet Gynecol* 2020;**223**:B2–17. doi:10.1016/j.ajog.2020.05.010

15 Lees CC, Stampalija T, Baschat A, *et al.* ISUOG Practice Guidelines: diagnosis and management of small-for-gestational-age fetus and fetal growth restriction. *Ultrasound Obstet Gynecol* 2020;**56**:298–312. doi:10.1002/uog.22134

16 Mylrea-Foley B, Bhide A, Mullins E, *et al.* Building consensus: thresholds for delivery in TRUFFLE-2 randomized intervention study. Ultrasound Obstet. Gynecol. 2020;**56**:285–7. doi:10.1002/uog.22124

17 Stampalija T, Thornton J, Marlow N, *et al.* Fetal cerebral Doppler changes and outcome in late preterm fetal growth restriction: prospective cohort study. *Ultrasound Obstet Gynecol* 2020;:173–81. doi:10.1002/uog.22125

18 Johnson S, Bountziouka V, Brocklehurst P, *et al.* Standardisation of the Parent Report of Children's Abilities–Revised (PARCA-R): a norm-referenced assessment of cognitive and language development at age 2 years. *Lancet Child Adolesc Heal* 2019;**3**:705–12. doi:10.1016/s2352-4642(19)30189-0

19 Chappell LC, Brocklehurst P, Green ME, *et al.* Planned early delivery or expectant management for late preterm pre-eclampsia (PHOENIX): a randomised controlled trial. *Lancet* 2019;**394**:1181–90. doi:10.1016/S0140-6736(19)31963-4

20 NICE. Developmental follow-up of children and young people born preterm | Quality standards. 2018.

21 Papageorghiou AT, Ohuma EO, Altman DG, *et al.* International standards for fetal growth based on serial ultrasound measurements: The Fetal Growth Longitudinal Study of the INTERGROWTH-21st Project. *Lancet* 2014;**384**:869–79. doi:10.1016/S0140-6736(14)61490-2

22 Kiserud T, Piaggio G, Carroli G, *et al.* *The World Health Organization Fetal Growth Charts : A Multinational Longitudinal Study of Ultrasound Biometric Measurements and Estimated Fetal Weight*. 2017. doi:10.1371/journal.pmed.1002220

For peer review only - http://bmjopen.bmj.com/site/about/guidelines.xhtml

Supplementary Files:

1. Supplementary File 1 - Flowchart
2. Supplementary File 2 - Consent for Observational Data Collection
3. Supplementary File 3 - Consent for Randomisation
4. Supplementary File 4 - PARCA-R Questionnaire
5. Supplementary File 5 - Infant Health Questionnaire
6. Supplementary File 6 - Doppler image scoring sheet
7. Supplementary File 7 - SPIRIT Checklist

For peer review only

TRUFFLE 2 flowchart

32+0 to 36+6 weeks, singleton, non-anomalous pregnancy, age ≥ 18

AND

EFW or AC <10th centile ***OR*** decrease by 50 percentiles

AND

Normal computerised CTG (STV ≥ 4.5msec)

AND

Umbilical Cerebral Ratio > 1.0 at 32+0 - 33+6 weeks
> 0.8 at 34+0 - 36+6 weeks

(UCR must be repeated within 2 – 24 hours to confirm abnormal reading)

AND

No contra-indication to either trial arm

↓

RANDOMISATION (at time of 2nd abnormal UCR)

1:1 central by centre and gestational age (above or below 34 weeks)

↓

IMMEDIATE DELIVERY

Delivery or start of induction of labour within 48 hours of randomisation

To allow for administration of corticosteroids and magnesium sulphate (see guidance)

DELAYED DELIVERY

Twice weekly ultrasound Doppler and cCTG

Delivery mandatory if: cCTG STV < 4.5 msec
OR repeated decelerations
OR if UA EDF is reversed
OR ≥ 34 + 0 if UA EDF absent

↓

PRIMARY OUTCOME – Composite perinatal outcome

↓

SECONDARY OUTCOMES – 2 year neonatal follow-up & maternal outcomes

For peer review only - http://bmjopen.bmj.com/site/about/guidelines.xhtml

ADD LOCAL HOSPITAL HEADER

Information Sheet for Research Participants
TRUFFLE 2 study

Study title:

Perinatal and 2 year neurodevelopmental outcome in late preterm fetal compromise: the TRUFFLE 2 Randomised Trial

Chief Investigator:

Professor Christoph Lees, MD FRCOG

Principal Local Investigator:

You are being invited to take part in a research study, called the TRial of Umbilical and Fetal FLow in Europe (TRUFFLE) 2 Study. This leaflet is to help you decide whether to participate. It tells you why this study is being conducted and what taking part will mean for you. Please take time to read it carefully. Please get in touch with the study team if anything is not clear or if you would like more information. Take your time to decide whether or not you wish to be involved.

If you decide not to take part your future care will not be affected. If you do take part but decide later on that you don't want to after all, you can withdraw at any time – you do not have to give a reason and your care will not be affected.

Who is organising the research?

The TRUFFLE 2 study is being carried out in this hospital by .... and their team of doctors and midwives. It is also taking place in centres across the UK and internationally by members of the TRUFFLE research group. This group is made up of researchers and health professionals who specialise in caring for babies and mothers during pregnancy.

This study is sponsored and organised by Imperial College London, with The Centre for Trials Research, Cardiff University managing the trial on a day to day basis. The trial is being funded by the UK National Institute for Health Research (NIHR).

What is the purpose of the study?

Some babies grow more slowly in the womb than expected. This is called 'fetal growth restriction'. The slow growth can be seen on a scan.

For peer review only - http://bmjopen.bmj.com/site/about/guidelines.xhtml
For peer review only

Poor growth can be a warning sign about the baby's wellbeing. Doctors have many ways to monitor growth restricted babies, but there is no treatment in the womb; the only treatment is to deliver them. TRUFFLE is investigating the best time for this.

At the end of pregnancy (after 37 weeks) delivery is often recommended as there are fewer risks from birth at that point. Very early in pregnancy (before 32 weeks) doctors usually wait as long as possible, because the risks of premature birth are relatively large.

But when a pregnancy is affected by fetal growth restriction between 32 and 36 weeks of pregnancy, the decision about whether to deliver is more difficult. The possible problems of being delivered early must be balanced against the potential problems for the baby from growing slowly whilst in the womb, including stillbirth.

Currently, doctors don't have good information to help them decide about the best time to deliver a baby between 32 and 36 weeks of pregnancy. At the moment many different approaches are being used. This study aims to find answers about the safest time to deliver the baby.

How will the study work?

Pregnant women whose babies are either smaller or growing more slowly than expected between 32 and 36 weeks of pregnancy will be invited to take part in the screening phase of the study. At this point we will ask your permission to collect information about you and your baby.

Baseline Questionnaire

If you agree to take part in the screening phase of the study, we will ask you for your email address so we can email you a questionnaire to complete. This questionnaire will ask you questions about your ethnicity, job status and drinking and smoking habits.

Apart from this, your care will not change during the screening phase. You will have regular scans and baby heart rate monitoring as normal for small babies. One of the scan tests involves measuring the blood flow through the umbilical cord, and another the blood flow to the baby's brain.

If the blood flow is redirected to the baby's brain, this may be an early warning sign of problems and can be measured by a Doppler ultrasound. The idea is that if the placenta is not working well the baby responds by diverting blood to the most vital organ, the brain. However, many babies have this redistribution pattern remain otherwise perfectly healthy. We can test this by looking at the heart rate pattern. This normally varies moment by moment. You can see this as the line on the monitor being wiggly, rather than flat. The heart rate monitor also calculates a number to measure this pattern objectively. This number is known as short term variation.

If your baby's blood flow is redirected to their brain, but they also have a normal heart rate pattern, some experts would recommend delivery. Others would prefer to wait for the heart rate pattern to change. If you were not taking part in the TRUFFLE 2 trial your treatment would depend on which hospital and/or expert was treating you.

At this point we will invite you to join the main TRUFFLE 2 trial.
If you agree to participate at this stage you will be randomly allocated by a computer to receive one of two methods of treatment.
The two groups are:

For peer review only - http://bmjopen.bmj.com/site/about/guidelines.xhtml

- To begin delivery within two days of the Doppler ultrasound blood flow test showing blood flow being redirected to the baby's brain, but before the heart rate pattern changes.
- To wait to deliver until the baby's heart rate pattern changes. This will typically involve twice weekly appointments for baby heart rate monitoring. Your doctor will recommend the exact frequency of this monitoring.

The way in which you give birth, vaginal or caesarean, will not be altered by participation in the trial. That will be left up to your preference and your doctor's recommendation. Your doctors may advise drugs that would be given routinely to improve baby's health when they are born early. These might include steroid injections for lung development and magnesium sulphate infusion for brain protection. Again, these will not be altered by participation in the trial. Your doctors will tell you what drug regime is normally used in your hospital.

We will assess how your baby is when she or he is first born, and then check their development up to two years of age by emailing you questionnaires to complete.

General Health Questionnaire
A general health questionnaire may be emailed to you 6, 12, 18 and 24 months after your baby is born. This questionnaire will ask you details of any hospital admissions for your baby in the last 6 months.

PARCA-R Questionnaire
The PARCA-R questionnaire may be emailed to you 2 years after your baby is born. This questionnaire is made up of two parts to look at your child's development. The questionnaire will be completed by you and will take 15 minutes.

All these questionnaires will be emailed to the email address you provided to the TRUFFLE 2 trials team. The email will come from no-reply@CastorEDC.com, please make sure you check your inbox around the times described above and let the research midwife know if you change your email address. Once completed the questionnaires will be sent back to the TRUFFLE 2 trials team.

We will also use anonymous information collected in the study to determine if there is a difference in the healthcare costs from the different groups in the TRUFFLE 2 study.

Do I have to take part?

No. It is your choice. If you are willing to be part of the study, you will be asked to complete a consent form. If you prefer not to take part, tell your doctor and we will not ask you again. If you decide to take part you are still free to withdraw at any time and without giving a reason. This will not affect the standard of care you receive in the future.

What are the possible disadvantages and risks of taking part?

The timing of your baby's birth will be based on which treatment group you are allocated to. The risks of being born too early are mainly of breathing problems for the baby and a very small risk of bleeding in the brain related to prematurity. The risks of waiting are that the baby's condition may deteriorate rapidly such that he or she gets seriously short of oxygen. All of these risks are very small. Neither ultrasound nor baby heart rate monitoring will cause harm to your baby directly.

What are the possible benefits of taking part?

For peer review only - http://bmjopen.bmj.com/site/about/guidelines.xhtml

There are no direct benefits to you from taking part in this study. There is some evidence that people who participate in medical research studies have generally better outcomes than those who don't. We hope that many women and their children in the future will benefit from your participation and the information we gain from this study.

What if a problem is detected?

If any unexpected problems are detected over the course of the study, your doctor will treat you as they think best, whatever trial group you are in. If, for example, you were allocated to the "wait for fetal heart rate changes before birth" group and a new problem, such as bleeding behind the placenta, developed such that delivery became the safest option, your doctor would deliver you. In such a scenario we would still wish to follow up you and your baby.

What happens if I withdraw?

If you decide to withdraw from the study, there are two options.

The first option is it withdraw consent to follow the treatment you were randomised to. This request will be respected and you can still give permission to be followed up by the study team and your data still analysed. You can decide to withdraw from the timing of delivery part of the study at any time without explanation. If you do so, your future care will not be affected by your decision.

The second option is to withdraw from the randomised treatment but also from any follow up data being collected as well. You are free to withdraw from follow up, however if many participants withdraw from follow up it could impact our results, we would therefore strongly prefer that you do not do this. If follow up becomes difficult, please discuss this with the study doctors or midwives. We will usually be able to reduce the burden, perhaps by limiting our contact to your GP rather than contacting you directly. Data that has been collected with your permission before you withdraw from follow up will be included in the study analysis.

Will my taking part in this study be kept confidential?

Imperial College London is the sponsor for this study based in the United Kingdom. We will be using information from you and your medical records in order to undertake this study and will act as the data controller for this study. This means that we are responsible for looking after your information and using it properly. Imperial College London will keep identifiable information about you for 10 years.

Further information on Imperial College London's retention periods may be found at https://www.imperial.ac.uk/media/imperial-college/administration-and-support-services/records-and-archives/public/RetentionSchedule.pdf.

Your rights to access, change or move your information are limited, as we need to manage your information in specific ways in order for the research to be reliable and accurate. If you withdraw from the study, we will keep the information about you that we have already obtained. To safeguard your rights, we will use the minimum personally-identifiable information possible. You can find out more about how we use your information [Dr Christoph Lees Email contact: c.lees@imperial.ac.uk].

LEGAL BASIS

For peer review only - http://bmjopen.bmj.com/site/about/guidelines.xhtml

As a university we use personally-identifiable information to conduct research to improve health, care and services. As a publicly-funded organisation, we have to ensure that it is in the public interest when we use personally-identifiable information from people who have agreed to take part in research. This means that when you agree to take part in a research study, we will use your data in the ways needed to conduct and analyse the research study.

Health and care research should serve the public interest, which means that we have to demonstrate that our research serves the interests of society as a whole. We do this by following the UK Policy Framework for Health and Social Care Research.

INTERNATIONAL TRANSFERS

There may be a requirement to transfer information to countries outside the European Economic Area (for example, to a research partner). Where this information contains your personal data, Imperial College London will ensure that it is transferred in accordance with data protection legislation. If the data is transferred to a country which is not subject to a European Commission (EC) adequacy decision in respect of its data protection standards, Imperial College London will enter into a data sharing agreement with the recipient organisation that incorporates EC approved standard contractual clauses that safeguard how your personal data is processed.

CONTACT US

If you wish to raise a complaint on how we have handled your personal data or if you want to find out more about how we use your information, please contact Imperial College London's Data Protection Officer via email at dpo@imperial.ac.uk, via telephone on 020 7594 3502 and via post at Imperial College London, Data Protection Officer, Faculty Building Level 4, London SW7 2AZ.

If you are not satisfied with our response or believe we are processing your personal data in a way that is not lawful you can complain to the Information Commissioner's Office (ICO). The ICO does recommend that you seek to resolve matters with the data controller (us) first before involving the regulator.

Imperial College London will collect information about you for this research study from Imperial College Healthcare Trust. This information will include your hospital number, contact details and health information, which is regarded as a special category of information. We will use this information to conduct this study and contact you with questionnaires.

How we will use and store your information

The TRUFFLE-2 researchers will use and store information about you and your baby for the purpose of the research. This will include contacting you, your hospital doctors and your family doctor to follow you both up. They will keep your information secure and confidential in accord with European data protection rules. Certain authorised individuals may also look at your medical and research records to check the accuracy of the research study.

Potential use of study data for future research

When you agree to take part anonymous information about your health and care may be provided to other authorised researchers. This use of your information without your explicit permission is strictly regulated and will not identify you.

For peer review only - http://bmjopen.bmj.com/site/about/guidelines.xhtml

We will also ask your permission to contact you in future to participate in future research studies where your identifiable information would be used. Your participation in such research will only be with your consent.

Who has reviewed the study?

This study was given a favourable ethical opinion for conduct in the NHS by the **London- Riverside** Regional Ethics Committee NHS Health Research Authority.

The IRAS reference number is 266400.

What if something goes wrong?

Imperial College London holds insurance policies which apply to this study. If you experience harm or injury as a result of taking part in this study, you will be eligible to claim compensation without having to prove that Imperial College is at fault. This does not affect your legal rights to seek compensation. If you are harmed due to someone's negligence, then you may have grounds for a legal action.

Regardless of this, if you wish to complain, or have any concerns about any aspect of the way you have been treated during the course of this study then you should immediately inform the Investigator (Dr Christoph Lees Email contact: c.lees@imperial.ac.uk).

The normal National Health Service complaints mechanisms are also available to you. If you are still not satisfied with the response, you may contact the Imperial AHSC Joint Research Compliance Office.

Contact for further information:

For more information you can phone:

Research Fellow and Midwife on 02033137316, or Professor Christoph Lees on 02075942104

Or write to either:

TRUFFLE 2 Investigators, Centre for Fetal Care, Queen Charlotte's & Chelsea Hospital, Du Cane Road, W12 0HS, London
Email: Imperial.TRUFFLEstudy@nhs.net

PALS:

If you have any concerns or wish to complain the details of your local Patient Advice and Liaison Service (PALS) are available on your local hospital's website or on www.nhs.uk.

For peer review only

For peer review only - http://bmjopen.bmj.com/site/about/guidelines.xhtml

ADD LOCAL HOSPITAL HEADER

INFORMED CONSENT FORM for DATA COLLECTION

Full Title of Project: **Perinatal and 2 year neurodevelopmental outcome in late preterm fetal compromise: the TRUFFLE 2 Randomised Trial**

Chief Investigator: **Professor Christoph Lees**

Participant number: ☐☐ __ ☐☐☐ __ ☐☐☐

This consent form is for patients who are PRE-ELIGIBLE, who have a small baby with a NORMAL cerebral Doppler.

	**Please initial box**
1. I confirm that I have read and understand the patient information sheet dated ……/……../………., version ……. for the above trial. I have had the opportunity to ask questions and have had these answered satisfactorily.	☐
2. I understand that my participation is voluntary and I am free to withdraw at any time, without giving any reason and without my medical care or legal rights being affected.	☐
3. I understand that sections of my and my baby's medical notes may be looked at by responsible individuals from Imperial College London, from the NHS Trust or from regulatory authorities where it is relevant to my taking part in this research.	☐
4. I give permission for these individuals to access my and my baby's records that are relevant to this research.	☐
5. **Optional:** I give consent for information collected about me and my baby to be used to support other ethically approved research in the future, including those outside of the EEA.	☐
6. **Optional:** I consent to being contacted to potentially taking part in other research studies.	☐
7. I consent for information from my records to be used as part of the TRUFFLE 2 trial.	☐
8. I understand that this form is NOT consent for randomisation.	☐

© Imperial College London
IRAS ID: 266400

Imperial College London
For peer review only - http://bmjopen.bmj.com/site/about/guidelines.xhtml

______________________	_______________	_______________
Name of Subject	Signature	Date
______________________	_______________	_______________
Name of Person taking consent	Signature	Date
______________________	_______________	_______________
Principal Investigator	Signature	Date

For peer review only

1 copy for subject; 1 copy for Principal Investigator; 1copy to be kept with hospital notes

© Imperial College London

For peer review only - http://bmjopen.bmj.com/site/about/guidelines.xhtml

ADD LOCAL HOSPITAL HEADER

INFORMED CONSENT FORM for RANDOMISATION

Full Title of Project: **Perinatal and 2 year neurodevelopmental outcome in late preterm fetal compromise: the TRUFFLE 2 Randomised Trial**

Chief Investigator: **Professor Christoph Lees**

Participant number: ☐☐ __ ☐☐☐ __ ☐☐☐

This consent form is for patients who are ELIGIBLE FOR RANDOMISATION. This means they have a small baby (EFW or AC <10th or fallen 50 centiles) AND an abnormal UCR (repeated to confirm abnormality within 2-24 hours).

Please initial box

Part A

1. I confirm that I have read and understand the patient information sheet dated ... / ... /... *Version* .... for the above trial. I have had the opportunity to ask questions and have had these answered satisfactorily. ☐

2. I understand that my participation is voluntary and I am free to withdraw at any time, without giving any reason and without my medical care or legal rights being affected. ☐

3. I understand that sections of any of my and my baby's medical notes may be looked at by responsible individuals from Imperial College London, from the NHS Trust or from regulatory authorities where it is relevant to my taking part in this research. ☐

4. I give permission for these individuals to access my and my baby's records that are relevant to this research. ☐

5. I understand that my family doctor will be informed of my participation in the trial. ☐

6. **Optional:** I give consent for information collected about me and my baby to be used to support other ethically approved research in the future, including those outside of the EEA. ☐

7. **Optional:** I consent to being contacted to potentially taking part in other research studies. ☐

Part B

8. I consent to be randomised to either immediate or delayed delivery for this trial. ☐

Name of Subject	Signature	Date
Name of Person taking consent	Signature	Date
Principal Investigator	Signature	Date

1 copy for subject; 1 copy for Principal Investigator; 1copy to be kept with hospital notes

For peer review only

© Imperial College London
Imperial College London
IRAS ID:

Parent Report of Children's Abilities – Revised (PARCA-R Questionnaire)

For peer review only

Your child's health and development at 2 years

In this form we ask you to answer some questions about your child and your family. This information is used to work out how your child is developing now that she/he is just over two years old. **Please complete all the questions as accurately as possible.**

If you need any help completing the questionnaire, or have any queries about the questions, please do not hesitate to ask the doctor about these at your appointment.

Permissions:
The PARCA questionnaire was adapted for use with infants born preterm (Johnson et al., Dev Med Child Neurol 2004, 46;389-397) with permission from Saudino, Dale, Oliver, Petrill, Richardson, Rutter, Simonoff, Stevenson & Plomin (1998). The language measures included in this questionnaire are used with permission from the MacArthur-Bates CDI Advisory Board, Chair: Larry Fenson (2016).

For further information about the English version of the PARCA-R questionnaire, please **email**: **parca-r@leicester.ac.uk**

Your child's play

As a parent, you will have a good idea of what your child can and can't do. Listed below are a number of activities. Please indicate whether or not your child can do the activity. That is, if you have seen your child do the activity (or something similar) then tick the box under "YES". If you know that your child would not be able to do it, then tick the box under "NO". If you are not sure whether or not your child can do it, then tick the box under "DON'T KNOW". Please answer every question.

Please keep in mind that these questions are for children ranging from 18 months to 4 years. Some activities may be easy for your child, others may be difficult. Most children of your child's age will not be able to do some of the activities.

		YES	NO	DON'T KNOW
1	**Does your child copy things you do such as cuddling a teddy?** (Try it out if not sure by cuddling a teddy and then giving it to your child. Say: Now you cuddle teddy)	☐	☐	☐
2	**When you hide a toy in full view of your child, will s/he look for it and find it?** (Try this out by covering a small toy with a cloth or a cup and seeing if s/he uncovers the toy)	☐	☐	☐
3	**Can your child put a simple piece, such as a square or an animal, into the correct place in a puzzle board?**	☐	☐	☐
4	**Some toys have several holes or openings with different shapes, such as a circle, triangle, and star. Could your child put the shapes into the right openings?**	☐	☐	☐
5	**Can your child stack two small blocks or toys on top of each other?**	☐	☐	☐
6	**Can your child put together, by him/herself, a puzzle or something similar where the pieces fit together?**	☐	☐	☐
7	**If so, can s/he do this for a puzzle with ten or more pieces?**	☐	☐	☐
8	**Can your child mark on a piece of paper using the tip of a crayon, pencil, or chalk?**	☐	☐	☐
9	**Can your child draw a more or less straight line on paper?**	☐	☐	☐
10	**Does your child turn, or try to turn, pages of a book one at a time?**	☐	☐	☐
11	**Does your child ever pretend that one object, such as a block, is another object, such as a car or a telephone?**	☐	☐	☐

For further information about the English version of the PARCA-R questionnaire, please **email**:

parca-r@leicester.ac.uk

For peer review only - http://bmjopen.bmj.com/site/about/guidelines.xhtml

		YES	NO	DON'T KNOW
12	Can your child stack three small blocks or toys on top of each other by him/herself?	☐	☐	☐
13	Does your child ever pretend to do things? For example, riding a horse or making a cup of tea?	☐	☐	☐
14	Can your child push a car along the floor with the wheels on the floor?	☐	☐	☐
15	Does your child look with interest at pictures in a book?	☐	☐	☐
16	Does your child point to pictures in a book?	☐	☐	☐
17	Does your child try to copy things you do, such as stirring with a spoon in a cup?	☐	☐	☐
18	Can your child stack seven small blocks or toys on top of each other by him/herself?	☐	☐	☐
19	Does your child point or show where people or objects are when you ask: "Where is the light?" "Where is Daddy?" or "Where is Teddy?"	☐	☐	☐
20	Does your child ever pretend that two dolls are playing together, or are talking to each other, or one is feeding the other?	☐	☐	☐
21	Does your child ever play pretend games with another child, pretending to be someone else, such as a mummy, daddy, policeman, or nurse?	☐	☐	☐
22	Does your child ever play any game with another child that involves taking turns?	☐	☐	☐
23	Does your child ever copy some action shortly (within a few minutes) after s/he has seen it?	☐	☐	☐
24	Can your child fetch something, such as a toy, from another room by him/herself when you ask?	☐	☐	☐
25	Does your child know where some things belong, such as, that his/her toys belong in a box?	☐	☐	☐
26	Does your child ever save or put to one side a biscuit (or snack) for later, on his/her own?	☐	☐	☐
27	Have you ever seen your child get together three or more toys before beginning to play with them?	☐	☐	☐
28	Have you ever seen your child sort things (blocks, other toys) into groups or piles that go together on his/her own?	☐	☐	☐

For further information about the English version of the PARCA-R questionnaire, please **email**:

parca-r@leicester.ac.uk

For peer review only - http://bmjopen.bmj.com/site/about/guidelines.xhtml

		YES	NO	DON'T KNOW
29	**If your child wants something out of reach, does s/he go and find a chair or box to stand on?**	☐	☐	☐
30	**When your child uses or plays with a telephone, does s/he speak into the mouthpiece not the earpiece?**	☐	☐	☐
31	**When your child drinks from a cup, is s/he careful about putting it down, trying not to spill it?**	☐	☐	☐
32	**Does your child try to turn doorknobs, twist tops, or screw lids on or off jars?**	☐	☐	☐
33	**Does your child recognise him/her self when looking in the mirror?**	☐	☐	☐
34	**Does your child ever use his/her index (first) finger to point to show an interest in something?**	☐	☐	☐

For peer review only

For further information about the English version of the PARCA-R questionnaire, please **email**:

parca-r@leicester.ac.uk

For peer review only - http://bmjopen.bmj.com/site/about/guidelines.xhtml

What your child can say

1. Children understand many more words than they can say. Here, we are only interested in the words your child SAYS. Please tick all the words you have heard your child say. If your child uses a different pronunciation of a word – e.g., "tend" for pretend, or "duce" for juice – tick it anyway. Please keep in mind that this is only a sample of words; your child may know many other words not on this list.

☐ Baa baa	☐ Cream cracker	☐ Bed	☐ Carry	☐ Last
☐ Meow	☐ Juice	☐ Bedroom	☐ Chase	☐ Tiny
☐ Ouch/ow	☐ Meat	☐ Settee/sofa	☐ Pour	☐ Wet
☐ Uh-oh/oh dear	☐ Milk	☐ Oven/cooker	☐ Finish	☐ After
☐ Woof woof	☐ Peas	☐ Stairs	☐ Fit	☐ Day
☐ Bear	☐ Hat	☐ Flag	☐ Hug/cuddle	☐ Tonight
☐ Bird	☐ Necklace	☐ Rain	☐ Listen	☐ Our
☐ Cat	☐ Shoe	☐ Star	☐ Like	☐ Them
☐ Dog	☐ Sock	☐ Swing	☐ Pretend	☐ This
☐ Duck	☐ Chin	☐ School	☐ Rip/tear	☐ Us
☐ Horse	☐ Ear	☐ Sky	☐ Shake	☐ Where
☐ Aeroplane	☐ Hand	☐ Zoo	☐ Taste	☐ Beside
☐ Boat	☐ Leg	☐ Friend	☐ Gentle	☐ Down
☐ Car	☐ Pillow	☐ Mummy/mum	☐ Think	☐ Under
☐ Ball	☐ Comb	☐ Person	☐ Wish	☐ All
☐ Book	☐ Lamp/torch	☐ Bye/byebye	☐ All gone	☐ Much
☐ Game	☐ Plate	☐ Hi/hello	☐ Cold	☐ Could
☐ Sandwich	☐ Rubbish	☐ No	☐ Fast	☐ Need to
☐ Fish	☐ Tray	☐ Shopping	☐ Happy	☐ Would
☐ Sauce	☐ Towel	☐ Thank you	☐ Hot	☐ If

For peer review only

For further information about the English version of the PARCA-R questionnaire, please **email**:

parca-r@leicester.ac.uk

How your child uses words

2. We would like to know how your child uses the words s/he can say. Please tick one box for each question below to tell us whether your child uses words like this often, sometimes, or not yet.

Please keep in mind that these questions are for children up to 4 years of age. Many children of your child's age will not be able say some of the words or sentences below.

		OFTEN	SOMETIMES	NOT YET
**A1**	Does your child ever talk about past events or people who are not present? For example, a child who saw a carnival last week might later say 'carnival', 'clown', or 'band'.	☐	☐	☐
**A2**	Does your child ever talk about something that is going to happen in the future? E.g. saying 'choo-choo' or 'bus' before you leave the house on a trip, or saying 'swing' when you are going to the park?	☐	☐	☐
**A3**	Does your child ever talk about objects that are not present? For example, asking about a missing toy not in the room, or asking about someone not present?	☐	☐	☐
**A4**	Does your child understand if you ask for something that is not in the room? For example, would s/he go to the bedroom to get a teddy bear when you say 'Where's the bear?'	☐	☐	☐
**A5**	Does your child know who things belong to? For example, a child might point to mummy's shoe and say 'Mummy'.	☐	☐	☐
**A6**	Has your child started to put together words yet, such as 'Daddy gone' or 'Doggie bite'?	☐	☐	☐

If you answered "Sometimes" or "Often" to question A6, please answer all the questions on the next page.

For further information about the English version of the PARCA-R questionnaire, please **email**:

parca-r@leicester.ac.uk

For peer review only - http://bmjopen.bmj.com/site/about/guidelines.xhtml

3. For EACH PAIR of sentences below – A and B – please tick the one that sounds MOST like the way your child talks at the moment, even if s/he would not say that EXACT sentence. If your child is saying sentences even more complicated than the two examples provided, tick B.

	**Talking about something happening right now**			**Talking about something that already happened**				
**A7**			**A8**			**A9**		
A	☐	I make tower	A	☐	Daddy pick me up	A	☐	That my truck
B	☐	I making tower	B	☐	Daddy picked me up	B	☐	That's my truck
**A10**			**A11**			**A12**		
A	☐	Baby crying	A	☐	There a doggie	A	☐	Coffee hot
B	☐	Baby is crying	B	☐	There's a doggie	B	☐	That coffee hot
**A13**			**A14**			**A15**		
A	☐	I no do it	A	☐	I like read stories	A	☐	Biscuit mummy
B	☐	I can't do it	B	☐	I like to read stories	B	☐	Biscuit for mummy
**A16**			**A17**			**A18**		
A	☐	Don't read book	A	☐	Baby want eat	A	☐	Look at me
B	☐	Don't want you read that book	B	☐	Baby want to eat	B	☐	Look at me dancing

For peer review only

Thank you very much for your time

For further information about the English version of the PARCA-R questionnaire, please **email**:

parca-r@leicester.ac.uk

Survey 'GB_InfantHealthSurvey'

GB_InfantHealthSurvey - InfantGrowth

Number	Question	Answers
	**Could you enter below the last weight and height of your child and the date when it was measured. Please use the measurements from your last visit to a child health clinic, or take the measurements yourself.**	
1.1	Date of measurement	___ ___ ___ *(dd-mm-yyyy)*
1.2	Height (cm)	___ cm
1.3	Weight (Kg)	___ kg

GB_InfantHealthSurvey - InfantInpatientCare

Number	Question	Answers
	**The following questions ask about your child's use of hospital services for the LAST SIX MONTHS only. Please answer all questions as accurately as possible.**	
2.1	Has your child been admitted to hospital in the last six months?	○ Yes ◉ No
2.1.1	***If 'Has your child been admitted to hospital in the last six months?' is equal to 'Yes' answer this question:*** In which month was your child admitted in hospital?	○ January<br>○ February<br>○ March<br>○ April<br>○ May<br>○ June<br>○ July<br>○ August<br>○ September<br>○ October<br>○ November<br>○ December

2.1.2	***If 'Has your child been admitted to hospital in the last six months?' is equal to 'Yes' answer this question:***<br>In which year was your child admitted in hospital?	○ 2020<br>○ 2021<br>○ 2022<br>○ 2023<br>○ 2024<br>○ 2025<br>○ 2026
2.1.3	***If 'Has your child been admitted to hospital in the last six months?' is equal to 'Yes' answer this question:***<br>Was the admission planned or an emergency	○ Planned<br>○ Emergency
2.1.4	***If 'Has your child been admitted to hospital in the last six months?' is equal to 'Yes' answer this question:***<br>What was the reason for the admission?	
2.1.5	***If 'Has your child been admitted to hospital in the last six months?' is equal to 'Yes' answer this question:***<br>Did your child stay overnight in the hospital?	○ Yes ○ No
2.1.5.1	***If 'Did your child stay overnight in the hospital?' is equal to 'Yes' answer this question:***<br>For how many nights did your child stay in hospital?	
2.1.6	***If 'Has your child been admitted to hospital in the last six months?' is equal to 'Yes' answer this question:***<br>Did your child receive intensive care?	○ Yes ○ No
2.1.6.1	***If 'Did your child receive intensive care?' is equal to 'Yes' answer this question:***<br>For how many nights did your child stay in the intensive care?	
2.2	Check Admission	
2.2.1	***If 'Check Admission' is equal to '1' answer this question:***<br>Has your child been admitted to hospital for a second time in the last six months?	○ Yes ○ No
	**---_SECOND ADMISSION_---**	

GB_InfantHealthSurvey - InfantOutpatientCare

Number	Question	Answers
	**The following questions ask if your child attended a hospital outpatient department/clinic for the LAST SIX MONTHS only. Please answer all questions as accurately as possible.**	
3.1	Has your child attended a hospital outpatient department/clinic in the last six months	○ Yes ◉ No
3.1.1	***If 'Has your child attended a hospital outpatient department/clinic in the last six months' is equal to 'Yes' answer this question:***<br>Which type of clinic did your child visit during the last six months?	○ Accident or Emergency<br>○ Paediatric / Neonatal Follow-up<br>○ Orthopaedic<br>○ Audiology / Hearing<br>○ Eye / Vision<br>○ General Medicine<br>○ Dermatology<br>○ Other
3.1.1.1	***If 'Which type of clinic did your child visit during the last six months?' is equal to 'Other' answer this question:***<br>Which specialty of outpatient department / clinic did your child visit during the last six months?	
3.1.2	***If 'Has your child attended a hospital outpatient department/clinic in the last six months' is equal to 'Yes' answer this question:***<br>What was the reason for this visit?	
3.1.3	***If 'Has your child attended a hospital outpatient department/clinic in the last six months' is equal to 'Yes' answer this question:***<br>How many times did your child visit this clinic during the past half year?	
3.2	CheckOPD	
3.2.1	***If 'CheckOPD' is equal to '1' answer this question:***<br>Has your child attended a second hospital outpatient department/clinic in the last six months	○ Yes ○ No
	**---_SECOND VISIT_---**	

TRUFFLE 2

Imperial College London

Doppler Image Scoring

For Umbilical Artery:

PULSEWAVE IMAGE (/6)	1 point	0 point
Magnification	Doppler display occupies 50% or more of the image	Doppler display occupies <50% of the image
Angle of insonation	<30 degrees	30 degrees or more
Sweep speed	Doppler spectrum has 4-10 waveforms	Doppler spectrum has < 4 (3 or less) or > 10 waveforms (11 or more)
Sample gate	Large enough to include 3/4th of the vessel diameter	Smaller than 3/4th of the vessel diameter
Appropriate PRF	The waveform fits at least 75% of the pulse wave Doppler scale or PRF (pulse repetition frequency)	The waveform fits < 75% of the pulse wave Doppler scale or PRF
Image quality	Uniform arterial waveforms, no aliasing or background artefacts or fetal breathing movements	Variable arterial waveforms/ aliasing/background artefacts/fetal breathing movements

For Middle Cerebral Arterial: (as above)

PULSEWAVE IMAGE (/6)	1 point	0 point
Magnification	Doppler display occupies 50% or more of the image	Doppler display occupies <50% of the image
Angle of insonation	<30 degrees	30 degrees or more
Sweep speed	Doppler spectrum has 4-10 waveforms	Doppler spectrum has < 4 (3 or less) or > 10 waveforms (11 or more)
Sample gate	Large enough to include 3/4th of the vessel diameter	Smaller than 3/4th of the vessel diameter
Appropriate PRF	The waveform fits at least 75% of the pulse wave Doppler scale or PRF	The waveform fits < 75% the pulse wave Doppler scale or PRF
Image quality	Uniform arterial waveforms, no aliasing or background artefacts or fetal breathing movements	Variable arterial waveforms/ aliasing/background artefacts/fetal breathing movements

For peer review only

SPIRIT

STANDARD PROTOCOL ITEMS: RECOMMENDATIONS FOR INTERVENTIONAL TRIALS

SPIRIT 2013 Checklist: Recommended items to address in a clinical trial protocol and related documents

Section/item	Item No	Description	Addressed on page number
**Administrative information**			
Title	1	Descriptive title identifying the study design, population, interventions, and, if applicable, trial acronym	1
Trial registration	2a	Trial identifier and registry name. If not yet registered, name of intended registry	4
	2b	All items from the World Health Organization Trial Registration Data Set	4
Protocol version	3	Date and version identifier	*see note
Funding	4	Sources and types of financial, material, and other support	4, 14
Roles and responsibilities	5a	Names, affiliations, and roles of protocol contributors	1-3, 14
	5b	Name and contact information for the trial sponsor	4
	5c	Role of study sponsor and funders, if any, in study design; collection, management, analysis, and interpretation of data; writing of the report; and the decision to submit the report for publication, including whether they will have ultimate authority over any of these activities	14
	5d	Composition, roles, and responsibilities of the coordinating centre, steering committee, endpoint adjudication committee, data management team, and other individuals or groups overseeing the trial, if applicable (see Item 21a for data monitoring committee)	13

For peer review only

Introduction

Background and rationale	6a	Description of research question and justification for undertaking the trial, including summary of relevant studies (published and unpublished) examining benefits and harms for each intervention	6-7
	6b	Explanation for choice of comparators	6-7
Objectives	7	Specific objectives or hypotheses	7
Trial design	8	Description of trial design including type of trial (eg, parallel group, crossover, factorial, single group), allocation ratio, and framework (eg, superiority, equivalence, noninferiority, exploratory)	7

Methods: Participants, interventions, and outcomes

Study setting	9	Description of study settings (eg, community clinic, academic hospital) and list of countries where data will be collected. Reference to where list of study sites can be obtained	7
Eligibility criteria	10	Inclusion and exclusion criteria for participants. If applicable, eligibility criteria for study centres and individuals who will perform the interventions (eg, surgeons, psychotherapists)	7-8, 11
Interventions	11a	Interventions for each group with sufficient detail to allow replication, including how and when they will be administered	8
	11b	Criteria for discontinuing or modifying allocated interventions for a given trial participant (eg, drug dose change in response to harms, participant request, or improving/worsening disease)	N/A
	11c	Strategies to improve adherence to intervention protocols, and any procedures for monitoring adherence (eg, drug tablet return, laboratory tests)	N/A
	11d	Relevant concomitant care and interventions that are permitted or prohibited during the trial	7-8
Outcomes	12	Primary, secondary, and other outcomes, including the specific measurement variable (eg, systolic blood pressure), analysis metric (eg, change from baseline, final value, time to event), method of aggregation (eg, median, proportion), and time point for each outcome. Explanation of the clinical relevance of chosen efficacy and harm outcomes is strongly recommended	9-10
Participant timeline	13	Time schedule of enrolment, interventions (including any run-ins and washouts), assessments, and visits for participants. A schematic diagram is highly recommended (see Figure)	8, 11, Supp File 1

For peer review only - http://bmjopen.bmj.com/site/about/guidelines.xhtml

Sample size	14	Estimated number of participants needed to achieve study objectives and how it was determined, including clinical and statistical assumptions supporting any sample size calculations	11-12
Recruitment	15	Strategies for achieving adequate participant enrolment to reach target sample size	7, 13
**Methods: Assignment of interventions (for controlled trials)**			
Allocation:			
Sequence generation	16a	Method of generating the allocation sequence (eg, computer-generated random numbers), and list of any factors for stratification. To reduce predictability of a random sequence, details of any planned restriction (eg, blocking) should be provided in a separate document that is unavailable to those who enrol participants or assign interventions	8
Allocation concealment mechanism	16b	Mechanism of implementing the allocation sequence (eg, central telephone; sequentially numbered, opaque, sealed envelopes), describing any steps to conceal the sequence until interventions are assigned	8
Implementation	16c	Who will generate the allocation sequence, who will enrol participants, and who will assign participants to interventions	8-11
Blinding (masking)	17a	Who will be blinded after assignment to interventions (eg, trial participants, care providers, outcome assessors, data analysts), and how	N/A
	17b	If blinded, circumstances under which unblinding is permissible, and procedure for revealing a participant's allocated intervention during the trial	N/A
**Methods: Data collection, management, and analysis**			
Data collection methods	18a	Plans for assessment and collection of outcome, baseline, and other trial data, including any related processes to promote data quality (eg, duplicate measurements, training of assessors) and a description of study instruments (eg, questionnaires, laboratory tests) along with their reliability and validity, if known. Reference to where data collection forms can be found, if not in the protocol	9-11
	18b	Plans to promote participant retention and complete follow-up, including list of any outcome data to be collected for participants who discontinue or deviate from intervention protocols	11-12

Data management	19	Plans for data entry, coding, security, and storage, including any related processes to promote data quality (eg, double data entry; range checks for data values). Reference to where details of data management procedures can be found, if not in the protocol	13
Statistical methods	20a	Statistical methods for analysing primary and secondary outcomes. Reference to where other details of the statistical analysis plan can be found, if not in the protocol	11-12
	20b	Methods for any additional analyses (eg, subgroup and adjusted analyses)	12
	20c	Definition of analysis population relating to protocol non-adherence (eg, as randomised analysis), and any statistical methods to handle missing data (eg, multiple imputation)	12
**Methods: Monitoring**			
Data monitoring	21a	Composition of data monitoring committee (DMC); summary of its role and reporting structure; statement of whether it is independent from the sponsor and competing interests; and reference to where further details about its charter can be found, if not in the protocol. Alternatively, an explanation of why a DMC is not needed	13
	21b	Description of any interim analyses and stopping guidelines, including who will have access to these interim results and make the final decision to terminate the trial	13
Harms	22	Plans for collecting, assessing, reporting, and managing solicited and spontaneously reported adverse events and other unintended effects of trial interventions or trial conduct	13
Auditing	23	Frequency and procedures for auditing trial conduct, if any, and whether the process will be independent from investigators and the sponsor	13
**Ethics and dissemination**			
Research ethics approval	24	Plans for seeking research ethics committee/institutional review board (REC/IRB) approval	14
Protocol amendments	25	Plans for communicating important protocol modifications (eg, changes to eligibility criteria, outcomes, analyses) to relevant parties (eg, investigators, REC/IRBs, trial participants, trial registries, journals, regulators)	14

Consent or assent	26a	Who will obtain informed consent or assent from potential trial participants or authorised surrogates, and how (see Item 32)	8, Supp Files 2+3
	26b	Additional consent provisions for collection and use of participant data and biological specimens in ancillary studies, if applicable	N/A
Confidentiality	27	How personal information about potential and enrolled participants will be collected, shared, and maintained in order to protect confidentiality before, during, and after the trial	13
Declaration of interests	28	Financial and other competing interests for principal investigators for the overall trial and each study site	*see note
Access to data	29	Statement of who will have access to the final trial dataset, and disclosure of contractual agreements that limit such access for investigators	*see note
Ancillary and post-trial care	30	Provisions, if any, for ancillary and post-trial care, and for compensation to those who suffer harm from trial participation	*see note
Dissemination policy	31a	Plans for investigators and sponsor to communicate trial results to participants, healthcare professionals, the public, and other relevant groups (eg, via publication, reporting in results databases, or other data sharing arrangements), including any publication restrictions	14
	31b	Authorship eligibility guidelines and any intended use of professional writers	14
	31c	Plans, if any, for granting public access to the full protocol, participant-level dataset, and statistical code	13-14
**Appendices**			
Informed consent materials	32	Model consent form and other related documentation given to participants and authorised surrogates	Supp Files 2+3
Biological specimens	33	Plans for collection, laboratory evaluation, and storage of biological specimens for genetic or molecular analysis in the current trial and for future use in ancillary studies, if applicable	N/A

*Live study documents (such as protocol, consent forms, etc) are continuously reviewed and updated as the trial is ongoing, these all contain version numbers and dates, and each site must demonstrate that they are using the up to date and current version. Contracts between Imperial and each participating site contain information regarding confidentiality, publication, data protection, indemnity, etc.

For peer review only
