# Supplementary material for: Perinatal and 2-year neurodevelopmental outcome in late preterm fetal compromise: the TRUFFLE 2 randomised trial protocol
Source: BMJ Open. 2022 Apr 15;12(4):e055543. doi: 10.1136/bmjopen-2021-055543 (PMC9014041; doi:10.1136/bmjopen-2021-055543)
Supplement: Reviewer comments [file bmjopen-2021-055543.reviewer_comments.pdf]

PEER REVIEW HISTORY

BMJ Open publishes all reviews undertaken for accepted manuscripts. Reviewers are asked to complete a checklist review form (http://bmjopen.bmj.com/site/about/resources/checklist.pdf) and are provided with free text boxes to elaborate on their assessment. These free text comments are reproduced below.

ARTICLE DETAILS

**TITLE (PROVISIONAL)**	Perinatal and 2 year neurodevelopmental outcome in late preterm fetal compromise: the TRUFFLE 2 Randomised Trial Protocol
**AUTHORS**	Mylrea-Foley, Bronacha; Arabin, Birgit; Berger, Astrid; bergman, Eva; Bhide, Amarnath; Bilardo, Caterina; Breeze, Andrew; Brodszki, Jana; Calda, Pavel; Cannings-John, Rebecca; Cesari, Elena; Cetin, I; Dall'Asta, Andrea; Ebbing, Cathrine; Ferrazzi, Enrico; Frusca, Tiziana; Goodier, Jenny; Gyselaers, Wilfried; Hecher, Kurt; Klaritsch, Philipp; Krofta, Ladislav; Lindgren, Peter; Lobmaier, Silvia; Marlow, Neil; Marsal, K; Maruotti, Giuseppe; Mecacci, Federico; Mullins, E; Myklestad, Kirsti; Napolitano, Potter, Claire; Raffaele; Prefumo, Federico; Raio, Luigi; Richter, Jute; Sande, Ragnar; Stampalija, Tamara; Thornton, Jim; Townson, Julia; Valensise, Herbert; Visser, Gerard; Wee, Ling; Wolf, Hans; Lees, Christoph

This article was not externally reviewed at BMJ Open. Protocols that have been independently assessed prior to submission to BMJ Open are usually fast-tracked to publication on the grounds that further substantial changes will not be possible. This independent assessment will usually be external, independent review for both a substantial grant award from a non-commercial or government funder and ethics approval.
